# Efficacy and safety of MP1032 plus standard-of-care compared to standard-of-care in hospitalised patients with COVID-19: a multicentre, randomised double-blind, placebo-controlled phase 2a trial

**DOI:** 10.1016/j.lanepe.2023.100810

**Published:** 2023-12-06

**Authors:** Petra Sager, Astrid Kaiser, Sara Schumann, Beate Ludescher, Michael Niedermaier, Ivo Schmidt, Katharina Och, Christiane Dings, Thorsten Lehr, Wolfgang Brysch

**Affiliations:** a MetrioPharm Deutschland GmbH, Am Borsigturm 100, 13507, Berlin, Germany; b Saarmetrics GmbH, Starterzentrum 1, Universität des Saarlandes, 66123, Saarbrücken, Germany; c Department of Clinical Pharmacy, Saarland University, 66123, Saarbrücken, Germany; d MetrioPharm AG, Europaallee 41, Zurich 8004, Switzerland

**Keywords:** Host-directed therapy, Anti-inflammatory, Anti-infective, Covid-19, Pandemic preparedness

## Abstract

**Background:**

SARS-CoV-2 infections still have a significant impact on the global population. The existing vaccinations have contributed to reducing the severe disease courses, decreasing hospitalisations, and lowering the mortality rate. However, due to the variability of COVID-19 symptoms, the emergence of new variants and the uneven global distribution of vaccines there is still a great need for new therapy options. One promising approach is provided by host-directed therapies. We assessed here the efficacy and safety of MP1032, a host-directed anti-viral/anti-inflammatory drug in hospitalised patients with moderate to severe COVID-19.

**Methods:**

In a randomised, double-blind, placebo-controlled, Phase IIa study, patients were randomised 2:1 to receive either 300 mg MP032 bid + Standard-of-Care (SoC) or placebo bid + SoC for 28 days. Eligible patients were ≥18 years old, tested positive for SARS-CoV-2, and had moderate to severe COVID-19 symptoms. The study spanned 20 sites in six countries (Bulgaria, France, Hungary, Italy, Romania, Spain), assessing disease progression according the NIAID scale as the primary outcome on day 14. Secondary objectives included disease progression (day 28), disease resolution (days 14 and 28), mortality rate, COVID-19 related parameters and safety. Exposure-response analyses were performed, linking MP1032 to COVID-19 biomarkers (eGFR, D-dimer).

**Findings:**

132 patients were enrolled to receive MP1032 + SoC (n = 87) or placebo + SoC (n = 45). The patients were all white or Caucasian with a mean (median) age of 60.5 (63) years. Overall, only 10 patients were vaccinated, 5 in each group. No significant risk difference of disease progression could be detected between groups on both day 14 (9.8% MP1032 vs. 11.6% placebo) and day 28 with MH common risk differences of −0.276% (95% CI, −11.634 to 11.081; p = 0.962) and 1.722% (95% CI, −4.576 to 8.019; p = 0.592), respectively.

The treatment with MP1032 + SoC was safe and well-tolerated. Overall, 182 TEAEs including 10 SAEs were reported in 53.5% (46/86) of patients of the verum group and in 57.8% (26/45) of patients of the placebo group; the SAEs occurred in 5.8% (5/86) and 6.7% (3/45) of verum and placebo patients, respectively. None of the SAEs was considered as related.

**Interpretation:**

Despite the study's limitation in size and the variation in concurrent SoCs, these findings warrant further investigation of MP1032 as a host-directed anti-viral drug candidate.

**Funding:**

The study was funded by the COVID-19 Horizon Europe work programme and MetrioPharm AG.

**Research in context**

**Evidence before this study**

Prior to commencing this study in December 2020, an exhaustive review of existing literature and clinical trial databases, including PubMed and ClinicalTrials.gov as well as the official websites of the EMA, FDA and WHO was conducted. The search, unrestricted by publication date or language, focused on keywords such as “COVID-19”, “SARS-CoV-2” and therapy thereof, “immune-modulatory therapy of COVID-19” and “overreacting immune response in COVID-19”. This exploration revealed substantial evidence suggesting the role of immunopathological processes in COVID-19, notably the excessive production of reactive oxygen species (ROS; oxidative stress) and cytokines culminating in a cytokine storm and leading to multiple organ failure in severe cases. This understanding underscored an urgent need for therapeutic agents that offer not only anti-viral effects but also anti-inflammatory properties. At this time, the RECOVERY trial highlighted this by showing that intravenous dexamethasone lowered 28-day mortality of patients with severe COVID-19, underscoring the potential of anti-inflammatory treatment. Although development of MP1032 was originally promoted in the field of autoimmune inflammatory diseases, in view of MP1032's proposed mechanism of action, we had conducted initial anti-viral preclinical studies. These *in vitro* studies demonstrated immune-modulatory, self-regulated ROS scavenging and SARS-CoV-2 anti-viral properties, thereby providing a compelling basis for further investigation. In summary, the existing clinical data and our initial preclinical findings established a strong scientific and clinical foundation for advancing research into anti-inflammatory agents in the context of COVID-19.

**Added value of this study**

Our study was the first randomised, double-blind, placebo-controlled clinical trial assessing the effect of oral MP1032 in hospitalised adults with moderate to severe COVID-19 and Standard-of-Care (SoC) treatment. The study was successfully completed. MP1032 was safe and well tolerated. Although limited due to its small size and high variation in SoC treatments, the results based on multivariate time-to-event analyses of the study provide indications towards earlier discharge from hospital and faster recovery. Further, to our knowledge, MP1032 is the only treatment tested so far that has shown a significant effect on the COVID-19 relevant biomarkers eGFR and D-dimer.

**Implications of all the available evidence**

As a host-directed drug, MP1032 provides the possibility to serve as a successful therapy against future SARS-CoV-2 variants, as well as other RNA viruses such as influenza, and to mitigate the course of infections. However, further phase II/III studies are required to confirm the host-directed anti-inflammatory effect of MP1032 in viral infections.

## Introduction

Even after more than three years into the pandemic, the coronavirus disease 2019 (COVID-19), caused by the severe acute respiratory syndrome coronavirus 2 (SARS-CoV-2) still confronts us with new challenges. These include the virus's high mutation rate, the complex and multi-system nature of severe COVID-19, and the variable immune response in different populations.^1^ Additionally, there are logistical issues such as the scalability and equitable global distribution of therapeutic interventions.

As of 22 June 2023, over 768 million confirmed cases and over 6.9 million deaths have been reported globally.^2^ SARS-CoV-2 infection can be asymptomatic or symptomatic with the latter ranging in severity from mild common cold symptoms to critical respiratory illnesses such as acute respiratory distress syndrome and pneumonia. Furthermore, several non-respiratory symptoms like sepsis often complicate the clinical picture of COVID-19 patients.^3^

Although vaccination strategies and several treatment options are in place, SARS-CoV-2 might persist as an endemic virus, perhaps with seasonal epidemic peaks. As the virus has the ability to evolve new variants with unpredictable characteristics, developing host-directed treatments could be a viable strategy to establish a pan-variant-proof drug.^4^^,^^5^

Host-directed therapies offer several potential advantages, including a broader spectrum of activity across diverse viral species and strains, a reduced likelihood of resistance emergence, and the potential for synergistic effects when combined with existing anti-viral drugs or vaccines.^6^ MP1032 belongs to these host-directed therapies that prevent an overreaction of the immune system independent of the pathogen while also inhibiting virus replication.

MP1032 is a phase-pure anhydrous polymorph of 5-amino-2,3-dihydro-1,4-phthalazinedione sodium salt^7^ that is orally available with good stability. The physiological action of MP1032 is based on a dual mode of action, targeting two important mechanisms: i) immune system overactivation and ii) viral replication both result in a strong dose-dependent SARS-CoV-2 anti-viral effect, which is comparable to remdesivir.^8^ MP1032 leads to a reduced concentration of pathologically increased pro-inflammatory cytokines (e.g., IL-6 and TNF-α) without completely blocking them, which was shown *in vivo* and in *vitro*. Furthermore, it suppressed virus replication dose-dependently *in vitro*.^8^ In combination with oral availability, a favourable safety profile and high stability at ambient temperatures, MP1032 is the ideal candidate for early treatment of COVID-19, which can also be used in high-risk patients. In this study, we have therefore tested the hypothesis that MP1032 can contribute to the improvement of COVID-19 patients.

As part of the COVID-19 Horizon Europe work programme (Project No.: 101046182), we present here the results of an exploratory phase IIa clinical trial of the host-directed drug candidate MP1032, assessing the effects of this compound as an add-on treatment to Standard-of-Care (SoC) in hospitalised COVID-19 patients.

## Methods

### Study design and participants

This Phase IIa study evaluated the efficacy of 300 mg MP1032 bid (twice a day) + SoC vs. placebo + SoC in hospitalised patients with moderate to severe COVID-19 (EudraCT-No.: 2021-000344-21, IND 153604). The study was randomised, double-blind, and placebo-controlled, with enrollment at 20 Independent-Ethics-Committee-approved sites across six countries (Bulgaria, France, Hungary, Italy, Romania, Spain). A list of all participating sites, the in-and exclusion criteria and the study protocol are published on clinicaltrials.gov (NCT04932941).

Patients aged 18 years or older with a positive SARS-CoV-2 test (RT-PCR assay or equivalent test) and symptoms suggestive of moderate or severe systemic illness were eligible for enrollment, while those with asymptomatic or mild COVID-19, or critical COVID-19 at screening, were excluded.

The proportion of patients with at least 1 major protocol deviation in both groups was similar (20.7% of patients in the MP1032 plus SoC group and 24.4% of patients in the placebo plus SoC group). None of the deviations led to the exclusion from the ITT set.

Moderate COVID-19 was characterised by a respiratory rate ≥20 breaths per minute, SpO_2_ >93%, and a heart rate ≥90 beats per minute, with no signs indicative for severe or critical COVID-19. Severe COVID-19 was characterised by a respiratory rate ≥30 breaths per minute, SpO_2_ ≤93%, heart rate ≥125 beats per minute, partial pressure of oxygen/FiO_2_ <300, or diagnosed with acute respiratory distress syndrome (according to the Berlin definition); but no criteria met for critical COVID-19.

### Randomisation and masking

The randomisation of patients to treatment groups was performed centrally by an Interactive Web-Response System (IWRS) using a randomisation scheme that was produced by an unblinded, independent statistician. The IWRS assigned a randomisation number and two kit numbers (for day 1 and day 14 visits) to each patient. The study drug kits contained the respective blinded treatment available at the study site. The randomisation was performed stratified considering the baseline COVID-19 severity (moderate and severe) and age-class (≤65 years and >65 years) and in blocks.

MP1032 hard gelatine capsules of 50 mg and placebo capsules were supplied in identical blister packaging and were colour and size-matched, with blinded labelling. All patients, investigators, and all staff involved in the conduct of the study (including sponsor and CRO) were blinded to the treatment.

### Procedures

After a screening period of up to 7 days patients, were treated orally with 300 mg MP1032 bid + SoC or placebo bid + SoC for 28 consecutive days, supported with a follow-up period until day 60 ( ± 3 days). Patients who were discharged before day 28 received the remaining blinded study drug kit(s) to continue treatment at home.

All patients received selected SoC that was used in accordance with the hospital's SoC procedures and that could have included drugs under an emergency use authorisation. Clinical data, concomitant medication, adverse events, and COVID-19-related clinical status on the National Institute Allergy and Infectious Disease (NIAID)-8 point scale were collected daily until day 14, and at days 21, 28 (end of treatment), 60 (end of follow-up), and at the day of discharge. Blood samples for biomarkers were collected on days 1, 7, 14, 21, 28 and 60. Plasma concentrations of MP1032 were determined at prespecified time points (on days 1 and 7) in a subgroup of patients using a validated HPLC method. Safety assessments started at screening with the collection of medical and surgery history, demographics, prior medication, and an electrocardiogram (ECG). In scope of the scheduled visits, physical examinations and laboratory assessments were performed, vital signs were collected, and patients were actively asked for adverse events (AEs). An independent data monitoring committee (DMC) reviewed accumulating study data at regular intervals throughout the study to ensure the safety of patients and the integrity of the overall study conduct (independent from the sponsors and clinical investigators).

### Outcomes

The primary objective was to measure the effect of MP1032 + SoC vs. placebo + SoC on Day 14 according to WHO Master Protocol using the NIAID 8-point scale (NIH Master Protocol, ACTIV-1, 2020): (1) death; (2) hospitalised, on invasive ventilation; (3) hospitalised, on non-invasive ventilation; (4) hospitalised, requiring suppl. oxygen; (5) hospitalised, not requiring suppl. oxygen, but requiring medical care; (6) hospitalised, no longer requiring ongoing medical care; (7) not hospitalised, limitations on activities, and/or requiring home oxygen; (8) not hospitalised, no limitations on activities. The secondary objectives included disease progression on day 28, disease resolution (NIAID score ≥6) at day 14 and day 28, mortality rate and specific COVID-19 related characteristics. Safety outcomes as cumulative incidence of treatment emergent adverse events (TEAEs), vital signs, clinical laboratory parameters and physical examination findings were also assessed. Additionally, in case of deaths, other SAEs, and certain other clinically significant AE, case narratives were prepared.

Exploratory objectives included duration of intensive care unit (ICU) treatment, duration of extracorporeal membrane oxygenation (ECMO), time to recovery from COVID-19 symptoms, health-related quality of life and biomarker levels.

### Statistical analysis

For determination of sample size, the primary efficacy endpoint “proportion of patients with disease progression at day 14” was assumed to be 10% in the 300 mg MP1032 bid + SoC treatment group (Arm A) and 30% in the placebo bid + SoC treatment group (Arm B). Using the Chi-square test with type I error alpha = 10% two-sided for this proof-of-concept study and a 2:1 randomisation ratio, 114 patients (76 in Arm A and 38 in Arm B) were required to achieve a statistical power of 83%. Considering about 5% early study terminations, the necessary sample size for randomisation resulted in 120 patients in total (80 in Arm A and 40 in Arm B). As the early study termination rate was higher than the estimated 5%, the number of randomised patients was increased during the study to ensure 114 evaluable patients at day 14. Sample size was estimated using nQuery 8, version 8.6.1.0.

The Intention-To-Treat (ITT) set was used for the analysis of efficacy, summary of demographics, and baseline characteristics. The ITT set included all randomised patients, irrespective of any deviation from the protocol or premature discontinuation from the study drug or withdrawal from study. The treatment group assignment was designated according to initial randomisation. The Safety Set (SS) included all patients who received at least one dose of the study drug and was used for the analysis of the safety. The pharmacokinetic set (PKS) included all patients who received the active study drug and had at least one evaluable plasma concentration after the day 1 dose. The Per-Protocol Set (PPS) was used for supportive analyses of efficacy and included all patients from the ITT set who received at least 1 dose of the study drug and who had no major protocol deviations affecting the efficacy assessments.

The main estimand for the primary efficacy endpoint was defined as a binary variable indicating disease progression at day 14 and is compared between treatment groups based on the common risk difference resulting from the Mantel-Haenszel (MH) test considering the four strata out of the combination of the two randomisation stratification factors: disease severity (moderate vs. severe) and age class (≤65 years vs. >65 years). Different imputation methods were used to prevent bias as far as possible with the remaining bias directing towards a poorer outcome for the patient. Missing data due to study termination prior to day 14 were imputed by multiple imputation using information from similar patients of the same treatment group assuming a “missing at random” mechanism. Missing postbaseline data for patients randomised but not treated were imputed by the respective baseline value (baseline observation carried forward). Intermediate missing data of day 14 (i.e., NIAID assessments prior to day 14 and at day 28 were available, but only day 14 was missing) were imputed by last observation carried forward. A 95% CI was provided for the risk difference.

A second estimand for the primary efficacy endpoint was defined and evaluated similarly to the main estimand but with the only difference that the intercurrent event of study drug discontinuation due to AE or due to lack of efficacy is handled using the composite strategy as “disease progression”, but the intercurrent event of study drug discontinuation due to other reasons is still handled with the treatment-policy strategy. The imputation strategy as described for the main estimand was used for the second estimand as well. Similar sensitivity analyses were applied for the main and the second estimand.

For the key secondary efficacy endpoints on disease progression and disease resolution similar tests and estimand definitions were used.

Safety data were summarised descriptively by seriousness, severity, relationship to study medication, outcome and duration. AEs and medical history were coded using MedDRA version 24.0 Analysis and summaries were derived using SAS (Version 9.4, SAS Institute Inc.).

### Exposure—response analysis

An exposure-response analysis was performed to establish a pharmacokinetic (PK) model of MP1032, which was linked to the biomarkers D-dimer and estimated glomerular filtration rate (eGFR), as well as to the outcome “discharge from hospital” recorded as a score of 7 or higher on the NIAID 8-point scale using the non-linear mixed effects modelling approach within the software NONMEM (version 7.4.3, ICON Development Solutions, Ellicott City, MD, USA). A detailed method section is provided in the supplement.

### Role of the funding source

This study was mainly funded by the COVID-19 Horizon Europe work programme (Project No.: 101046182). The funder of the study had no role in study design, data collection, data analysis, data interpretation, or writing of the report. The study was also funded by the study sponsor MetrioPharm AG.

## Results

### Patients

**Fig. 1:**
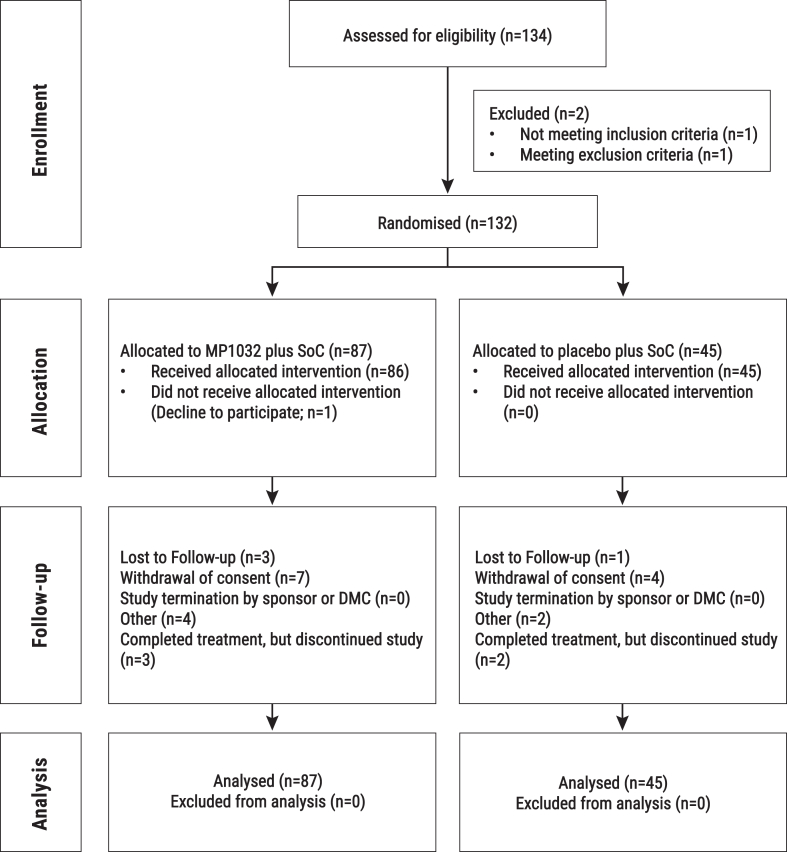
**CONSORT flow-chart of patients disposition**. SoC: Standard-of-Care, DMC: Data Monitoring Committee.

Out of the 134 screened patients, 132 patients were randomly assigned between October 19, 2021 and June 1, 2022 to receive either MP1032 + SoC (n = 87) or the placebo + SoC (n = 45). Out of the 87 patients randomised to the MP1032 + SoC group, 86 patients (98.9%) were dosed with the study drug. All 45 patients randomised to the placebo + SoC group were dosed with placebo. By day 14, 121 out of 132 patients (91.7%) had completed treatment, with 80 patients (92.0%) in the MP1032 + SoC group and 41 patients (91.1%) in the placebo + SoC group. The most common reasons for discontinuation of treatment before day 14 were withdrawal of consent (3.8%) and adverse events (3.0%). Overall, 107 patients (81.1%) completed both treatment and follow-up, with 70 patients (80.5%) in the MP1032 + SoC group and 37 patients (82.2%) in the placebo + SoC group (Fig. 1).

The SoC treatments used in this study were generally balanced between the two groups, with oxygen being the most common treatment used in 98.9% of the patients in the MP1032 group vs. 100% of the patients in the placebo group.

Other common treatments included dexamethasone (62.15% in the MP1032 + SoC group vs. 60.0% in the placebo + SoC group), sodium chloride (51.7% vs. 60.0%), paracetamol (36.8% vs. 42.2%), methylprednisolone (35.6% vs. 35.6%), ceftriaxone (33.3% vs. 40.0%), pantoprazole (36.8% vs. 33.3%), and ascorbic acid (33.3% vs. 40.0%).

The use of anti-virals for systemic use was also balanced between both groups (39.1% in the MP1032 + SoC vs. 40.0% in the placebo + SoC), with remdesivir being the most commonly administered anti-viral (26.4% (MP1032 + SoC) vs. 33.3% (placebo + SoC)).

Pharmacokinetic samples could only be collected from a limited cohort of six patients due to COVID-19 pandemic workload, limited staff and time constraints. Details regarding the samples for the pharmacokinetic analysis are provided in the supplements.

The mean age of patients was 59.6 years (median age 63 years), with 58.3% of the patients being male. All patients were white or Caucasian. All patients had a baseline COVID-19 severity of moderate or severe. At baseline, 80.3% of patients had a NIAID score of 4, while 18.2% had a score of 5, and 1.5% had a score of 3 (Table 1).

**Table 1:** Demographics, baseline and further characteristics of patients (ITT set).

Characteristics	MP1032 + SoC (n = 87)	Placebo + SoC (n = 45)	Total (n = 132)
Age, years, mean ± SD	59.6 ± 13.8	62.3 ± 14.15	60.5 ± 13.9
Sex, n (%)			
Male	51 (58.6)	26 (57.8)	75 (58.3)
Female	36 (41.4)	19 (42.2)	57 (41.7)
Race, n (%)			
American Indian or Alaska Native	0	0	0
Asian	0	0	0
Black or African American	0	0	0
Native Hawaiian or other pacific Islander	0	0	0
Other	0	0	0
White or Caucasian	87 (100)	45 (100)	132 (100)
Ethnicity, n (%)			
Hispanic or Latino	5 (5.7)	1 (2.2)	6 (4.5)
Not Hispanic or Latino	79 (90.8)	44 (97.8)	123 (93.2)
Not reported/specified	3 (3.4)	0 (0.0)	3 (2.3)
BMI (kg/m^2^), mean ± SD	29.17 ± 5.45	28.5 ± 4.6	28.95 ± 5.17
Coexisting conditions, n (%)			
Hypertension	45 (51.7)	19 (42.2)	64 (48.5)
Type 2 Diabetes	6 (6.9)	3 (6.7)	9 (6.8)
Obesity	11 (12.6)	4 (8.9)	15 (11.4)
Baseline COVID-19 severity, n (%)			
Moderate	42 (48.3)	21 (46.7)	63 (47.7)
Severe	45 (51.7)	24 (53.3)	69 (52.3)
Baseline NIAID score, n (%)			
2	0	0	0
3	1 (1.1)	1 (2.2)	2 (1.5)
4	70 (80.5)	36 (80)	106 (80.3)
5	16 (18.4)	8 (17.8)	24 (18.2)
6, 7, 8	0	0	0
Elapsed time since COVID-19 diagnosis, days, mean ± SD	3.4 ± 3.85	2.9 ± 2.56	3.2 ± 3.47
Elapsed time since COVID-19 diagnosis (≤3 days), n (%)	57 (65.5)	29 (64.4)	86 (65.2)
Pneumonia, n (%)	82 (94.3)	43 (95.6)	125 (94.7)
ARDS, n (%)	16 (18.4)	4 (8.9)	20 (15.2)
Another diagnosis/etiology for illness, n (%)	8 (9.2)	4 (8.9)	12 (9.1)
Abnormal chest image, n (%)	82 (94.3)	44 (97.8)	126 (95.5)
Hypercoagulability, n (%)	15 (17.2)	13 (28.9)	28 (21.2)
Vaccinated, n (%)	5 (5.7)	5 (11.1)	10 (7.8)
Anti-virals for systemic use, n (%)	34 (39.1)	18 (40.0)	52 (39.4)
Remdesivir	23 (26.4)	15 (33.3)	38 (28.8)
Molnupiravir	6 (6.9)	2 (4.4)	8 (6.1)
Favipiravir	5 (5.7)	1 (2.2)	6 (4.5)
Aciclovir	1 (1.1)	2 (4.4)	3 (2.3)
Bictegravir	1 (1.1)	0	1 (0.8)
Cilgavimab	1 (1.1)	0	1 (0.8)
Emtricitabine	1 (1.1)	0	1 (0.8)
Tenofovir Alafenamide	1 (1.1)	0	1 (0.8)
Tixagevimab	1 (1.1)	0	1 (0.8)

Age was collected at Screening and COVID-19 severity at day 1. Percentages were collected using the number of patients included in the ITT Set. BMI: Body Mass Index, NIAID: National Institute Allergy and Infectious Disease, ARDS: acute respiratory distress syndrome.

Most patients had at least one coexisting medical diagnosis at enrolment, with hypertension (48.5%), obesity (11.4%), and type 2 diabetes (6.8%) being the most common.

The mean elapsed time since COVID-19 diagnosis was 3.2 days and 65.2% of patients were randomised within three days. The majority of patients presented with pneumonia (94.7%) and abnormal chest images (95.5%) and all patients exhibited symptoms, with the most frequent being cough (97.0%), fatigue (93.2%), and fever ≥38 °C (78.8%). The proportion of patients with acute respiratory distress syndrome (ARDS) at baseline was higher in the MP1032 + SoC group (18.4%) compared to the placebo + SoC group (8.9%). Other symptoms were reported in ≤50.0% of patients. Overall, only 10 patients were vaccinated, 5 patients in each group (corresponding to 5.7% in the MP1032 + SoC group and 11.1% in the placebo + SoC group; Table 1).

Analysing the ITT set, the main estimand of the primary analysis revealed no significant risk difference of disease progression on day 14 between MP1032 + SoC and placebo + SoC (9.8% vs. 11.6%, respectively), with a MH common risk difference of −0.276% (95% CI, −11.634 to 11.081; p = 0.962).

The results of the second estimand analysis were similar to the primary estimand results. The proportion of patients with disease progression at day 14 was 10.8% in the MP1032 + SoC group vs. 15.9% in the placebo + SoC group (MH common risk difference of −3.772% (95% CI, −16.537 to 8.994; p = 0.562) (Table 2).

**Table 2:** Primary and secondary outcomes in the ITT sets.

Endpoint	MP1032 plus SoC (n = 87)	Placebo plus SoC (n = 45)	Statistics (MH CI = Mantel-Haenszel common risk difference 95% CI and MH (%) = common risk difference (%))
**Primary efficacy endpoint**			
Patients with disease progression on day 14, n (%%)	Primary analysis: Main estimand: 8 (9.8) Second estimand: 9 (10.8)	Primary analysis: Main estimand: 5 (11.6) Second estimand: 7 (15.9)	MH CI −11.634; 11.081 p = 0.962 MH (%) −0.276 MH CI −16.537; 8.994 p = 0.962 MH (%) −3.772
**Secondary efficacy endpoints**			
Patients with disease progression on day 28, n (%)	Primary analysis: Main estimand: 2 (2.5) Second estimand: 4 (4.9)	Primary analysis: Main estimand: 1 (2.4) Second estimand: 3 (7.1)	MH CI −4.576, 8.019 p = 0.592 MH (%) 1.722 MH CI −9.784, 8.422 p = 0.883 MH (%) −0.681
Patients with disease resolution on day 28, n (%)	Primary analysis: Main estimand: 77 (97.5) Second estimand: 77 (95.1)	Primary analysis: Main estimand: 40 (97.6) Second estimand: 39 (92.9)	MH CI −8.019, 4.576 p = 0.592 MH (%) −1.722 MH CI −8.422, 9.784 p = 0.883 MH (%) 0.681
Patients with disease resolution on day 14, n (%)	69 (84.1)	31 (72.1)	MH CI −4.027, 26.769 p = 0.128 MH (%) 11.371
Patients who died by day 14, n (%)	1 (1.2)	1 (2.3)	MH CI −9.981, 7.981 p = 0.820 MH (%) −1.00
Patients who died by day 28, n (%)	2 (2.4)	1 (2.4)	MH CI −10.983, 9.431 p = 0.880 MH (%) −0.776
Patients who died by day 60, n (%)	3 (3.8)	2 (4.9)	MH CI −13.936, 10.209 p = 0.758 MH (%) −1.863
Change of NIAID Score at day 28	Primary analysis: LS mean (SE): 3.542 (0.130)	Primary analysis: LS mean (SE): 3.612 (0.174)	LS mean difference: −0.069, 95% CI −0.488, 0.350 p = 0.746
Change of NIAID Score at day 14	LS mean (SE): 2.987 (0.174)	LS mean (SE): 2.543 (0.242)	LS mean difference: 0.444, 95% CI −0.136, 1.023 p = 0.132
Patients requiring Invasive Mechanical Ventilation/ECMO or died n (%) at day 14	1 (1.2)	1 (2.3)	MH CI −6.273, 4.244 p = 0.675 MH (%) −1.015
Patients requiring Invasive Mechanical Ventilation/ECMO or died n (%) at day 28	2 (2.5)	1 (2.4)	MH CI −5.784, 6.405 p = 0.919 MH (%) 0.919

According to the treatment groups. Data are n (%), except where otherwise stated. SoC: Standard-of-Care, MH: Mantel-Haenszel, LS: least square, ECMO: extracorporeal membrane oxygenation, NIAID: National Institute of Allergy and Infectious Diseases, SE: standard error.

**Fig. 2:**
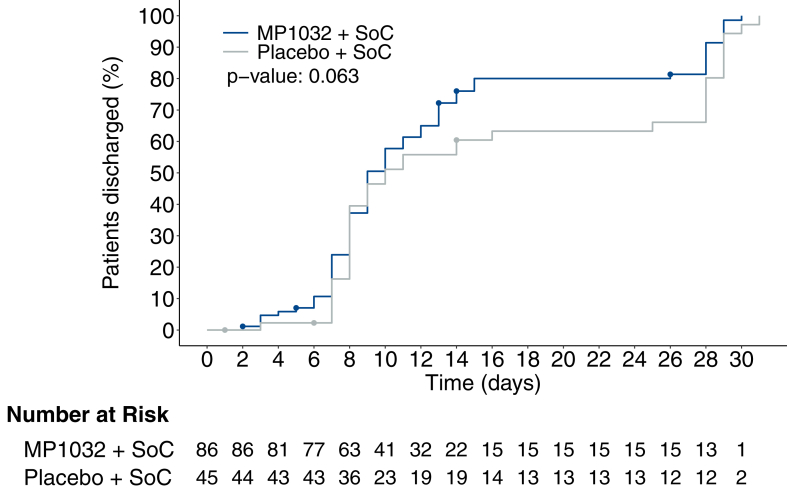
**Time to discharge of patients**. Kaplan–Meier estimates for time to discharge of patients after treatment start with MP1032 + SoC (blue) or placebo + SoC (grey). Dots represent censored data.

The main estimand analysis of the secondary efficacy assessment did not reveal any significant difference in the risk of disease progression on day 28 between the two groups with an MH common risk difference of 1.722% (95% CI, −4.576 to 8.019; p = 0.592; Table 2). The risk of death was not significantly different between the groups (Table 2), however, there was a corresponding relative risk reduction of 23.0% in the MP1032 + SoC group on day 60 (data not shown). Additionally, median duration of ICU treatment (5 patients out of each group) within both periods of 28 days and 60 days was shorter in the MP1032 + SoC group (8.5 days) compared to the placebo + SoC group (12.5 days) and there was a tendency towards earlier discharge or reaching NIAID 7 (Fig. 2).

Showing faster recovery, patients in the MP1032 + SoC group were discharged sooner from hospital, indicated by reaching NIAID ≥7 (NIAID 7 = not hospitalised, limitation on activities and/or requiring home oxygen; Fig. 2). Post-hoc stratification by anti-viral medication showed similar results with an additional beneficial effect from anti-viral SoC medication (including remdesivir; Supplementary Figure S1).

Exploratory findings could be quantified more precisely by a multivariate time-to-event (TTE) analysis, where MP1032 plasma concentrations showed a significant (p < 0.05) increase in the probability of discharge, which lagged behind the exposure with a mean transit time of 8.3 days. Additionally, three significant covariates (p < 0.01) were identified: i) Higher baseline NIAID value goes along with better chances for earlier discharge, ii) Older patient age and iii) Higher aspartate aminotransferase (ASAT) values are associated with a higher probability of a later discharge. In a post-hoc exposure-response modelling analysis, the effect of MP1032 + SoC on the biomarkers eGFR and D-dimer revealed a significant effect of MP1032 plasma concentrations resulting in a faster increase to normal range eGFR levels (p < 0.001) and a significant decrease of D-dimer levels compared to placebo (p < 0.001) (Supplementary Figure S2). Further details are provided in the supplementary results section.

Overall, MP1032 was safe and well tolerated. 182 TEAEs were reported in scope of the study, including 10 SAEs. Forty-six patients (53.5%) treated with MP1032 + SoC and 26 patients (57.8%) treated with placebo + SoC reported at least one TEAE, predominantly classified as mild or moderate in severity. In only 5.8% of the patients treated with MP1032 + SoC (n = 5) serious TEAEs were reported compared with 6.7% (n = 3) of placebo + SoC treated patients. Serious TEAEs resulted in death for 3 patients (3.5%) treated with MP1032 + SoC and for 2 patients (4.4%) treated with placebo + SoC. None of the reported serious TEAEs was considered related.

The proportion of patients with non-serious TEAEs considered as related was higher in the MP1032 + SoC group (25.6%) compared with the placebo + SoC group (15.6%). The most frequently reported related TEAEs (reported in ≥5% of patients) in the MP1032 + SoC group included hypertriglyceridaemia, increased alanine aminotransferase, and diarrhoea. The proportion of patients with TEAEs leading to permanent discontinuation of the study drug was lower in the MP1032 + SoC group than in the placebo + SoC group (5.8% vs. 8.9%, respectively). An overview on the most important numbers received from the safety assessment is provided in Table 3.

**Table 3:** Frequency of treatment emergent adverse events.

Adverse events	MP1032 + SoC (n = 86)	Placebo + SoC (n = 45)	All (n = 131)
**Any adverse events**	114/46 (53.5)	68/26 (57.8)	182/72 (55.0)
Most common adverse events^a^			
Alanine aminotransferase increased	7/7 (8.1)	6/6 (13.3)	13/13 (9.9)
Blood glucose increased	1/1 (1.2)	3/3 (6.7)	4/4 (3.1)
Hypertriglyceridaemia	11/9 (10.5)	4/4 (8.9)	15/13 (9.9)
Hypercholesterolaemia	5/3 (3.5)	2/2 (4.4)	5/5 (3.8)
Hyperglycaemia	3/3 (3.5)	4/4 (8.9)	7/7 (5.39)
Diarrhoea	5/5 (5.8)	0/0 (0.0)	5/5 (3.8)
**Any related adverse events**^b^	54/22 (25.6)	21/7 (15.6)	75/29 (22.1)
**Adverse events leading to permanent discontinuation of study drug**	5/5 (5.8)	4/4 (8.9)	9/9 (6.9)
**Any serious adverse events**	7/5 (5.8)	3/3 (6.7)	10/8 (6.1)
**Any related serious adverse events**	0/0 (0.0)	0/0 (0.0)	0/0 (0.0)
**Deaths**	3/3 (3.5)	2/2 (4.4)	5/5 (3.8)

Numbers indicate: number of adverse events/number of patients (% of patients).

a Shown are all treatment emergent adverse events occurring in at least 5% of patients in any of the treatment groups or overall.

b Treatment emergent adverse events that were considered as related by the investigator.

## Discussion

Here, we present the findings of a randomised, double-blind, placebo-controlled phase IIa trial conducted across multiple centres and evaluating the efficacy and safety of the host-directed anti-viral/anti-inflammatory compound MP1032 in hospitalised patients with moderate to severe COVID-19. Patients were assigned to receive either MP1032 or placebo in combination with SoC. No statistically significant differences were observed in both, the primary and secondary endpoints between patients receiving MP1032 + Soc or placebo + SoC. However, patients treated with MP1032 + SoC exhibited a lower corresponding relative mortality compared to those receiving placebo + SoC. Furthermore, our findings demonstrate that in terms of early recovery from the disease and time to discharge, MP1032 exhibits comparable efficacy to remdesivir.^9^^,^^10^ Moreover, the efficacy of MP1032 was enhanced when used in combination with anti-viral agents, including but not limited to remdesivir. While anti-viral treatments generally target specific viruses, MP1032 is classified as a host-directed therapy.^8^ Hence, the combination of MP1032 with anti-viral medication might have additive or synergistic effects in the treatment of patients with various viral infections.

A multivariate TTE analysis revealed a significant effect of MP1032 exposure on the probability of discharge, indicating that higher MP1032 plasma concentration increases the probability of discharge (compared with placebo + SoC) with a mean transit time of 8.3 days. Additionally, older age was identified as a covariate for later discharge. This is in line with previous findings showing that elderly patients are more susceptible to complication of SARS-CoV-2 infection due to an altered immune system response.^11^ Furthermore, Herta and Berg reported that elevated liver function test results are linked to a more severe disease outcome, which is supported by our finding that increased ASAT values correlate with a later discharge.^12^

To further investigate the effects of MP1032 + SoC, a post-hoc analysis was conducted on eGFR and D-dimer biomarkers that demonstrated a significant effect of MP1032 exposure resulting in a faster increase of eGFR levels to normal range and a significant faster decrease of D-dimer levels compared to placebo. Reduced eGFR is associated with chronic kidney disease^13^ and it has been demonstrated that eGFR is a strong prognostic marker for a severe outcome and higher mortality of COVID-19 patients, especially in older patients and in patients with co-morbidities.^14^^,^^15^ In addition, elevated levels of D-dimer, an indirect marker of thrombosis, are recognized to be linked with a prothrombotic condition, ultimately contributing to a negative clinical outcome in COVID-19 infection.^16^^,^^17^ A significant decrease in D-dimer levels in patients receiving MP1032 + SoC suggests the potential to mitigate the risk of cardiovascular complications by MP1032 treatment.

Although further data are needed, altogether, these findings suggest the potential of MP1032 + SoC as a treatment option for COVID-19 patients. Considering MP1032 is classified as a host-directed therapy,^8^ there is a likelihood that MP1032 will exhibit comparable efficacy against other RNA viruses, including future variants of SARS-CoV-2 and influenza viruses. These properties are currently under further investigation and would make MP1032 a promising option for a broad first-line therapeutic and prophylactic treatment option to protect at-risk populations against new, emerging viruses.

In interpreting the results of our exploratory clinical phase II trial, several key limitations must be acknowledged. First, the small number of patients enrolled in the trial and the variability within this cohort limits the statistical power. Second, the high degree of variation in concomitant SoC treatments across patients presents a substantial confounding factor. This heterogeneity makes it challenging to gauge the discrete impact of the investigational drug on certain clinical outcomes. These limitations underscore the need for a more controlled, larger-scale study, potentially on mixed-cause respiratory viral infections that can yield more statistically reliable data and, ideally, control better for concurrent therapeutic interventions. However, the broad utility of MP1032 as host-based therapy could potentially provide a valuable option in future real-world pandemic scenarios, which will invariably involve different countries, medical systems and standard treatment regimes.

## Contributors

AK, PS, BL, MN, IS, WB, KO, CD and TL had full access to all of the data in the study and take responsibility for the integrity of the data and the accuracy of the data analysis.

SS, WB and IS administered the funding project.

WB and AK decided to publish the paper.

PS, AK, WB, BL, MN and SS provided input on the trial design.

BL, SS, AK, WB and PS were responsible for acquisition, analysis, and interpretation of data.

PS, AK and WB drafted the manuscript.

SS, BL, IS, KO, CD and TL critically revised the manuscript.

PS, AK, IS, CD, KO and TL worked on visualization of the data.

TL, KO and CD contributed to statistical analysis.

WB gave valuable suggestions for data analysis.

All authors were not precluded from accessing data in the study, and they accept responsibility to submit for publication.

## Data sharing statement

Clinical results data and related analyses are openly accessible via clinicaltrials.gov together with the study protocol and the statistical analysis plan. Individual participant data collected will not be shared publicly.

## Declaration of interests

The authors’ affiliations are as follows:

P.S., A.K., S.S., B.L. M.N., I.S. are employees of MetrioPharm Deutschland GmbH.

W.B. is employer, co-founder, and CSO of MetrioPharm AG and CMO of MetrioPharm GmbH.

K.O., C.D. and T.L. are employees of Saarmetrics GmbH and are also members of Department of Clinical Pharmacy of University of Saarland, Germany.

## Appendix ASupplementary data

Final Supplement

Protocol
