# Supplementary material for: Efficacy and safety of MP1032 plus standard-of-care compared to standard-of-care in hospitalised patients with COVID-19: a multicentre, randomised double-blind, placebo-controlled phase 2a trial
Source: Lancet Reg Health Eur. 2023 Dec 6;37:100810. doi: 10.1016/j.lanepe.2023.100810 (PMC10704330; doi:10.1016/j.lanepe.2023.100810)
Supplement: Final Supplement [file mmc1.pdf]

Supplementary Document

Efficacy and safety of MP1032 plus standard-of-care compared to standard-of-care in hospitalised patients with COVID-19: a multicentre, randomised double-blind, placebo-controlled phase 2a trial

Petra Sager[1*], Astrid Kaiser[1], Sara Schumann[1], Beate Ludescher[1], Michael Niedermaier[1], Ivo Schmidt[1], Katharina Och[2,3], Christiane Dings[2,3], Thorsten Lehr[2,3], Wolfgang Brysch[1*4]

[1] MetrioPharm Deutschland GmbH, Am Borsigturm 100, 13507 Berlin, Germany
[2] Saarmetrics GmbH, Starterzentrum 1, Universität des Saarlandes, 66123 Saarbrücken, Germany
[3] Department of Clinical Pharmacy, Saarland University, 66123 Saarbrücken, Germany
[4] MetrioPharm AG, Europaallee 41, 8004 Zurich, Switzerland

Table of Contents

Table of Contents 1
Supplementary Methods 2
Pharmacokinetic sampling 2
Pharmacometric analysis 2
Pharmacokinetic modelling 2
Time-to-event modelling 2
Biomarker modelling 3
Supplementary Results 3
Pharmacokinetics of MP1032 3
Time-to-event analysis 3
Biomarker analysis 3
Supplementary Table 5
Supplementary Figures 6
Supplementary References 11

Supplementary Methods

Pharmacokinetic sampling

PK samples were obtained from four verum and two placebo patients in the presented study (MP1032-CT05). In addition, PK data had previously been collected in three studies conducted in healthy volunteers or patients with psoriasis (MP1032-CT01, MP1032-CT02, MP1032-CT04):

In study MP1032-CT01, healthy volunteers received either single dose from 50 to 600 mg and PK samples were drawn at 0 min (predose), 30 min, 60 min and 90 min, 2 h, 3 h, 4 h, 6 h, 8 h, 12 h, 16 h and 24 h after dose (MP1032-CT01-SD) or they received multiple dose of 100 mg or 300 mg bid over 7 days and PK samples were drawn predose and additionally on day 1 and 7 at 10 min, 20 min, 30 min, 45 min, 60 min, 90 min, 2 h, 3 h, 4 h and 8 h post dose (MP1032-CT01-MD).

In study MP1032-CT02, adult patients with moderate to severe chronic plaque psoriasis received either 100 mg MP1032 bid over 42 days or placebo bid over 42 days. PK samples of MP1032 were drawn on day 1 at 15 min, 30 min, 60 min, 120 min post dose. Additional PK samples were taken predose on day 15 and 29.

In study MP1032-CT04, patients with moderate to severe chronic plaque psoriasis received either 150 mg MP1032 bid, 300 mg MP1032 bid or placebo bid over 12 weeks. PK samples of MP1032 were drawn in subgroup of patients on day 1 and day 84 predose (morning dose), 15 min, 30 min, 60 min, 120 min post morning dose.

In study MP1032-CT05, hospitalized patients with moderate to severe COVID-19 received either 300 mg MP1032 bid + SoC or placebo bid + SoC over a treatment period of 28 days. PK samples were collected from four patients receiving MP1032 and from 2 patients receiving placebo. Samples were drawn on day 1 and day 7 pre-dose, 10 min, 20 min, 30 min, 60 min, 120 min post dose, 8 h before next dose and 24 hours before the next morning dose (if applicable).

Pharmacometric analysis

Pharmacometric analyses were performed using non-linear mixed-effects modelling techniques implemented in the software NONMEM (Version 7.4.3, ICON Development Solutions, Ellicott City, MD, USA). For parameter estimation, the first-order condition estimation algorithm with interaction (FOCE-I) was used for the PK and biomarker analysis and the Laplacian method for time-to-event modelling.[1] For the PK and biomarker analysis inter-individual variability (IIV) was modelled using exponential random effects models. Model selection criteria were (I) significant reduction in NONMEM objective function value (OFV), (II) adequate goodness-of-fit plots such as observations vs. model predictions and visual predictive checks (VPCs),[2] and (III) precise estimation of model parameters.[3]

All models were developed in a stepwise procedure, where first, base models were developed by testing different structural models. Second, covariate analyses were performed with these base models using forward inclusion and backward elimination procedure with significance levels of $p \leq 0{\cdot}05$ and $p \leq 0{\cdot}01$, respectively. Numerical covariates were implemented using power relationships centred to the median. For categorical covariates, factors were estimated for each level.

Pharmacokinetic modelling

First, a PK base model was developed by testing one-, two- and three-compartment models. Second, covariate analysis was carried out on the base model. Laboratory values (alanine aminotransferase (ALAT), aspartate aminotransferase (ASAT), albumin, glomerular filtration rate (GFR), C-reactive protein (CRP)) and patient characteristics (body surface area (BSA), body mass index (BMI), height, weight, sex, age, COVID-19 disease) were considered as covariates.

Time-to-event modelling

A time-to-event analysis was conducted to evaluate the endpoint ‘patients discharge’. To analyse the time to discharge the parametric survival function given in Eq. 1 was used.

$$S(t) = e^{-\int_0^t h(t)dt} \qquad \text{Eq. 1}$$

h(t) = Hazard function for discharge, S(t) = Probability of not being discharged within the time interval 0 (start of treatment period) to time t.

The modelling process was carried out in a stepwise manner. First, various baseline hazard models were evaluated, such as proportional, Gompertz, Weibull and Bateman hazard functions. Furthermore, a lag time for delayed onset of hazard was tested. Second, the drug effect of MP1032 was assessed using an Emax function, and direct links from MP1032 plasma concentration to baseline hazard were explored through an effect compartment model and transit compartments. The optimal number of transit compartments was determined. Third, covariate candidates (sex, age, weight, height, BSA, BMI, anti-viral co-medication, COVID-19-vaccination, smoke status, NIAID score at start of study, COVID-19 severity at start of study (severe/moderate), ALAT, ASAT, albumin, GFR, CRP, D-dimer) were tested on the hazard function parameter.

Biomarker modelling

For the description of the biomarkers D-dimer and eGFR over time, linear and turnover models were tested and linked to the MP1032 PK model. Then, sex, age, weight, height, BSA, BMI, anti-viral co-medication, COVID-19-vaccination, smoke status, baseline ALAT, baseline ASAT, baseline albumin, baseline GFR, and baseline D-dimer were evaluated as covariates.

Supplementary Results

Pharmacokinetics of MP1032

Overall, 24 healthy individuals, 40 psoriasis patients and four COVID-19 patients contributed to the PK dataset. Subjects were on average (median) 33·5 years old, weighed 79 kg, were 179 cm tall and 83·8% male. In total, 1138 MP1032 plasma concentrations were available.

A two-compartment model, incorporating absorption lag time of 9·84 minutes and linear elimination, was utilized to describe the concentration-time profiles of MP1032. A schematic representation of the model is depicted in Supplementary Figure 3. IIV was identified on clearance, volumes of distributions (for central and peripheral compartments; V_C and V_P), absorption rate and intercompartmental clearance. The goodness-of-fit plots show that all data points were randomly scattered around the line of identity, indicating a favourable descriptive performance (Supplementary Figure 4). All parameters were estimated precisely, with a relative standard error (RSE) below 40%. The typical V_C and V_P were estimated at 363 L and 1320 L, respectively. Deviating from the V_C of non-COVID-19 patients, a V_C of 1161·6 L was estimated for COVID-19 patients ($p<0·001$). This difference which could not be explained by the covariates tested. The average clearance of MP1032 was calculated to be 563 L/hour. For non-COVID-19 patients, half-lives for the first and terminal elimination phase were determined to 0·35 hours and 8·1 hours and for COVID-19 patients 1·09 hours and 8·4 hours, respectively.

The covariate analyses revealed body weight as a statistically significant ($p < 0·001$) covariate. V_C was increased by increasing body weight, implemented as a power model with an estimated exponent of 1·2. Exemplarily, a 90 kg person has a 35% higher V_C compared to a person weighing 70 kg. The typical clearance (CL) was 563 L/hour and increased by body weight with an estimated exponent of 1·47. Exemplarily, a 90 kg person has a 45% higher CL than a person weighing 70 kg. Individual PK profiles are depicted in Supplementary Figure 5.

Time-to-event analysis

The dataset for TTE model development contained 131 COVID-19 patients, 86 receiving MP1032 +SoC and 45 patients receiving placebo + SoC. In total, 107 patients were discharged within 28 days after start of treatment. A Bateman hazard function with two preceding transit compartments was found to best describe the "time to discharge". No immediate effect of MP1032 exposure on the baseline hazard could be identified. When the MP1032 exposure was linked to the baseline hazard using an Emax function with a 50% effective concentration (EC_{50}) of 2·4*10-5 ng/mL via five transit compartments, a significant effect ($p<0·01$) was observed. The transit compartments resulted in an estimated mean transit time (MTT) of 8·3 days. A schematic representation of the model is depicted in Supplementary Figure 3.

A covariate analysis identified that the NIAID baseline value had a significant impact ($p <0·01$) on scale parameter of the hazard function and patients age and ASAT showed significant impact ($p <0·01$) on shape parameter of hazard function. Higher NIAID baseline value resulted in better chances for discharge. Older patient age and higher ASAT values resulted in later discharge. A KM-VPC stratified by treatment shows the adequate model performance (Supplementary Figure 6).

Biomarker analysis

D-dimer

The dataset for model development contained 667 D-dimer measurements from 131 COVID-19 patients. For the description of the COVID-19 biomarker D-dimer, a turnover model was used as base model. Due to the concurrent SARS-CoV-2 infection, non-steady state conditions were assumed at baseline and the first D-dimer measurement was used to initialize the system of ordinary differential equations (ODEs). An additional time-dependent input rate was added. A schematic representation of the model is depicted in Supplementary Figure 3. A significant impact ($p<0·001$) of MP1032 exposure as predicted by the PK model was identified on the extend of the time dependent input rate with a maximum reduction of the input rate by 52·6% and an EC_{50} of 0·0784 ng/m. The individual D-dimer levels to which patients returned after 60 days were highly variable (IIV of 301·2 %CV). The covariate screening revealed no significant impact of any tested covariate on the model. All parameters were estimated precisely (RSE<31 %) and are depicted in Supplementary Table 1. Supplementary Figure 7 visualises the high inter-individual variability between 12 exemplary individual D-dimer-time profiles. Goodness-of-fit plots (Supplementary Figure 8) depict the good model performance.

eGFR

The dataset for model development contained 617 eGFR measurements from 131 COVID-19 patients. For the description of the eGFR during COVID-19, a turnover model was used as base model. Due to the concurrent

SARS-CoV-2 infection, non-steady state conditions were assumed at baseline and the first GFR measurement was used to initialize the system of ODEs. No further time-dependent processes were needed for the description of the data. A schematic representation of the model is depicted in Supplementary Figure 3. Moderate IIV (25·6 %CV) was identified on the steady-state eGFR levels. A significant impact ($p<0·001$) of the treatment group (MP1032 + SoC vs. placebo + SoC) was identified on the output rate of the turnover model with the placebo treatment group having a 74·2% lower output rate in comparison to the MP1032 treatment group, which results in a slower return of the decreased eGFR to normal levels during the infection. None of the tested covariates showed a significant impact. All model parameters were estimated precisely (RSE <33%) and are depicted in Supplementary Table 1. Supplementary Figure 9 shows exemplary individual D-dimer-time profile. Goodness-of-fit plots (Supplementary Figure 10) depict the good model performance.

Supplementary Table

Supplementary Table 1: Model parameter estimates. RSE: Relative standard error.

Parameter	Description	Unit	Estimate (RSE)
PK Model			
CL/F	Apparent clearance	L/h	563 (5%)
V_C/F	Apparent central volume of distribution	L	363 (7·30%)
k_a	Absorption rate	1/h	45·8 (8·70%)
Q/F	Apparent intercompartmental clearance	L/h	143 (12·90%)
V_P/F	Apparent peripheral volume of distribution	L	1320 (23%)
ALAG	Absorption lag time	h	0·164 (0·60%)
V_C(COVID-19)	Covariate effect of COVID-19 on V_C	-	1·2 (30·10%)
V_C(WGT)	Covariate effect of weight on V_C	-	1·47 (20·70%)
CL(WGT)	Covariate effect of weight on CL	-	3·2 (37·50%)
IIV CL	Inter-individual variability CL	%CV	24·3 (14·10%)
IIV V_C	Inter-individual variability V_C	%CV	28·7 (16·10%)
IIV k_a	Inter-individual variability k_a	%CV	172·0 (14·80%)
IIV Q	Inter-individual variability Q	%CV	59·4 (11·30%)
IIV V_P	Inter-individual variability V_P	%CV	78·7 (16.60·%)
a. error	Additive residual error	SD ng/ml	2·09 (16·10%)
p. error	Proportional residual error	%CV	48·10 (3·50%)
Time-to-event model			
k_a	Shape parameter baseline hazard	-	0·0201 (18·7%)
k_{el}	Shape parameter baseline hazard	-	0·0202 (36·8%)
F	Scale parameter baseline hazard	-	0·0216 (35·6%)
$k_{tr,Hazard}$	Hazard transit rate	1/h	0·0168 (10·2%)
EC_{50}	Concentration at half-maximum effect	µg/ml	0·024 (fix)
$k_{tr,Drug}$	Drug effect transit rate	1/h	0·03 (16·8%)
$Effect_{ASAT}$	Exponential effect of ASAT on hazard (k_{el})	-	0·589 (38·7%)
$Effect_{Age}$	Exponential effect of patients age on hazard (k_{el})	-	2·21 (25·3%)
$Effect_{NIAID}$	Exponential effect of NIAID score on hazard (F)	-	2·99 (34·1%)
D-dimer model			
BL_{DD}	Steady state D-dimer levels	ng/ml	48 (10·6%)
$k_{deg,DD}$	Degradation rate	1/day	0·113 (18·7%)
MAX_{DD}	Maximum of the time-dependent input	ng/(ml*day)	36·2 (30·4%)
Slope	Slope of time-dependent input	1/day	0·0283 (15·8%)
$Emax_{DD}$	Maximum effect of MP1032	-	-0·526 (12·1%)
$EC50_{DD}$	Half-maximal effective MP1032 concentration	ng/ml	0·0784 (27·7%)
IIV MAX_{DD}	Inter-individual variability MAX_{DD}	%CV	301·2 (30·8%)
p. error	Proportional residual error	%CV	44·7 (5·1%)
eGFR model			
BL_{GFR}	Steady state eGFR levels	ml/min	82·3 (10·9%)
$k_{deg,GFR}$	Degradation rate	1/day	0·217 (32·2%)
$BL_{GFR(BL)}$	Covariate effect of observed GFR baseline on BL_{GFR}	-	0·839 (6·9%)
$k_{deg(Tret)}$	Effect of treatment with MP1032 on k_{deg}	-	-0·742 (11·9%)
IIV BL_{GFR}	Inter-individual variability BL_{GFR}	%CV	25·6 (0·03%)
p. error	Proportional residual error	%CV	12 (6%)

Supplementary Figures

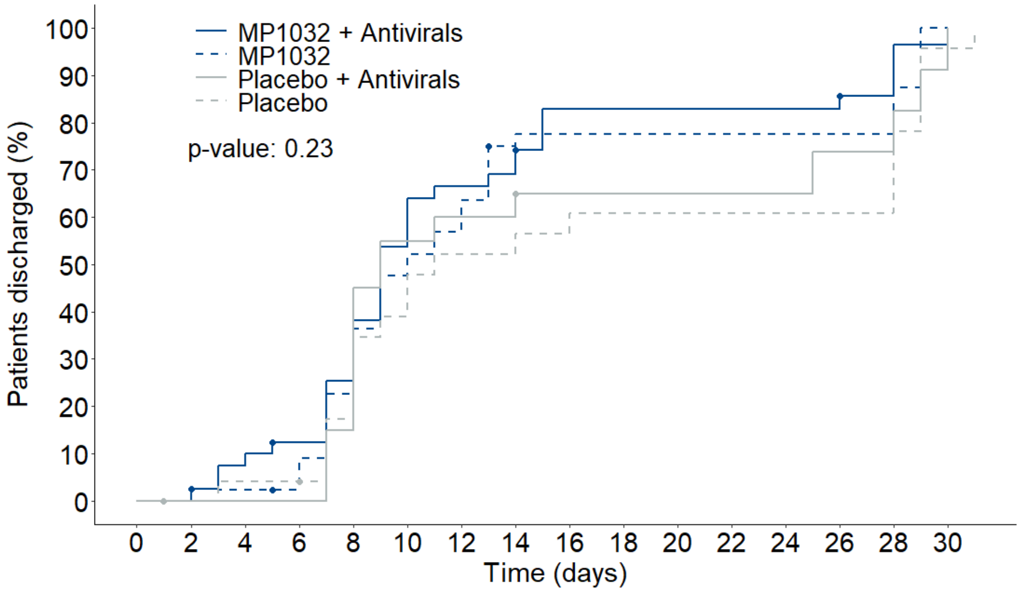

Number at Risk

MP1032 + Antivirals	41	41	37	34	29	18	13	12	6	6	6	6	6	6	4	1
MP1032	45	45	44	43	34	23	19	10	9	9	9	9	9	9	9	0
Placebo + Antivirals	20	20	20	20	17	9	8	8	4	4	4	4	4	3	3	1
Placebo	25	24	23	23	19	14	11	11	10	9	9	9	9	9	9	1

Supplementary Figure 1: Time to discharge of patients with and without antiviral SoC treatment. Kaplan-Meier estimates for time to discharge of patients after treatment start with MP1032 + SoC (blue) or placebo + SoC (grey) stratified by the presence (solid lines) or absence (dashed lines) of additional anti-viral therapy. Dots represent censored data.

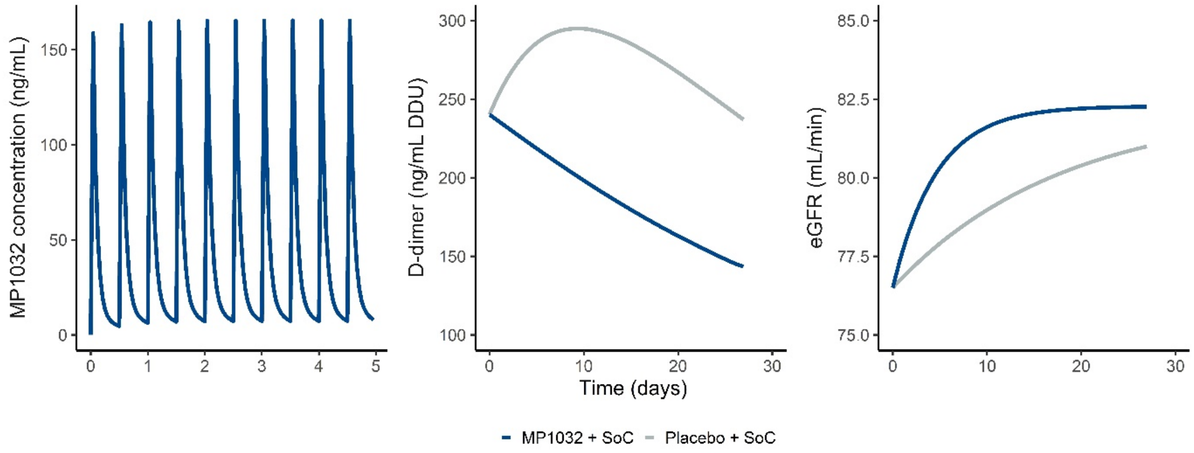

Supplementary Figure 2: Simulation results of the PK model for MP1032 and the models of biomarkers D-dimer and eGFR in COVID-19 patients treated with MP1032 + SoC vs. placebo + SoC.

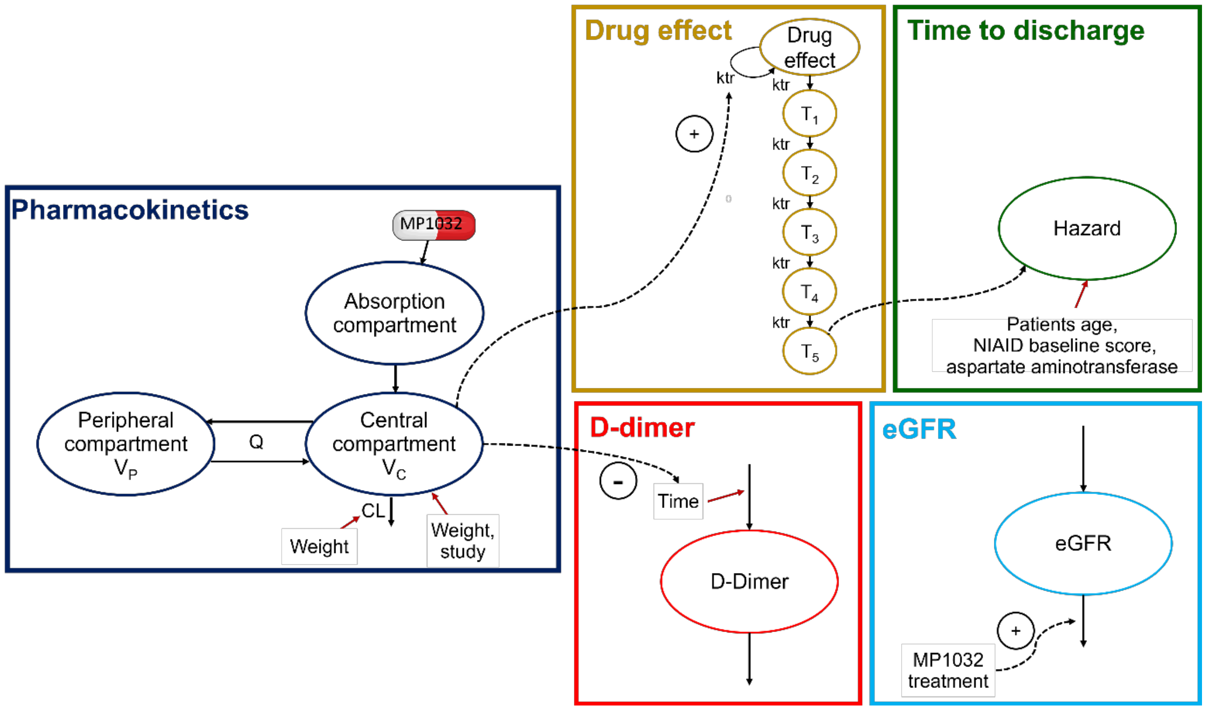

Supplementary Figure 3: Schematic representation of all models. V_P: Volume of distribution for peripheral compartments; V_C: Volume of distribution for central compartments; CL: Clearance; k_{tr}: Drug effect transit rate, Q: Intercompartmental clearance, k_a: Absorption rate, $k_{deg,DD}$: Degradation rate (D-dimer), $k_{deg,GFR}$: Degradation rate (eGFR)

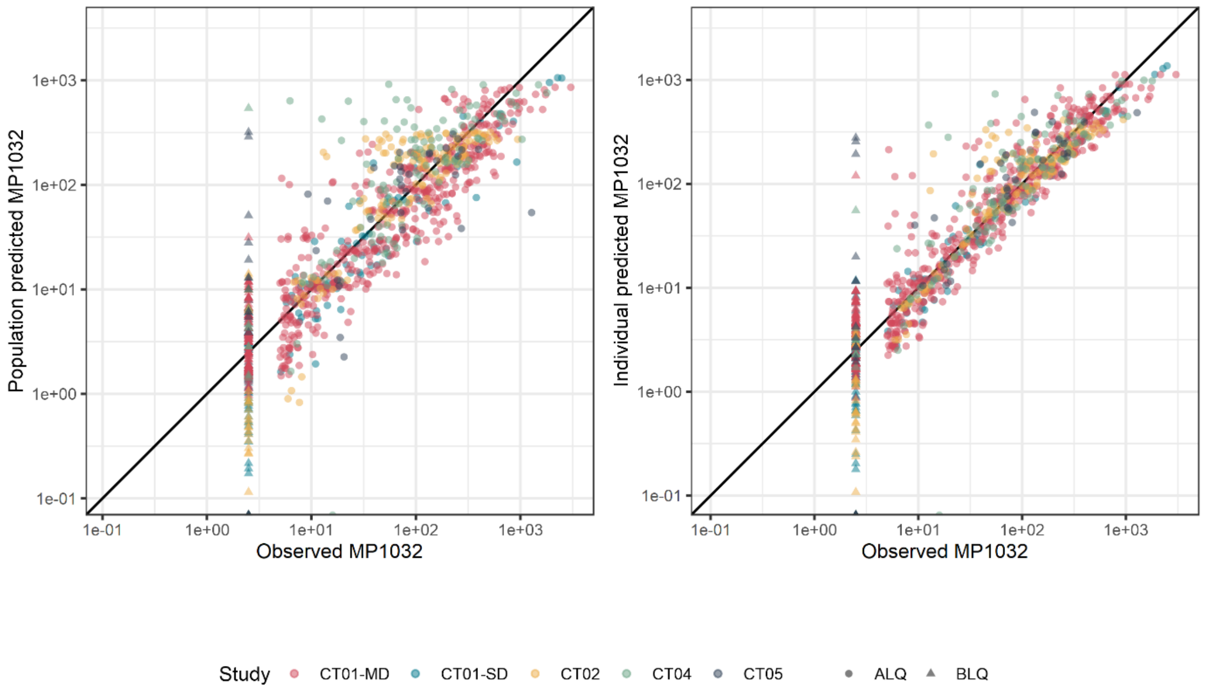

Supplementary Figure 4: Goodness-of-fit plots of MP1032 PK model. ALQ: above lower limit of quantification, BLQ: below lower limit of quantification.

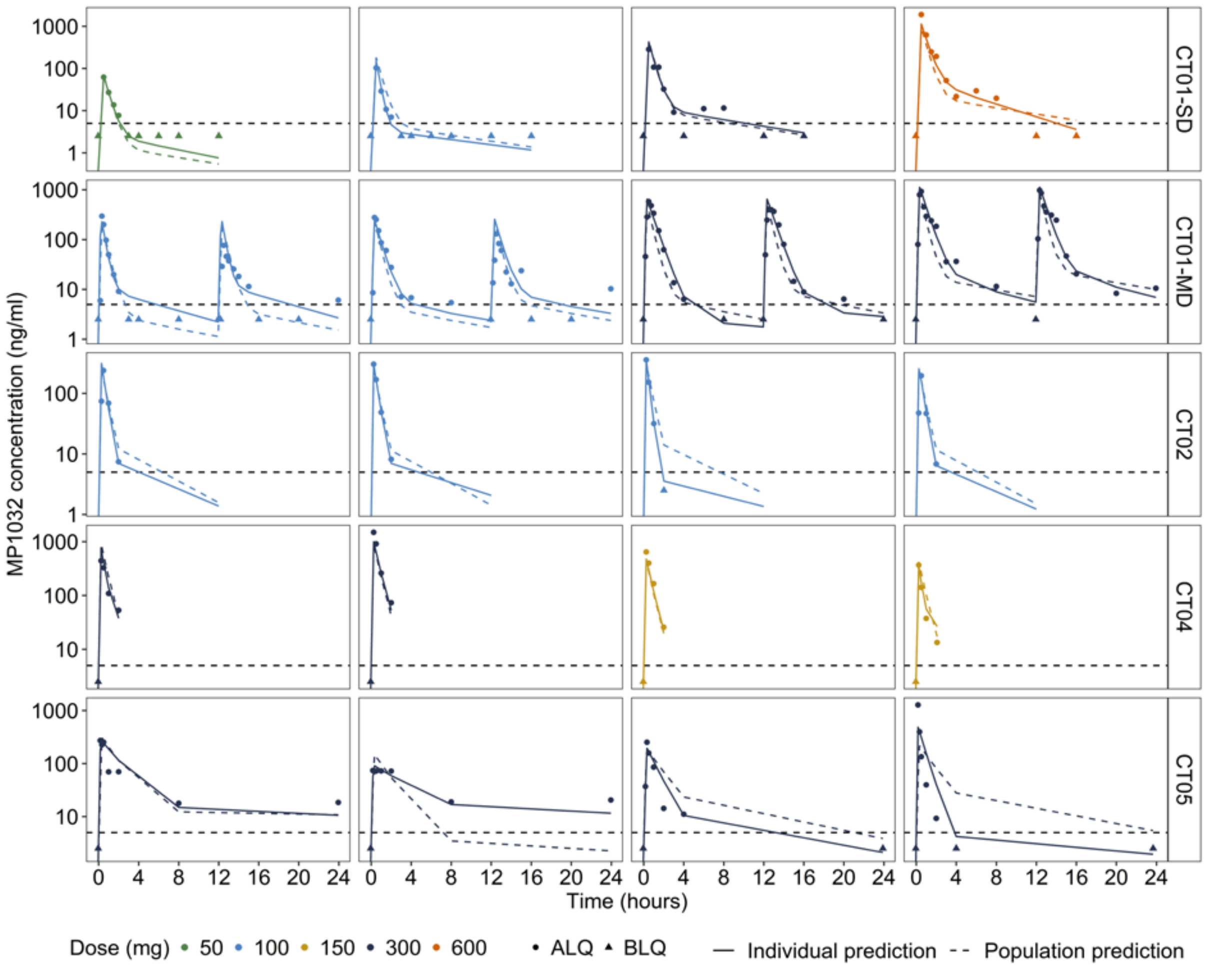

Supplementary Figure 5: Randomly drawn individual MP1032 profiles. Points represent observations. Lines represent model predictions. The dashed line indicates the lower limit of quantification.

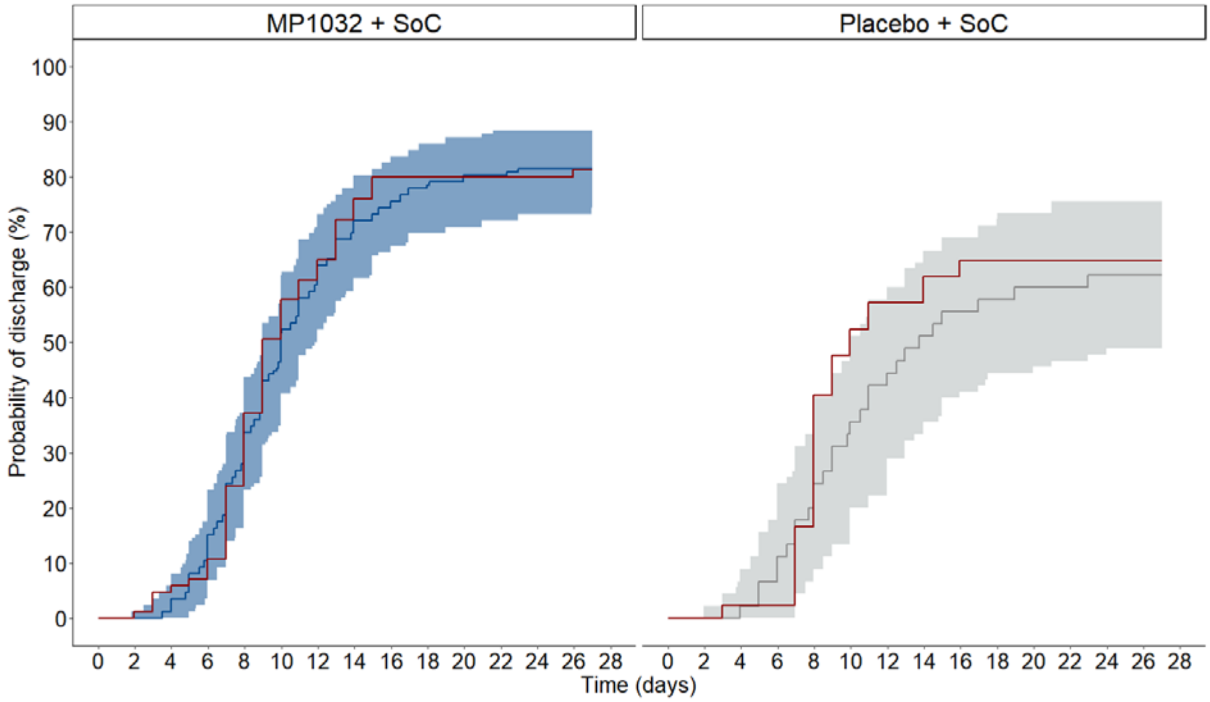

Supplementary Figure 6: Kaplan-Meier plots of the probability of discharge over time. Left panel: MP1032 + Soc treatment group. Right panel: Placebo + SoC treatment group. Red solid lines represent the observed cumulative incidence of discharge over time. The blue and the grey solid lines represent the simulation median for hospital discharge based on the final model for MP1032 and placebo, respectively. Coloured shaded area represents the 95% confidence interval calculated from simulations of 500 replicates.

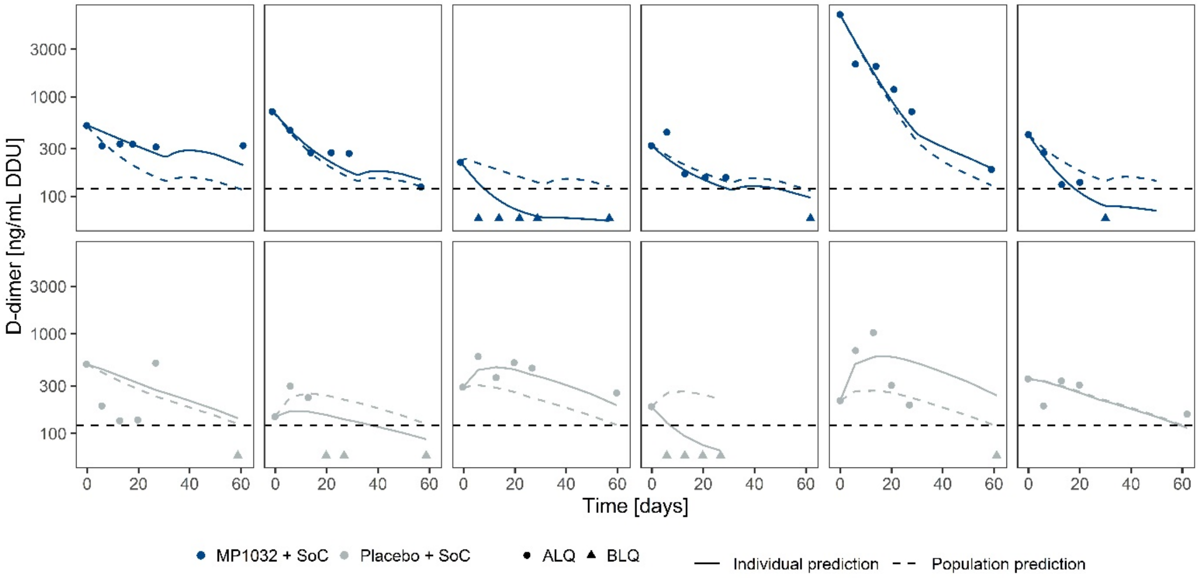

Supplementary Figure 7: Randomly drawn individual D-dimer-time profiles. Points represent observations. Lines represent model predictions. The dashed line indicates the lower limit of quantification. ALQ: above lower limit of quantification, BLQ: below lower limit of quantification.

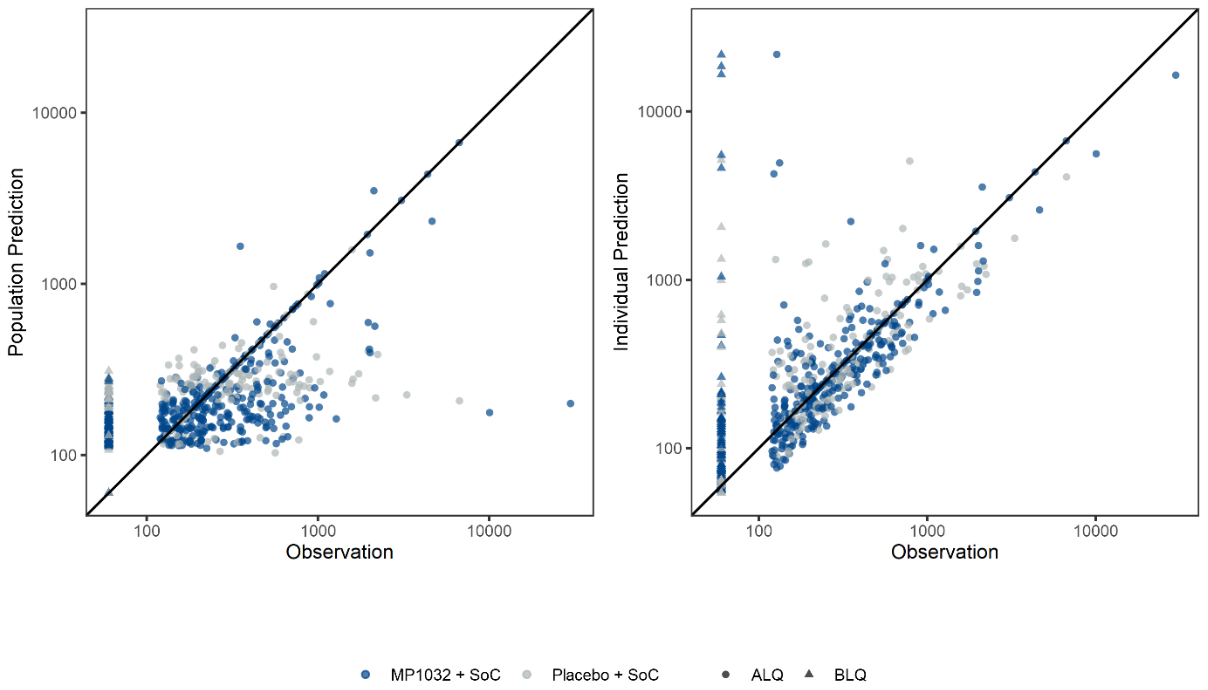

Supplementary Figure 8: Goodness-of-fit plots of D-dimer model. ALQ: above lower limit of quantification, BLQ: below lower limit of quantification

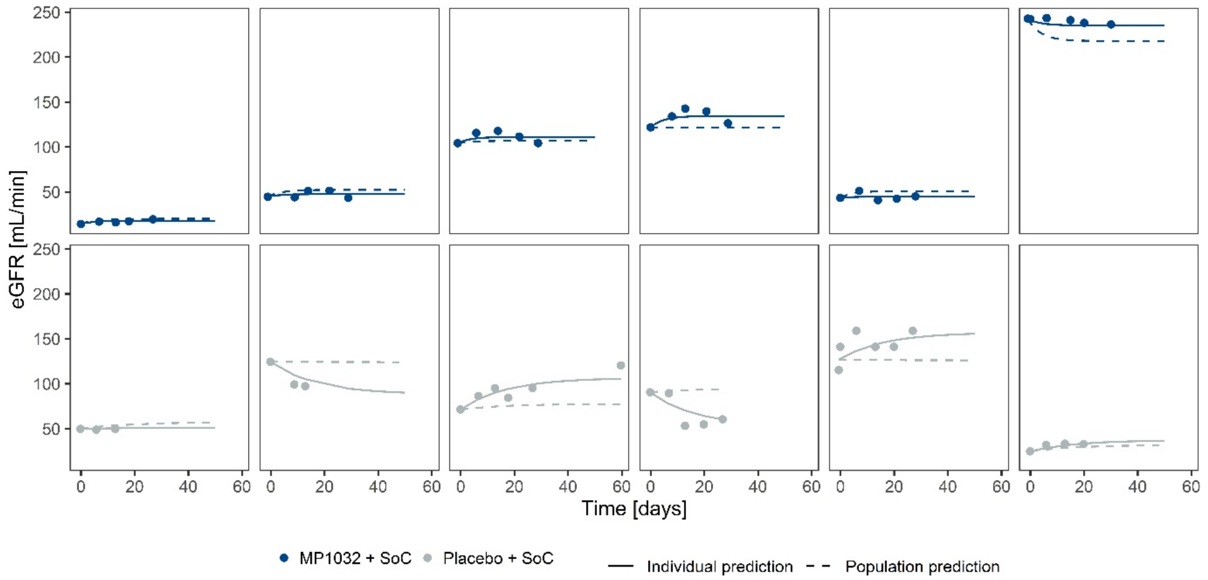

Supplementary Figure 9: Randomly drawn individual eGFR-time profiles. Points represent observations. Lines represent model predictions.

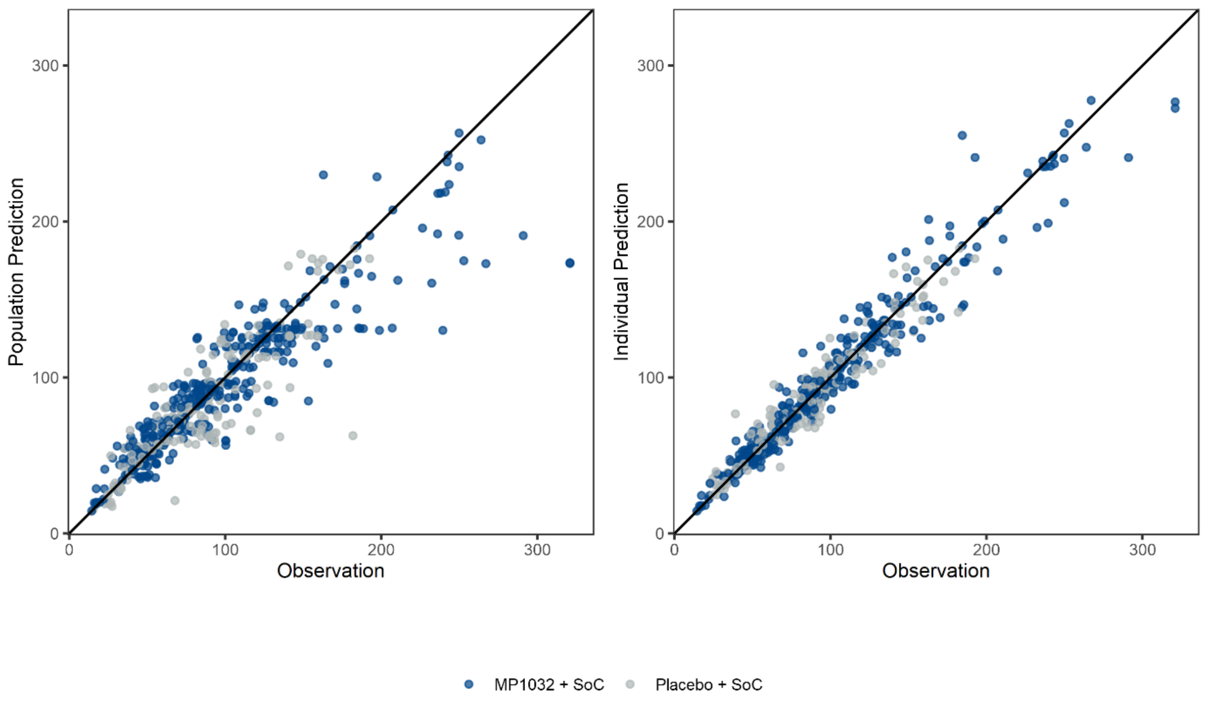

Supplementary Figure 10: Goodness-of-fit plots of eGFR model.

Supplementary References

[1] Bauer RJ. NONMEM Tutorial Part II: Estimation Methods and Advanced Examples. *CPT Pharmacometrics Syst Pharmacol*, 2019. **8**(8): p. 538-556.

[2] Karlsson MO, Savic RM. Diagnosing model diagnostics. *Clin Pharmacol Ther*, 2007. **82**(1): p. 17-20.

[3] Upton RN, Mould DR. Basic RM. concepts in population modeling, simulation, and model-based drug development: part 3-introduction to pharmacodynamic modeling methods. *CPT Pharmacometrics Syst Pharmacol*, 2014. **3**(1): p. e88.
