# Supplementary material for: Efficacy and safety of MP1032 plus standard-of-care compared to standard-of-care in hospitalised patients with COVID-19: a multicentre, randomised double-blind, placebo-controlled phase 2a trial
Source: Lancet Reg Health Eur. 2023 Dec 6;37:100810. doi: 10.1016/j.lanepe.2023.100810 (PMC10704330; doi:10.1016/j.lanepe.2023.100810)
Supplement: Protocol [file mmc2.pdf]

1 CLINICAL STUDY PROTOCOL

Protocol Title: A RANDOMIZED, DOUBLE-BLIND, PLACEBO-CONTROLLED, MULTICENTER, PROOF-OF-CONCEPT, PHASE IIA STUDY OF MP1032 PLUS STANDARD OF CARE VS STANDARD OF CARE IN THE TREATMENT OF HOSPITALIZED PATIENTS WITH MODERATE TO SEVERE COVID-19

Protocol Number: MP1032-CT05

Short Title: MP1032 Treatment in Patients with Moderate to Severe COVID-19

**Investigational New Drug Number:**	153604
**Name of Investigational Product:**	MP1032 hard gelatin capsules 50 mg
**Phase of Development:**	IIa
**Indication:**	Treatment of symptomatic patients with moderate to severe coronavirus disease 2019 (COVID-19) disease
**EudraCT Number:**	2021-000344-21
**Sponsor's EU Representative:**	Petra Schulz<br>Senior Project Manager<br>MetrioPharm Deutschland GmbH<br>Am Borsigturm 100<br>13507 Berlin<br>Germany<br>Tel: +49 30 338439536<br>Email: p.schulz@metriopharm.com
**Sponsor:**	MetrioPharm AG<br>Bleicherweg 10<br>8002 Zürich<br>Switzerland
**Protocol Version:**	2.0
**Protocol Date:**	31-Jan-2022

-CONFIDENTIAL-

This document and its contents are the property of and confidential to MetrioPharm AG. Any unauthorized copying or use of this document is prohibited.

PROTOCOL APPROVAL SIGNATURES

**Protocol Title:**	A RANDOMIZED, DOUBLE-BLIND, PLACEBO-CONTROLLED, MULTICENTER, PROOF-OF-CONCEPT, PHASE IIA STUDY OF MP1032 PLUS STANDARD OF CARE VS STANDARD OF CARE IN THE TREATMENT OF HOSPITALIZED PATIENTS WITH MODERATE TO SEVERE COVID-19
**Protocol Number:**	MP1032-CT05

This study will be conducted in compliance with the clinical study protocol (and amendments), International Council for Harmonisation guidelines for current Good Clinical Practice (GCP), and applicable regulatory requirements.

Sponsor Signatory

[redacted]

Signature

Date (DD-Mmm-YYYY)

Medical Signatory

[redacted]

Signature

Date (DD-Mmm-YYYY)

Biostatistician Signatory

[redacted]

Signature

Date (DD-Mmm-YYYY)

INVESTIGATOR SIGNATURE PAGE

Protocol Title: A RANDOMIZED, DOUBLE-BLIND, PLACEBO-CONTROLLED, MULTICENTER, PROOF-OF-CONCEPT, PHASE IIA STUDY OF MP1032 PLUS STANDARD OF CARE VS STANDARD OF CARE IN THE TREATMENT OF HOSPITALIZED PATIENTS WITH MODERATE TO SEVERE COVID-19

Protocol Number: MP1032-CT05

Confidentiality and Current Good Clinical Practice (GCP)/E6(R2)/Compliance Statement

- I, the undersigned, have reviewed this protocol (and any amendments), including appendices, and I will conduct the study as described in compliance with this protocol (and any amendments), GCP, and relevant International Council for Harmonisation guidelines.
- I am thoroughly familiar with the appropriate use of the study drug, as described in this protocol and any other information provided MetrioPharm AG including, but not limited to, the current investigator's brochure.
- Once the protocol has been approved by the institutional review board (IRB)/independent ethics committee (IEC), I will not modify this protocol without obtaining prior approval of MetrioPharm AG and of the IRB/IEC. I will submit the protocol amendments and/or any informed consent form (ICF) modifications to MetrioPharm AG and the IRB/IEC, and approval will be obtained before any amendments are implemented.
- I ensure that all persons or parties assisting me with the study are adequately qualified and informed about the MetrioPharm AG study drug and of their delegated study-related duties and functions as described in the protocol.
- I ensure that source documents and study records that include all pertinent observations on each of the site's patients will be attributable, legible, contemporaneous, original, accurate, and complete.
- I understand that all information obtained during the conduct of the study with regard to the patients' state of health will be regarded as confidential. No patients' names will be disclosed. All patients will be identified by assigned numbers on all case report forms, laboratory samples, or source documents forwarded to the sponsor. Clinical information may be reviewed by the sponsor or its agents or regulatory agencies. Agreement must be obtained from the patient before disclosure of patient information to a third party.
- Information developed in this clinical study may be disclosed by MetrioPharm AG to other clinical investigators, regulatory agencies, or other health authority or government agencies as required.

____
Printed Name

____
Investigator Signature

____
Title

____
Date (DD-Mmm-YYYY)

____
Institution

2 SYNOPSIS

**Title of Study:**	A RANDOMIZED, DOUBLE-BLIND, PLACEBO-CONTROLLED, MULTICENTER, PROOF-OF-CONCEPT, PHASE IIA STUDY OF MP1032 PLUS STANDARD OF CARE VS STANDARD OF CARE IN THE TREATMENT OF HOSPITALIZED PATIENTS WITH MODERATE TO SEVERE COVID-19
**Protocol Number:**	MP1032-CT05
**Investigators/Study Sites:**	Approximately 40 sites worldwide will participate in this study.
**Phase of Development:**	Phase IIa
**Study Objectives:**	The primary objective of this study is:<br>• To measure the effect of MP1032 plus standard of care (SoC) versus placebo plus SoC on Day 14 on disease progression in patients with moderate to severe coronavirus disease 2019 (COVID-19)<br><br>The secondary objectives of this study are:<br>• To measure the effect of MP1032 plus SoC versus placebo plus SoC on Day 28 on disease progression in patients with moderate to severe COVID-19<br>• To measure the effect of MP1032 plus SoC versus placebo plus SoC on disease resolution on Day 14 and Day 28<br>• To measure the effect of MP1032 plus SoC versus placebo plus SoC on the mortality rate and other specific COVID-19 related characteristics<br>• To assess the safety of MP1032 (eg, adverse events [AEs] and laboratory abnormalities)<br>• To assess the pharmacokinetics (PK) of MP1032 on Day 1 (single dose) and Day 7 (steady state) in a PK subset of patients<br><br>The exploratory objectives of this study are:<br>• To measure the effect of MP1032 plus SoC versus placebo plus SoC on some additional COVID-19 related characteristics<br>• To evaluate the health-related quality of life (HRQoL) of patients treated MP1032 plus SoC compared with placebo plus SoC<br>• To evaluate biomarker levels
**Study Endpoints:**	The primary efficacy endpoint of this study is:<br>• Proportion of patients with disease progression on Day 14. Disease progression is defined as the proportion of patients who are not alive or who have respiratory failure. Respiratory failure is defined as patients who have a score of 2, 3, or 4 on the National Institute of Allergy and Infectious Diseases (NIAID) 8-point ordinal scale (see below).<br><br>The key secondary efficacy endpoints of this study are:

	• Proportion of patients with disease progression on Day 28. Disease progression is defined as the proportion of patients who are not alive or who have respiratory failure. Respiratory failure is defined as patients who have a score of 2, 3, or 4 on the NIAID 8-point ordinal scale (see below).<br>• Proportion of patients with disease resolution on Day 28. Disease resolution is defined as patients who are alive and have a score of 6, 7, or 8 on the NIAID 8-point ordinal scale.<br>• All-cause mortality rate at Day 28<br>• Change of clinical status related to COVID-19 on Day 28 compared with baseline according to the following NIAID 8-point ordinal scale:<br>1. Death<br>2. Hospitalized, on invasive ventilation (mechanical ventilator and/or extracorporeal membrane oxygenation [ECMO])<br>3. Hospitalized, on non-invasive ventilation or high-flow oxygen devices<br>4. Hospitalized, requiring supplemental oxygen<br>5. Hospitalized, not requiring supplemental oxygen, but requiring ongoing medical care (COVID-19 related or otherwise)<br>6. Hospitalized, not requiring supplemental oxygen and no longer requires ongoing medical care (used if hospitalization was extended for infection-control reasons)<br>7. Not hospitalized, limitation on activities, and/or requiring home oxygen<br>8. Not hospitalized, no limitations on activities<br>Please note: Patients requiring oxygen before COVID-19 and returning to baseline oxygen use will be considered improved (ie, not requiring supplemental oxygen). Patients with a limitation on activities before COVID-19 and returning to baseline activity will be considered improved. In case of death before Day 14 or Day 28, the patient will be considered with the NIAID score for death (score of 1) on the date of death in the analysis.<br><br>The other secondary endpoints of this study are:<br>• Proportion of patients with disease resolution on Day 14. Disease resolution is defined as patients who are alive and have a score of 6, 7, or 8 on the NIAID 8-point ordinal scale.<br>• All-cause mortality rate at Day 14 and Day 60<br>• Change of clinical status related to COVID-19 on Day 14 compared with baseline according to the NIAID 8-point ordinal scale as listed above.<br>• Proportion of patients requiring invasive ventilation (mechanical ventilator and/or ECMO), or who are not alive on Day 14 or Day 28<br>• Proportion of patients in each category of the NIAID 8-point ordinal scale<br>• Time to (first) improvement of at least 1 category on the NIAID 8-point ordinal scale (until Day 28). Patients who did not improve at least 1 category on the NIAID scale or die before Day 28 will be censored at Day 28.<br>• The odds ratio between MP1032 and SoC and placebo and SoC for the number of patients with clinical status improvement from baseline on the NIAID 8-point ordinal scale (ie, an improvement of at least 1 category) at Day 14 and Day 28

	• Total duration of hospitalization on Day 28 and Day 60 (from baseline to discharge; with death censored on the last day of the observed period – at Day 28 or Day 60, respectively)<br>• Proportion of patients alive and testing negative for COVID-19 on Day 14, Day 28, and Day 60<br>• Safety and tolerability assessed by:<br>○ Cumulative incidence of treatment-emergent AEs (summarized by seriousness, severity, relationship to the study medication, outcome, and duration)<br>○ Vital sign parameters<br>○ Clinical laboratory parameters<br>○ Physical examination findings<br>• MP1032 plasma concentrations and PK parameters (if possible) including maximum observed plasma concentration, area under the concentration-time curve, elimination parameters, apparent body clearance, apparent volume of distribution, trough concentration, average observed plasma concentration at steady state, and other relevant PK parameters assessed via MP1032 plasma exposure on Day 1 and Day 7 in a PK subset of patients<br><br>Exploratory endpoints of this study are:<br>• Change in saturation of oxygen (SpO_2)/fraction of inspired oxygen (FiO_2) ratio (for patients alive) on Day 14 or Day 28 compared with baseline<br>• Total number of days in the intensive care unit (ICU)<br>• Duration of invasive mechanical ventilation<br>• Duration of ECMO<br>• Time to recovery from COVID-19 symptoms (stuffy or runny nose, sore throat, red or irritated eyes, shortness of breath, cough, low energy or tiredness, muscle or body aches, headache, chills or shivering, feeling hot or feverish, nausea, and number of times of vomit, times of diarrhea, sense of smell, sense of taste in the last 24 hours) at Day 14, Day 28, and Day 60<br>• Change from discharge in the EuroQol (EQ) index value and EQ visual analog scale (VAS) based on the EuroQol-5D-5L (EQ-5D-5L) questionnaire at Day 60<br>• Change from baseline in biomarker levels potentially including, but not limited to, cytokines (eg, C-reactive protein, interleukin [IL]-1β, IL-6, tumor necrosis factor-α, and interferon-γ), and other coagulation/inflammatory biomarkers (eg, D-dimer and ferritin)
**Study Design:**	This is a Phase IIa, randomized, double-blind, placebo-controlled, multicenter, proof-of-concept study designed to assess the efficacy and safety of 300 mg MP1032 twice daily (BID) plus SoC versus placebo plus SoC in hospitalized adults with moderate to severe COVID-19.<br><br>Approximately 140 patients will be screened to randomize approximately 120 patients in 2:1 ratio as follows:<br>• Arm A (300 mg MP1032 BID plus SoC): approximately 80 patients<br>• Arm B (placebo BID plus SoC): approximately 40 patients

	The stratification factor for randomization will include baseline COVID-19 severity (moderate versus severe) and age-class (aged ≤65 years versus >65 years). COVID-19 severity will be determined using the following criteria:<br>• Moderate COVID-19:<br>o Positive severe acute respiratory syndrome coronavirus type 2 (SARS-CoV-2) testing by standard reverse transcription-polymerase chain reaction (RT-PCR) assay or equivalent test<br>o Symptoms of moderate illness with COVID-19, which could include any symptom of mild illness or shortness of breath with exertion<br>o Clinical signs suggestive of moderate illness with COVID-19, such as respiratory rate ≥20 breaths per minute, SpO_2 >93% (on room air at sea level, if possible), heart rate ≥90 beats per minute<br>o No clinical signs indicative of severe or critical COVID-19<br>• Severe COVID-19:<br>o Positive SARS-CoV-2 testing by standard RT-PCR assay or equivalent test<br>o Symptoms suggestive of severe systemic illness with COVID-19, which could include any symptom of moderate illness or shortness of breath at rest, or respiratory distress<br>o Clinical signs suggestive of severe systemic illness with COVID-19, such as respiratory rate ≥30 breaths per minute, heart rate ≥125 beats per minute, SpO_2 ≤93% (on room air at sea level, if possible), partial pressure of oxygen/FiO_2 <300, or diagnosed with acute respiratory distress syndrome (according to the Berlin definition)<br>o No criteria met for critical COVID-19<br><br>To standardize the assessment of COVID-19 severity, respiratory rate, SpO_2, and heart rate will be measured when the patient is on room air at sea level (ie, no supplemental oxygen) and at rest for at least 5 minutes. If possible, the site should collect the information from each patient at the same time each day (±1 hour).<br><br>Please note: Patients who are receiving oxygen therapy at baseline for a chronic condition (eg, emphysema, chronic obstructive pulmonary disease [COPD], pulmonary arterial hypertension [PAH], idiopathic pulmonary fibrosis [IPF], etc) must be considered as having severe COVID-19 (unless the patient meets the definition for critical COVID-19). For questions related to COVID-19 severity, the investigator should contact the medical monitor.<br><br>Each site will treat all patients with SoC for the duration of the study. The selected SoC will be used in accordance with the hospital's SoC procedures and may include drugs under an emergency use authorization.<br><br>This study consists of 3 periods: (1) screening, (2) treatment, and (3) follow-up. During the screening period, each potential patient (or patient's legally acceptable representative) will provide informed consent before starting any study-specific procedures. The randomization of patients to treatment groups will be performed

	centrally by an interactive web-response system (IWRS) using a randomization scheme that will be developed by an unblinded, independent statistician. During the treatment period, randomized patients will be provided their assigned treatment and assessments according to the protocol. All patients will be treated for 28 days unless the study drug is discontinued for safety reasons. Patients who progress to the use of an invasive ventilation (mechanical ventilator and/or ECMO) or who can no longer swallow the study drug (>2 days [ie, 2-day interruption is acceptable]) must discontinue the study drug; but whenever possible, the study assessments will be performed on Day 14, Day 21, Day 28, and Day 60. If the patient is discharged home before Day 28, the study team will provide the patient with the remainder of the assigned blinded study drug kit(s) to take home along with instructions on how to continue treatment at home on the day-of-discharge; for these patients, treatment compliance will be conducted via a diary. If the patient does not feel comfortable to complete the diary, the site will alternatively provide the patient with the option to be called, twice a day, to confirm that the study drug is taken as instructed. The treatment period will end with the Day 28 (End of Treatment Visit) assessments. The follow-up period will consist of Day 60 (Follow-up Visit) assessments.
**Selection of Patients:**	**Inclusion Criteria:**<br>Individuals must meet all of the following criteria to be included in the study:<br>1. The patient must be willing and able to give informed consent to participate in the study and to adhere to the procedures stated in the protocol or, for adults incapable of consenting due to their medical condition (eg, too weak or debilitated, severe shortness of breath) or due to literacy issues, the patient's legally authorized representative must be willing and able to give informed consent on behalf of the patient to participate in the study as permitted by local regulatory authorities, institutional review boards (IRBs)/independent ethics committees (IECs), or local laws.<br>2. The patient is male or female adult aged ≥18 years (as per local laws) at the time of giving informed consent.<br>3. The patient is admitted to a hospital and has a positive SARS-CoV-2 test by standard RT-PCR assay or equivalent test. Please note: If the patient has a previous confirmation of SARS-CoV-2 (within 7 days of Day 1), the SARS-CoV-2 test at screening is not required.<br>4. The patient has the presence of any symptom(s) suggestive of moderate or severe systemic illness with COVID-19 on Day 1, such as presence of fever (≥38.0°C [≥100.4°F] by any route), loss of smell or taste, cough, sore throat, malaise, headache, muscle pain, gastrointestinal symptoms, shortness of breath upon exertion and/or at rest, or respiratory distress.<br>5. The patient has the presence of moderate to severe clinical signs indicative of moderate or severe illness with COVID-19 on Day 1:<br>a) Moderate:<br>i. Clinical signs suggestive of moderate illness with COVID-19, such as respiratory rate ≥20 breaths per minute, SpO_2 >93% (on room air at sea level, if possible), heart rate ≥90 beats per minute

	ii. No clinical signs indicative of severe or critical COVID-19<br>b) Severe:<br>i. Clinical signs suggestive of severe systemic illness with COVID-19, such as respiratory rate ≥30 breaths per minute, heart rate ≥125 beats per minute, SpO_2 ≤93% (on room air at sea level, if possible), partial pressure of oxygen/FiO_2 <300, or diagnosed with acute respiratory distress syndrome (according to the Berlin definition)<br>ii. No criteria met for critical COVID-19<br><br>Please note: Patients who are receiving oxygen therapy at baseline for a chronic condition (eg, emphysema, COPD, PAH, IPF, etc) must be considered as having severe COVID-19 (unless the patient meets the definition for critical COVID-19). For questions related to COVID-19 severity, the investigator should contact the medical monitor.<br><br>6. The patient does not require hemodialysis (chronic) or any renal replacement therapies at screening or Day 1.<br>7. The patient is able to swallow the study drug (hard gelatin capsules).<br>8. The patient agrees to minimize strong sun exposure (sunbathing) and strong ultraviolet exposure during the course of the study. Additionally, during the study, patients must agree to use sunscreen when spending an extended period outdoors.<br>9. Men whose sexual partners are women of childbearing potential (WOCBP) must agree to comply with 1 of the following contraception requirements from the time of first dose of study medication (Day 1) until at least 30 days after the last dose of study medication:<br>a) Vasectomy with documentation of azoospermia.<br>b) Sexual abstinence (defined as refraining from heterosexual intercourse from the time of first dose of study medication until at least 30 days after the last dose of study medication)<br>c) Male condom plus partner use of 1 of the contraceptive options below: contraceptive subdermal implant; intrauterine device or intrauterine system; oral contraceptive, either combined or progestogen alone; injectable progestogen; contraceptive vaginal ring; percutaneous contraceptive patches.<br>The above is an all-inclusive list of those methods that meet the following definition of highly effective: having a failure rate of <1% per year when used consistently and correctly and, when applicable, in accordance with the product label. For non-product methods (eg, male sterility), the investigator will determine what is consistent and correct use. The investigator is responsible for ensuring that patients understand how to properly use these methods of contraception.<br>10. WOCBP must agree to comply with 1 of the following contraception requirements from the time of first dose of study medication (Day 1) until at least 30 days after the last dose of study medication:

	a) Sexual abstinence (defined as refraining from heterosexual intercourse from the time of first dose of study medication until at least 30 days after the last dose of study medication)<br>b) Use of 1 of the contraceptive options below plus use of a condom by male partner: contraceptive subdermal implant; intrauterine device or intrauterine system; oral contraceptive, either combined or progestogen alone; injectable progestogen; contraceptive vaginal ring; percutaneous contraceptive patches.<br>c) Vasectomy of male partner with documentation of azoospermia.<br>The above is an all-inclusive list of those methods that meet the following definition of highly effective: having a failure rate of <1% per year when used consistently and correctly and, when applicable, in accordance with the product label. The investigator is responsible for ensuring that patients understand how to properly use these methods of contraception. Women of non-reproductive potential are defined as:<br>a) Premenopausal females with 1 of the following: documented tubal ligation; documented hysteroscopic tubal occlusion procedure with follow-up confirmation of bilateral tubal occlusion; hysterectomy; documented bilateral oophorectomy.<br>b) Postmenopausal defined as 12 months of spontaneous amenorrhea [in questionable cases a blood sample with simultaneous follicle stimulating hormone and estradiol levels consistent with menopause (refer to laboratory reference ranges for confirmatory levels)]. Women on hormone replacement therapy (HRT) and whose menopausal status is in doubt will be required to use 1 of the highly effective contraception methods listed above if they wish to continue their HRT during the study. Otherwise, they must discontinue HRT to allow confirmation of postmenopausal status before randomization.<br>**Exclusion Criteria:**<br>Individuals meeting any of the following criteria are ineligible to participate in this study:<br>1. The patient, in the opinion of the investigator, is not likely to survive for ≥48 hours beyond Day 1.<br>2. The patient has a diagnosis of asymptomatic COVID-19, mild COVID-19, or critical COVID-19 on Day 1.<br>a) Asymptomatic COVID-19 is defined as a patient with a positive SARS-CoV-2 test by standard RT-PCR assay or equivalent test but not experiencing symptoms.<br>b) Mild COVID-19 is defined as a patient with a positive SARS-CoV-2 test by standard RT-PCR assay or equivalent test and experiencing symptoms of mild illness but no clinical signs indicative of moderate, severe, or critical COVID-19.<br>c) Critical COVID-19 is defined as a patient with a positive SARS-CoV-2 test by standard RT-PCR assay or equivalent test and experiencing at least 1 of the following: shock defined by systolic blood pressure <90 mm Hg or diastolic blood pressure <60 mm Hg, or requiring vasopressors; respiratory failure

requiring endotracheal intubation and invasive mechanical ventilation, oxygen delivered by high-flow nasal cannula (heated, humidified, oxygen delivered via reinforced nasal cannula at flow rates >20 L/min with fraction of delivered oxygen ≥0.5), non-invasive positive pressure ventilation, ECMO, or clinical diagnosis of respiratory failure (ie, clinical need for 1 of the preceding therapies, but preceding therapies not able to be administered in setting of resource limitation), and/or multi-organ dysfunction/failure.

Please note: Patients who are receiving oxygen therapy at baseline for a chronic condition (eg, emphysema, COPD, PAH, IPF, etc) must be considered as having severe COVID-19 (unless the patient meets the definition for critical COVID-19). For questions related to COVID-19 severity, the investigator should contact the medical monitor.

3. The patient has a Child Pugh score ≥ C.
4. The patient has a documented medical history of infection with hepatitis A, B, or C at screening or Day 1.
5. The patient has a documented medical history of infection with human immunodeficiency virus <u>and</u> has a detectable viral load and CD4 count <500 cells/µL.
6. The patient has a documented active infection with tuberculosis at screening or Day 1.
7. The patient has clinically significant electrocardiogram abnormalities at screening.
8. A female patient who is pregnant, planning to become pregnant during the study, breastfeeding, or has a positive pregnancy test at screening (by serum) and before dosing on Day 1 (by urine) as determined by human chorionic gonadotrophin tests.
9. The patient is planning to donate or bank ova or sperm from Day 1 until 30 days after the last dose of study drug.
10. The patient has a known history of drug or alcohol abuse within 6 months of study start that would interfere with the patient's participation in the study.
11. The patient has a history of sensitivity to any of the study medications, components thereof (eg, mannitol or gelatin), or a history of drug or other allergy that, in the opinion of the investigator or medical monitor, would contraindicate their participation.
12. The patient has participated in and/or plans to participate in another clinical study using an investigational product within the following period before the first dosing day in the current study: 30 days, 5 half-lives, or twice the duration of the biological effect of the investigational product (whichever is longer).
13. The patient will be transferred to another hospital that is not a study site within 72 hours. Please note: If the investigator has admitting privileges to the transfer hospital, the patient may be considered for randomization.
14. The patient is employed by MetrioPharm, the contract research organization or clinical site involved in the clinical study.

	15. The investigator makes a decision that study involvement is not in patient's best interest, or the patient has any condition or critical illness, in the opinion of the investigator, that will not allow the protocol to be followed safely.
**Planned Sample Size:**	The primary efficacy endpoint "proportion of patients with disease progression on Day 14" is assumed to be 10% in the 300 mg MP1032 BID plus SoC treatment group (Arm A) and 30% in the placebo plus SoC treatment group (Arm B). Using the Chi-square test with type I error alpha=10% two-sided for this proof-of-concept study and 2:1 randomization ratio, with 114 randomized patients (76 in Arm A and 38 in Arm B) a statistical power of 83% can be achieved. Considering about 5% early study terminations, the necessary sample size to randomize results is 120 patients in total (80 in Arm A and 40 in Arm B). If during the conduct of the study the early study termination rate is higher than the estimated 5%, then an increase in the randomized number of patients may be necessary.<br><br>Sample size estimation was performed using nQuery 8, Version 8.6.1.0.<br><br>A subset of randomized patients will be consented for the collection of blood samples for PK measurements. The PK subset consists of approximately 20 patients from the 300 mg MP1032 BID plus SoC group and approximately 10 patients from the placebo plus SoC group, up to a total of approximately 30 patients.
**Study Drug, Dose, and Mode of Administration:**	All randomized patients will receive study drug from Day 1 to Day 28 according to their assigned treatment and according to the randomization scheme. Patients will receive 6 capsules in the morning and 6 capsules in the evening (ie, approximately every 12 hours, approximately at the same time every day, and at least 8 hours apart) according to their randomized treatment arm assignment as described below:<br><br>• Arm A: MP1032 (300 mg [6 × MP1032 hard gelatin capsules 50 mg] BID) for oral administration<br>• Arm B: 6 × placebo capsules (ie, matching MP1032 hard gelatin capsules 50 mg) BID for oral administration
**Standard of Care**	Each site will treat all patients with SoC for the duration of the study. The selected SoC will be used in accordance with the hospital's SoC procedures and may include drugs under an emergency use authorization.
**Treatment Duration:**	Patients will be treated with study drug for 28 consecutive days (unless study drug is discontinued for safety reasons) to provide a fixed duration of treatment across all patients. Patients who progress to the use of an invasive ventilation (mechanical ventilator and/or ECMO) or who can no longer swallow the study drug (>2 days [ie, 2-day interruption is acceptable]) must discontinue the study drug; but whenever possible, the study assessments will be performed on Day 14, Day 21, Day 28, and Day 60. If the patient is discharged home before Day 28, the study team will provide the patient with the remainder of the assigned blinded study drug kit(s) to take home along with instructions on how to continue treatment at home on the day-of-discharge; for these patients, treatment compliance will be conducted via a diary. If the patient does not feel comfortable

	to complete the diary, the site will alternatively provide the patient with the option to be called, twice a day, to confirm that the study drug is taken as instructed.<br><br>The study duration for an individual patient will be as follows:<br>• Screening period: up to 7 days<br>• Treatment period: 28 consecutive days (ie, Day 1 to Day 28)<br>• Follow-up period: 32 days after Day 28 (ie, Day 60)<br><br>As such, the approximate study duration (including screening and the follow-up period) for an individual patient is up to 67 days (±3 days).<br><br>Please note: Patients who remain hospitalized after 28 days of consecutive treatment with study drug are not eligible for additional treatments with study drug.<br><br>Any patient who is discharged during the study should complete the day-of-discharge assessments before release from the hospital.
**Efficacy Assessments:**	The following efficacy assessments will be collected:<br>• COVID-19 symptoms<br>• COVID-19 severity<br>• Clinical status related to COVID-19 on the NIAID 8-point ordinal scale, as well as, each individual component of the NIAID 8-point ordinal scale<br>• HRQoL assessed by the EQ-5D-5L questionnaire<br>• COVID-19 testing
**Safety Assessments:**	The following safety assessments will be collected:<br>• Medical/surgical history<br>• Demographics<br>• Prior and concomitant medications<br>• Vital signs<br>• Physical examinations<br>• Electrocardiograms<br>• Clinical laboratory assessments<br>• AEs
**Pharmacokinetic Assessments:**	PK samples will be collected via sparse sampling from a subset of approximately 30 patients (who give optional consent) in a 2:1 ratio (approximately 20 patients from the 300 mg MP1032 BID plus SoC group and approximately 10 patients from the placebo group plus SoC group) to assess the PK of MP1032 on Day 1 (single dose) and Day 7 (steady state). A total of 16 samples will be collected relative to the first dose on Day 1 and the first dose on Day 7. Samples on Day 1 and Day 7 will be collected predose (within 10 minutes before the first daily dose) and postdose at 10 minutes, 20 minutes, 30 minutes, 60 minutes, 120 minutes, 8 hours (before next dose), and 24 hours before the next morning dose (if applicable), if possible. Patients who provide consent for the PK sampling, but are no longer hospitalized on Day 7, will not have Day 7 PK samples collected.
**Other Assessments:**	Blood samples will be obtained on Day 1 (before the first study drug administration) and Days 7, 14, 21, 28, and 60 (when possible) to measure biomarker levels. Biomarkers will potentially include but will not be limited to

	cytokines (eg, C-reactive protein, IL-1β, IL-6, tumor necrosis factor-α, and IFN-γ) and other coagulation/inflammatory biomarkers (eg, D-dimer and ferritin).
**Statistical Methods and Planned Analyses:**	**Analysis Sets:**<br><br>**Intention-to-Treat Set**<br><br>The Intention-to-Treat (ITT) set, correspond with randomized set, will include all randomized patients irrespective of any deviation from the protocol or premature discontinuation from study drug/withdrawal from study. The treatment group assignment will be designated according to initial randomization. The ITT will serve as the basis for the analysis of efficacy and summary of demographics and baseline characteristics.<br><br>**Per Protocol Set**<br><br>The Per Protocol Set (PPS) will include all patients from ITT who received at least 1 dose of study drug and who do not have any major protocol deviations impacting the efficacy assessments. The PPS will be used for supportive analyses of efficacy.<br><br>**Safety Set**<br><br>The Safety Set (SS) will include all randomized patients who received at least 1 dose of study drug. The treatment group assignment in the SS will be defined by the treatment actually received. The SS will be used for the analysis of safety.<br><br>**PK Analysis Set**<br><br>The PK Analysis Set (PKS) will include all the patients who have been administered active study drug and have at least 1 postdose evaluable plasma concentration after Day 1 dose.<br><br>**Analyses:**<br><br>The estimands for the primary efficacy endpoint and for the 4 key secondary endpoints comparing the 2 treatment groups will be tested with consideration of a two-sided alpha of 10% on each test-level on the ITT. Additionally, 95% confidence intervals will be provided. Also, the treatment comparison with regards to the other efficacy endpoints will be performed using a two-sided alpha level of 10%.<br><br>The main estimand for the primary efficacy endpoint is defined as a binary variable indicating disease progression (death or respiratory failure) at Day 14. Intercurrent events that lead to study drug discontinuation and/or switch to other treatment before Day 14 will be handled using the treatment-policy strategy (ie, the NIAID score at Day 14 will be used for definition of the failure in such patients). Missing data on Day 14 resulting from study termination before Day 14 are not expected. However, in case they happen, such monotone missings will be imputed by multiple imputation using information from similar patients of the same treatment group. Missing post-baseline data for patients randomized but not treated will be imputed by the respective baseline value (baseline observation carried forward, BOCF). The comparison between the treatment groups considering all randomized patients after having imputed missing values will be performed using Mantel-Haenszel (MH) test for risk difference considering the 4

	strata resulting from the 2 randomization stratification factors disease severity (moderate versus severe) and age-class (aged ≤65 years versus >65 years) at baseline.<br><br>Similar MH analyses will be conducted for the main estimand of the first key secondary efficacy endpoint, which is defined as a binary variable indicating disease progression (death or respiratory failure) at Day 28, and the main estimand for the second key secondary efficacy endpoint, which is defined as a binary variable indicating disease resolution (alive and have a score of 6, 7 or 8 on NIAID) at Day 28. Intercurrent events that lead to study drug discontinuation and/or switch to other treatment before Day 28 will be handled using the treatment-policy strategy for these estimands (ie, the NIAID score at Day 28 will be used for definition of the failure or response, respectively) in such patients. Missing data on Day 28 will be imputed by multiple imputation using information from similar patients of the same treatment group.<br><br>A similar MH approach as described above will be applied also to the main estimand of the third key secondary efficacy endpoint, which is the all-cause mortality at Day 28. Intercurrent events that lead to study drug discontinuation and/or switch to other treatment before Day 28, will be handled using the treatment-policy strategy (ie, the life status at Day 28 will be considered). No missing data of life status on Day 28 is expected.<br><br>The main estimand of the fourth key secondary efficacy endpoint, change of clinical status related to COVID-19 on Day 28 compared with baseline according to the NIAID 8-point ordinal scale, will be compared using the risk difference from the analysis of covariance model with treatment and the 2 binary stratification factors (ie, severity and age-class) as independent factors and the baseline value of NIAID as independent covariate. Intercurrent events that lead to study drug discontinuation and/or switch to other treatment before Day 28 will be handled using the treatment-policy strategy for these estimands (ie, the NIAID score at Day 28 will be used). Missing data on Day 28 will be imputed by multiple imputation using information from similar patients of the same treatment group.<br><br>Sensitivity analyses for the main estimands of the primary and key efficacy estimands will be performed by assuming missing data at Day 14 or Day 28 as failure or non-resolution for the binary endpoints, and by multiple imputation using information from similar patients of the placebo group for the NIAID 8-point ordinal scale.<br><br>All above analyses for the main estimands of the primary efficacy and key secondary efficacy endpoints will be repeated on PPS as supportive analysis. Additionally, for the primary efficacy and binary key secondary efficacy endpoints, logistic regression model with treatment and the 2 stratification factors as covariables will be performed on the ITT.<br><br>The proportion of patients with disease resolution at Day 14 and the all-cause mortality rate at Day 14 and Day 60 will be analyzed using similar MH methods as described above for Day 28. Cumulative proportion of patients with disease resolution will be plotted by time.

	A similar analysis of covariance approach as described above for Day 28 will be applied for the analysis of the change of clinical status related to COVID-19 on Day 14 compared with baseline on the NIAID 8-point ordinal scale. The endpoint considering the proportion of patients requiring invasive ventilation (mechanical ventilator and/or ECMO) or who are not alive, the proportion of patients in each NIAID category, and the proportion of patients alive and testing negative for COVID-19 on Day 14, Day 28, and Day 60, respectively, will be compared between the treatment groups using MH test. Overall survival, time to (first) improvement of at least 1 category on the NIAID scale and total hospitalization duration (from baseline to discharge; with death censored on last day of the observed period - at Day 28 or Day 60, respectively - depending on the analyses) will be summarized using the Kaplan-Meier method and will be compared between treatment groups using log-rank tests. Additionally, Cox proportional hazards model will be applied with treatment and the 2 stratification factors as covariables.<br><br>The odds ratio between MP1032 and SoC and placebo and SoC for the number of patients with clinical status improvement from baseline on the NIAID 8-point ordinal scale (ie, an improvement of at least 1 category) at Day 14 and Day 28 will be analyzed using a logistic regression with consideration of the 2 stratification factors.<br><br>The time to recovery of all at baseline present COVID-19 symptoms (stuffy or runny nose, sore throat, red or irritated eyes, shortness of breath, cough, low energy or tiredness, muscle or body aches, headache, chills or shivering, feeling hot or feverish, nausea, and number of times of vomit, times of diarrhea, sense of smell, sense of taste in the last 24 hours) will be presented by Kaplan-Meier estimates and figure. The change from baseline will be presented in shift tables for each single symptom, with the number and percentage of subjects in each category at postbaseline assessment time-point compared to baseline for each treatment group.<br><br>Total number of days in ICU, duration of invasive mechanical ventilation, and duration of ECMO will be descriptively summarized by treatment group using mean days with 90% confidence intervals and additionally by median days and quartiles. To consider death sufficiently unfavorable in these measures, in case of death the duration of the respective measure will be considered with the longest duration possible (eg, if a summary of the first 28 days is presented, then death before Day 28 will be considered as duration 28 days, and if the summary also includes follow-up period until Day 60, then death before Day 60 will be considered as duration of 60 days). Both summaries (up to Day 28 and up to Day 60) will be provided.<br><br>For HRQoL assessments at discharge and Day 60, the EQ index value and the EQ VAS, both based on the EQ-5D-5L questionnaire will be calculated and summarized by visit and treatment group. Additionally, change at Day 60 from discharge by treatment group.<br><br>All efficacy endpoints will be summarized using descriptive statistics by treatment group and by visit, as appropriate: Binary and categorical endpoints will be summarized by treatment group by presenting the number and percentage of

	patients in the categories. Continuous and ordinal endpoints will be described by mean, standard deviation, median, minimum, and maximum for actual values and change from baseline. Additionally, for ordinal data, the number and percentage of patients in each category will be presented.<br><br>The PK parameters (such as C_{max}, T_{max}, $AUC_{0\text{-}t}$, and others) of MP1032 on Day 1 (single dose) and Day 7 (steady state; if available), using up to 8 hours postdose, will be derived applying non-compartmental analysis. PK parameters will be summarized along with plasma concentrations at various time points. Individual and mean plasma concentration versus time will be presented on linear, and semi-log scales. Further details of the PK analysis will be described in the statistical analysis plan (SAP). Any possible correlation between plasma exposure and biomarkers (eg, change from baseline in cytokines, or other coagulation/inflammatory biomarkers) or efficacy measures can be explored by appropriate regression analysis methods during the final data analysis.<br><br>The change from baseline biomarker levels will be summarized descriptively by treatment group and visit. The change in biomarker levels will also be evaluated with any change of COVID-19 clinical status.<br><br>All safety parameters will be summarized descriptively by treatment group for the entire study period.<br><br>Study drug duration and SoC therapies will be described for each treatment group.<br><br>The SAP will present further estimands for the primary and key secondary endpoints and will describe the statistical analyses in detail.
**Independent Data Monitoring Committee:**	An independent data monitoring committee (DMC) will be established by the sponsor or designee to review accumulating study data at regular intervals (as per the DMC charter) throughout the study to ensure the safety of patients and review overall study conduct. Additional data may be reviewed that are related to efficacy, but the DMC will only be reviewing data for risk-benefit purposes. Members will include experts in drug safety and/or medical specialist and biostatistics, who are not participating in this study and do not have affiliation with the investigators or the sponsor. The DMC can recommend in writing to the sponsor whether to continue, modify, or stop the clinical study on the basis of safety considerations. The DMC’s specific duties will be fully described in a DMC charter.

3 TABLE OF CONTENTS

1 CLINICAL STUDY PROTOCOL 1
2 SYNOPSIS 4
3 TABLE OF CONTENTS 18
3.1 List of In-text Tables 21
4 LIST OF ABBREVIATIONS 22
5 INTRODUCTION 24
5.1 Background on COVID-19 24
5.2 Background on MP1032, Including Benefit Risk Assessment 25
5.2.1 Nonclinical Studies 26
5.2.2 Clinical Studies 27
5.3 Risk Management of MP1032 29
5.4 Study Rationale 29
6 STUDY OBJECTIVES AND ENDPOINTS 31
6.1 Study Objectives 31
6.2 Study Endpoints 31
7 INVESTIGATIONAL PLAN 33
7.1 Description of Overall Study Design and Plan 33
7.2 Discussion of Study Design 36
7.3 End of Study 37
8 SELECTION OF STUDY POPULATION 37
8.1 Inclusion Criteria 37
8.2 Exclusion Criteria 39
8.3 Rescreening 41
8.4 Study Withdrawal, Removal, and Replacement of Patients 41
9 TREATMENTS 42
9.1 Details of Study Drugs 42
9.2 Dosage Schedule 42
9.3 Measures to Minimize Bias: Randomization and Blinding 43
9.3.1 Randomization 43
9.3.2 Blinding44
9.4 Treatment Modifications and Discontinuations 45
9.5 Treatment Accountability and Compliance 46
9.6 Prior and Concomitant Medications 46

10 STUDY PROCEDURES 47
10.1 Informed Consent 53
10.2 Study Procedures 53
11 EFFICACY ASSESSMENTS 53
11.1 COVID-19 Symptoms 54
11.2 COVID-19 Severity 54
11.3 Clinical Status Related to COVID-19 on the National Institute of Allergy and Infectious Diseases 8-Point Ordinal Scale 56
11.4 Health-related Quality of Life Assessment 58
11.5 COVID-19 Testing 58
12 SAFETY ASSESSMENTS 58
12.1 Medical/Surgical History and Demographics 58
12.2 Vital Signs 59
12.3 Physical Examinations 60
12.4 Electrocardiograms 60
12.5 Clinical Laboratory Assessments 60
12.6 Adverse Events 62
12.6.1 Adverse Events 62
12.6.2 Serious Adverse Events 64
12.6.3 Serious Adverse Event Reporting 65
12.6.4 Suspected Unexpected Serious Adverse Reactions 66
12.6.5 Pregnancy 66
12.6.6 Overdose 67
13 PHARMACOKINETICS 67
13.1 Pharmacokinetic Sampling 67
13.2 Pharmacokinetic Analytical Methodology 68
14 OTHER ASSESSMENTS 68
14.1 Biomarkers 68
15 STATISTICAL ANALYSIS 69
15.1 Determination of Sample Size 69
15.2 Analysis Sets 70
15.3 Efficacy Analysis 70
15.4 Safety Analysis 73
15.5 Pharmacokinetic Analysis 74
15.6 Biomarker Analysis 75
15.7 Interim Analysis 75

15.8 Data Monitoring Committee....75
16 STUDY MANAGEMENT....75
16.1 Approval and Consent....75
16.1.1 Regulatory Guidelines....75
16.1.2 Institutional Review Board/Independent Ethics Committee....76
16.1.3 Informed Consent....76
16.2 Data Handling....76
16.3 Source Documents....76
16.4 Record Retention....77
16.5 Monitoring....77
16.6 Quality Control and Quality Assurance....77
16.7 Protocol Amendment and Protocol Deviation....78
16.7.1 Protocol Amendment....78
16.7.2 Protocol Deviations....78
16.8 Ethical Considerations....78
16.9 Financing and Insurance....78
16.10 Publication Policy/Disclosure of Data....79
17 REFERENCES....80
18 APPENDICES....82
APPENDIX 1. CHARLSON COMORBIDITY INDEX....83
APPENDIX 2. EUROQOL-5D-5L (EQ-5D-5L) QUESTIONNAIRE....84

3.1 List of In-text Tables

Table 1: Schedule of Assessments 48

Table 2 COVID-19 Symptoms....54

Table 3: Acute Respiratory Distress Syndrome – Berlin Definition....56

Table 4 Standard Conversion for Liter Flow Oxygen....57

Table 5: Clinical Laboratory Assessments....61

Table 6: Classification of Adverse Events by Severity....63

Table 7: Classification of Adverse Events by Relationship to Study Drug....64

This document is confidential.

4 LIST OF ABBREVIATIONS

**Abbreviation**	**Definition**
AE	adverse event
ARDS	acute respiratory distress syndrome
AUC_{0-t}	area under the concentration-time curve from time zero to the last non-zero concentration
BID	twice daily
BOCF	baseline observation carried forward
CFR	Code of Federal Regulations
C_{max}	maximum plasma concentration
COPD	chronic obstructive pulmonary disease
COVID-19	coronavirus disease 2019
CSR	clinical study report
DMC	data monitoring committee
ECG	electrocardiogram
ECMO	extracorporeal membrane oxygenation
eCRF	electronic case report form
EDC	electronic data capture
EQ	EuroQol
EQ-5D-5L	EuroQol-5D-5L
FiO_2	fraction of inspired oxygen
GCP	good clinical practice
hCG	human chorionic gonadotropin
HRQoL	health-related quality of life
HRT	hormone replacement therapy
IB	investigator’s brochure
ICF	informed consent form
ICU	intensive care unit
IEC	independent ethics committee
IL	interleukin
IMP	investigational medicinal product
IPF	idiopathic pulmonary fibrosis
IRB	institutional review board
ITT	Intention-to-Treat
IWRS	interactive web-response system
LAR	legally authorized representative
MH	Mantel-Haenszel
NIAID	National Institute of Allergy and Infectious Diseases
PAH	pulmonary arterial hypertension
PaO_2	partial pressure of oxygen

**Abbreviation**	**Definition**
PARP1	poly-ADP-ribose-polymerase 1
PASI	Psoriasis Area and Severity Index
PI	principal investigator
PK	pharmacokinetic
PKS	PK Analysis Set
PPS	Per Protocol Set
PT	preferred term
ROS	reactive oxygen species
RT-PCR	reverse transcription-polymerase chain reaction
SAE	serious adverse event
SAP	statistical analysis plan
SARS-CoV-2	severe acute respiratory syndrome coronavirus type 2
SoC	standard of care
SOC	system organ class
SpO_2	saturation of oxygen
SS	Safety Set
SUSAR	suspected unexpected serious adverse reactions
TEAE	treatment-emergent adverse events
T_{max}	time of observed C_{max}
TNF	tumor necrosis factor
VAS	visual analog scale
WHO	World Health Organization
WOCBP	women of childbearing potential

5 INTRODUCTION

5.1 Background on COVID-19

On 31 December 2019, the World Health Organization (WHO) was informed of a cluster of cases of pneumonia of unknown cause detected in Wuhan City, Hubei Province of China. Severe acute respiratory syndrome coronavirus type 2 (SARS-CoV-2) was identified as the causative virus by Chinese authorities on 07 January 2020 (WHO 2020a). Coronavirus disease 2019 (COVID-19) was identified as the disease caused by SARS-CoV-2 infection. In March 2020, the COVID-19 outbreak was declared as a pandemic by the WHO. The disease resulting from SARS-CoV-2 led to shutdowns and social measures in great parts of the world, particularly to slow down the transmission of the virus and thereby avoiding the overburden of health care systems.

The pandemic is still ongoing. Estimates of actual morbidity, mortality, and lethality are highly variable and depend on factors such as the time of analysis, region analyzed, and surveillance methods. As of 04 August 2020, the WHO estimated the case fatality ratio, which estimates the proportion of deaths among confirmed cases, to vary from 0.1% to >25%. Whereas, the true severity of COVID-19 as measured by the infection fatality ratio, which estimates the proportion of deaths among all infected individuals, is estimated to be approximately 0.5% to 1% (WHO 2020b). In a more recent summary of Levin et al (2020) it could be clearly shown that the infection fatality ratio is highly age dependent, being in average as low as 0.004% for patients younger than 35 and as high as 28.3% in patients at the age of 85 and older.

Presentations of COVID-19 range from an asymptomatic course or mild symptoms to severe illness and mortality. Common symptoms include fever or chills, cough, shortness of breath or difficulty breathing, fatigue, muscle or body aches, headache, loss of taste or smell, sore throat, congestion or runny nose, nausea or vomiting, and diarrhea (Cennimo 2021). Less common symptoms are sputum production, malaise, respiratory distress, and neurologic symptoms, such as altered mental state. While most patients with COVID-19 seem to show mild respiratory symptoms only, some of them develop pneumonia, shortness of breath and hypoxia, or other serious conditions that require hospitalization. Generally, pneumonia seems to be the most common serious manifestation of COVID-19. Further, major deterioration such as an acute respiratory distress syndrome (ARDS) can lead to the imperative need of an intensive care unit (ICU) and might even require mechanical ventilation via intubation. Other complications that might occur during the course of the disease include sepsis, acute kidney injury, thyrotoxicosis, and neurologic manifestations such as myasthenia gravis. The elderly, as well as patients with pre-existing chronic conditions (eg, asthma, chronic obstructive pulmonary disease [COPD], diabetes mellitus, chronic kidney disease, immune system deficiencies, liver disease, and cardiac diseases, etc), seem to be more vulnerable to a severe disease course.

Although, to date, knowledge about SARS-CoV-2 and corresponding COVID-19 is still limited, it seems to be clear that the course of the disease, and particularly severe complications, are

based on an overshooting immune response. Details on and a deeper understanding of how the virus causes such a reaction will be a central part of future investigations (Hillienhof 2020).

Patients with COVID-19 show increased levels of plasma pro-inflammatory cytokines (Rothan and Byrareddy 2020). From a pathophysiological point of view, in severe cases the viral infection seems to trigger an excessive immune reaction in the host, a so-called cytokine storm, leading to extensive tissue damage. A key player of this mechanism is interleukin (IL)-6. IL-6 inhibitors, as well as other immune modulators, must thus be considered a potential treatment approach. This approach seems to be particularly promising, as recent postmortem findings show direct viral infection of the endothelial cells and diffuse endothelial inflammation in the whole body (Varga et al 2020).

Recently the first vaccines to prevent COVID-19 have been approved by authorities, including the Food and Drug Administration in the United States and the European Medicines Agency in Europe, and numerous further vaccine projects are still under development. Nonetheless, it will take years until the majority of people will be vaccinated and the epidemiological situation can be considered as under control. Hence, there is still an urgent need for therapeutic agents to cure COVID-19 and/or ameliorate its symptoms. Among the antiviral treatment options, remdesivir has shown to be effective in reducing hospitalization time when administered early in the course of the disease (Beigel et al 2020); however, in the meantime the WHO has issued a conditional recommendation against the use of remdesivir in hospitalized patients regardless of disease severity (WHO 2020c) Among immune modulating drugs low-dosed dexamethasone could significantly lower the incidence of deaths when applied at a later time point of the disease course (WHO-REACT 2020) but is not recommended early in disease or in patients with mild symptoms only (WHO 2020c). Hence, there is still no antiviral treatment or otherwise effective curative agent that could really lower the burden of this pandemic. Thus, COVID-19 still presents an urgent unmet medical need.

5.2 Background on MP1032, Including Benefit Risk Assessment

MP1032 is a purely synthetic, new chemical entity. MP1032 is a mono sodium salt derived from the aromatic system phthalazinedione, containing an additional amino group. MP1032 is water-soluble and hydrophilic.

The primary mechanism of action of MP1032 is assumed to be a reactive oxygen species (ROS) scavenging action in inflamed tissues (ie, tissues that experience oxidative stress). A unique pharmacologic property of MP1032 is that the ROS-scavenging function of the molecule is only activated in tissues with pathologically high ROS concentrations (oxidative stress), which is the case in inflamed tissue. By re-establishing a physiologic redox balance in activated immune cells, MP1032 is putatively inhibiting nuclear factor-kappa B activation. Inhibition of nuclear factor-kappa B is expected to lead to inhibition of pro-inflammatory cytokines (eg, IL-6 and TNF-α) (Gloire et al 2006). In addition, MP1032 is a specific inhibitor of poly-ADP-ribose-polymerase 1 (PARP1). PARP1 is a nuclear enzyme involved in inflammation (Ke et al 2019).

This document is confidential.

Further identified potential targets are the kinases human Aurora-B, human Aurora-C, serine/threonine-protein kinase, tyrosine kinase, and the metalloprotease TNF-α-converting enzyme—however, at relatively high molarity only. Therefore, the administration of MP1032 (as shown nonclinically, both in vitro and in vivo), in pathological conditions only, leads to a reduced concentration of pro-inflammatory cytokines without completely blocking them. In addition to this broadly anti-inflammatory potential, MP1032 exhibits direct antiviral activity against SARS-CoV-2 that could be shown by a clear and dose-dependent inhibition of SARS-CoV-2 reproduction in Vero B4 cells (African green monkey [*Chlorocebus aethiops*] kidney epithelium cells) (Schumann et al 2020).

MP1032 could attenuate prolonged virus replication by preventing oxidative stress (Khomich et al 2018) and/or by limiting ADP-ribosylation of the viral nucleocapsid protein via PARP1 inhibition (Grunewald et al 2018). PARP1 inhibitors also have been suggested for the treatment of COVID-19 (Curtin et al 2020).

Based on the basic chemical mechanism of MP1032 as an electron donor to ROS, the molecule acts similarly across a wide range of species and is thus not an agent with a high degree of species specificity. Further, MP1032 has no specific immune-system targets as known from various biologicals. MP1032 is not expressed by using mammalian systems (cells) or using raw materials of human or animal origin.

5.2.1 Nonclinical Studies

In vivo safety studies showed no evidence of neurotoxic potential or negative cardiohemodynamic effects; no local intolerance was observed. There was no dose-limiting or target organ toxicity and no substance-related findings in histopathology in rats or dogs. However, after 6 months of long-term treatment in rats receiving the highest dose (1000 mg/kg/d), liver organ weight was elevated compared with that of controls and histopathologically associated with hepatocellular hypertrophy. This finding was not considered adverse because of its adaptive character, as histopathological changes were not present at the end of the recovery period (ie, effect was reversible after 8 weeks).

In reproduction toxicity studies on fertility, early embryonic, and embryo-fetal development, no pathological effects were found. On the basis of results from 4 different test systems, mutagenic or clastogenic effects are not considered to be a potential risk for volunteers and patients.

Transient effects on some pulmonary parameters have been observed in rats following a single intravenous bolus injection of the highest dose (300 mg/kg) of MP1032. No pulmonary effects have been observed in any of the long-term animal toxicity studies. The maximum plasma levels reached in humans are over 1000 times (oral) lower than for the abovementioned dose. Thus, pulmonary effects are not considered a potential risk for patients and volunteers.

Emesis sporadically occurred in various repeat-dose toxicology studies in dogs; the emesis was present in the dogs more often at the high doses of 150 mg/kg twice daily (BID) intravenous than at 125 mg/kg BID (oral). The lowest experimental oral dose inducing emesis was 25 mg/kg BID

(oral) in dogs, corresponding to 1.8 g (oral; conversion based on human-equivalent dose) for a 65-kg human. Dogs are well known to be susceptible to emesis; in addition, the maximum doses administered in humans are 6 to 30 times below the converted human-equivalent doses. Therefore, emesis is not considered a potential risk for patients and volunteers.

Transient liquid feces were sporadically observed in various repeat-dose toxicology studies in dogs of all dose groups treated with MP1032. However, these gastrointestinal irritations could not be attributed to specific animals and thus did not have an observable effect on their wellbeing. Further, no such observations were made in any of the other species investigated. Also, in the clinical studies completed so far, no cases of diarrhea have been reported as adverse drug reactions by the respective investigators. Gastrointestinal irritations are therefore not considered a potential risk for patients and volunteers.

A potential phototoxic risk cannot be excluded from the in vitro 3T3 neutral red uptake and the distribution quantitative whole-body autoradiography test. In the clinical studies completed so far with MP1032, mean maximum plasma concentrations and calculated maximum skin concentrations were all below the half maximal effective concentration in the 3T3 neutral red uptake test with safety margins between 2 to 20 times. Therefore, a phototoxic risk for patients seems to be unlikely.

Further, a slight to moderate increase in serum creatinine level combined with a slight decrease of kidney weights was observed after a 90-day treatment period in male mice in a dose-range finding, repeated dose (oral) toxicity study at high dosed animals. However, as there were no associated histopathological findings in kidneys of the respective animals, this effect is also not considered to be adverse in nature. In the scope of extensive laboratory controls in the clinical studies completed so far, no respective treatment-emergent adverse events (TEAEs) have been reported.

The probability of pharmacokinetic interactions of MP1032 regarding the inhibition or induction of Cytochrome P450 or inhibition of well-known drug transporters is rather low. However, based on in vitro experiments there are hints that a combination of MP1032 and dexamethasone might act (supra-)additive regarding the inhibition of proinflammatory cytokines (ie, IL-6). Thus, currently, a potential drug-drug interaction of MP1032 and dexamethasone regarding their anti-inflammatory action cannot be excluded.

For further details of the completed nonclinical studies conducted with MP1032, readers are referred to the current version of the investigator’s brochure (IB).

5.2.2 Clinical Studies

MP1032 so far has been investigated in 3 clinical studies, including a first-in-human study (MP1032-CT01; EudraCT-No.: 2014-004606-15) with a single ascending dose and a multiple ascending dose part, as well as 2 studies in patients with moderate-to-severe psoriasis: a 6-week, Phase IIa, pilot study (MP1032-CT02; EudraCT-No.: 2015-005159-28) and a 3-month, Phase II study (MP1032-CT04; EudraCT-No.: 2017-003484-36).

Among the subjects enrolled in MP1032 studies, 146 subjects were administered MP1032 orally. Among them, 24 male subjects participated as healthy volunteers in the Phase I study, while both male (n=89) and female (n=33) patients with psoriasis participated in the Phase II studies. The age of participants administered MP1032 ranged from 18 to 68 years. Pregnant and breastfeeding women, as well as children and adolescents have not been included in the studies.

The highest single dose of MP1032 administered was 600 mg, applied to healthy volunteers. The highest repeated dose was 300 mg MP1032 BID, applied to both healthy volunteers (7 days) and patients with psoriasis (3 months).

No serious adverse events (SAEs) occurred in any of the subjects administered MP1032.

No deaths occurred in the scope of the MP1032 clinical development program.

MP1032 was well tolerated in all studies, and safety analyses did not reveal any clinically important safety issues. In general, incidence, intensity, causality, and spreading of TEAEs over system organ classes (SOCs) were comparable between groups. In the recent Phase II study, the incidence, intensity, relationship to investigational medicinal product (IMP), and causality to IMP for adverse events (AEs) were even lower for both MP1032 dose groups compared with those of the placebo group and statistically significantly lower for the 300 mg MP1032 group compared with the placebo group.

None of the potential risks arising from preclinical data could be confirmed with clinical data.

From the TEAEs that occurred in the MP1032 groups and were regarded as at least possibly related by the investigator, nasopharyngitis, pruritus, and psoriasis must be considered as common (≥1/100 to <1/10); while neutropenia, palpitations, abdominal pain upper, fatigue, feeling drunk, influenza-like illness, cystitis, and headache can be regarded as uncommon (≥1/1,000 to <1/100). Regarding the general spreading of AEs over SOCs and the comparison between groups, currently none of them can be considered a significant clinical risk.

Regarding efficacy in the treatment of moderate to severe psoriasis, the MP1032-CT02 study showed that after only 6 weeks of treatment, there was a clinically meaningful response in patients who entered the study with moderate to severe psoriasis with a Psoriasis Area and Severity Index (PASI) score of 10 to 20 and achieved the appropriate drug-exposure levels. Hence, only patients with a PASI score of 10 to 20 at baseline were randomized to the MP1032-CT04 study. After 12 weeks, a significant PASI reduction could be shown in the 300 mg MP1032 BID group compared with those in the placebo group; however, this only occurred in a prespecified subgroup of patients with PASI ≤15 at baseline.

Further details of the completed clinical studies conducted with MP1032 may be found in the current version of the IB.

5.3 Risk Management of MP1032

In the scope of planning and conducting future clinical studies, all potential risks associated with MP1032 will be considered, and measures will be taken as applicable.

On the basis of limitations in the amount of preclinical and clinical data available so far, MP1032 shall not be administered to patients for >3 months, and it will generally not be administered to children or pregnant women.

Adverse drug reactions that have already been described in the clinical program so far, as well as any suspected AEs arising from risks associated with preclinical data will be evaluated extensively on a case-by-case base during ongoing and/or upcoming studies.

Because of potential phototoxicity, patients in upcoming studies will be advised to avoid strong sun exposure (sunbathing) and strong ultraviolet exposure. Furthermore, the use of sunscreen will be recommended when spending an extended period outdoors. It should be ensured that exposure to ultraviolet light (eg, from lasers in procedures) is limited while the patient is hospitalized.

To avoid risks associated with potential low efficacy in serious and life-threatening conditions, such as COVID-19, MP1032 is only recommended to be used as add-on to standard of care (SoC) until a respective proof-of-concept is demonstrated.

As dexamethasone is expected to be part of SoC in most institutions treating patients with COVID-19 investigators should be aware that a (supra-)additive synergism between MP1032 and dexamethasone regarding their anti-inflammatory activity cannot be excluded.

Further details regarding known or anticipated risks, as well as reasonably anticipated AEs for MP1032 may be found in the current version of the IB.

5.4 Study Rationale

MP1032 is a small molecule that combines localized, auto-regulated ROS-scavenging and immune-modulating effects with potential antiviral properties against SARS-CoV-2. MP1032 leads to a reduced concentration of pathologically increased pro-inflammatory cytokines without completely blocking them. This downregulation to physiologic levels was shown in vivo and in vitro for IL-1β, IL-6, IL-12, IL-23, and TNF-α in activated macrophages/neutrophils; most data are available on the downregulation of IL-6 and TNF-α. Once physiologic ROS levels have been reached, concurrent with down-modulated cytokine levels, the drug activation stops. Further, MP1032 might suppress virus replication in patients with COVID-19 dose-dependently like it has been shown in vitro with no effect on cell viability.

On the basis of this mechanism of action, MP1032 does not carry the risk of developing opportunistic infections, such as with IL-6 or TNF-α inhibitors. On the contrary, an in vivo study on polymicrobial abdominal sepsis in mice showed a decrease of bacterial burden in animals treated with MP1032, although a direct antibacterial effect for MP1032 could not be shown.

MP1032 has also been investigated in a small exploratory hemagglutinin type 5 neuraminidase type 1 virus infection model in mice, wherein animals treated with MP1032 showed a significantly improved clinical score on Days 6 and 7 after infection, combined with a clear trend of prolonged survival compared with that of the mice receiving placebo.

In summary, when administered in combination with or as add-on to standard treatments, MP1032 may prevent and/or ameliorate any overstimulation of the immune system in the course of a COVID-19, thereby reducing the danger of opportunistic infections and helping to reduce the severity of general inflammatory symptoms. At the same time, the probability of a severe course of the disease is reduced because of a potential direct antiviral capacity of MP1032. This combinative approach makes MP1032 a promising candidate for the treatment of COVID-19. In addition, synergistic effects may be obtained when using MP1032 as an add-on to other medicinal products currently under investigation for treating patients with COVID-19.

There is a substantial medical need for preventive and/or therapeutic options for the COVID-19 pandemic. COVID-19 is a viral disease with a considerable mortality rate that is not yet completely determined, and it significantly affects healthcare systems. Considering the strong safety profile and good tolerability of MP1032 based on both preclinical and clinical data, combined with the signals of meaningful therapeutic effects of MP1032, it is justified that a proof-of-concept for MP1032 in the treatment of patients with COVID-19 is started.

As such, this proof-of-concept study is designed to investigate the efficacy and safety of MP1032 plus SoC versus placebo plus SoC treatment in hospitalized adults with confirmed moderate to severe COVID-19, the disease caused by SARS-CoV-2 infection. Additional exploratory objectives include pharmacokinetics (PK) of MP1032 in a PK subset of patients and the effect of MP1032 on biomarker levels.

This study will be executed during a worldwide outbreak of COVID-19, resulting in capacity issues at many medical facilities and concerns about the safety of healthcare providers caring for these seriously sick patients. Consequently, this study is designed to minimize the burden on clinical sites, including minimizing the number of required assessments and interventions. To allow close monitoring and oral administration, the intended patient population shall include only hospitalized patients with COVID-19 that do not require intubation at the time of screening and/or randomization. Only adults shall be included. Additionally, pregnant and breastfeeding women shall be excluded.

The study drugs will be indistinguishable to blinded study personnel to limit bias in study assessments. Most interventions and assessments may be accomplished in conjunction with the schedule dictated by SoC. An independent data monitoring committee (DMC) will be established by the sponsor or designee to review accumulating study data at regular intervals (as per the DMC charter) throughout the study to ensure the safety of patients and review overall study conduct.

This document is confidential.

6 STUDY OBJECTIVES AND ENDPOINTS

6.1 Study Objectives

The primary objective of this study is:

- To measure the effect of MP1032 plus SoC versus placebo plus SoC on Day 14 on disease progression in patients with moderate to severe COVID-19

The secondary objectives of this study are:

- To measure the effect of MP1032 plus SoC versus placebo plus SoC on Day 28 on disease progression in patients with moderate to severe COVID-19
- To measure the effect of MP1032 plus SoC versus placebo plus SoC on disease resolution on Day 14 and Day 28
- To measure the effect of MP1032 plus SoC versus placebo plus SoC on the mortality rate and other specific COVID-19 related characteristics
- To assess the safety of MP1032 (eg, AEs and laboratory abnormalities)
- To assess the PK of MP1032 on Day 1 (single dose) and Day 7 (steady state) in a PK subset of patients

The exploratory objectives of this study are:

- To measure the effect of MP1032 plus SoC versus placebo plus SoC on some additional COVID-19 related characteristics
- To evaluate the health-related quality of life (HRQoL) of patients treated MP1032 plus SoC compared with placebo plus SoC
- To evaluate biomarker levels

6.2 Study Endpoints

The primary efficacy endpoint of this study is:

- Proportion of patients with disease progression on Day 14. Disease progression is defined as the proportion of patients who are not alive or who have respiratory failure. Respiratory failure is defined as patients who have a score of 2, 3, or 4 on the National Institute of Allergy and Infectious Diseases (NIAID) 8-point ordinal scale (see below).

The key secondary efficacy endpoints of this study are:

- Proportion of patients with disease progression on Day 28. Disease progression is defined as the proportion of patients who are not alive or who have respiratory failure. Respiratory failure is defined as patients who have a score of 2, 3, or 4 on the NIAID 8-point ordinal scale (see below).
- Proportion of patients with disease resolution on Day 28. Disease resolution is defined as patients who are alive and have a score of 6, 7, or 8 on the NIAID 8-point ordinal scale.

- All-cause mortality rate at Day 28
- Change of clinical status related to COVID-19 on Day 28 compared with baseline according to the following NIAID 8-point ordinal scale:
  1. Death
  2. Hospitalized, on invasive ventilation (mechanical ventilator and/or extracorporeal membrane oxygenation [ECMO])
  3. Hospitalized, on non-invasive ventilation or high-flow oxygen devices
  4. Hospitalized, requiring supplemental oxygen
  5. Hospitalized, not requiring supplemental oxygen, but requiring ongoing medical care (COVID-19 related or otherwise)
  6. Hospitalized, not requiring supplemental oxygen and no longer requires ongoing medical care (used if hospitalization was extended for infection-control reasons)
  7. Not hospitalized, limitation on activities, and/or requiring home oxygen
  8. Not hospitalized, no limitations on activities

Please note: Patients requiring oxygen before COVID-19 and returning to baseline oxygen use will be considered improved (ie, not requiring supplemental oxygen). Patients with a limitation on activities before COVID-19 and returning to baseline activity will be considered improved. In case of death before Day 14 or Day 28, the patient will be considered with the NIAID score for death (score of 1) on the date of death in the analysis.

The other secondary endpoints of this study are:

- Proportion of patients with disease resolution on Day 14. Disease resolution is defined as patients who are alive and have a score of 6, 7, or 8 on the NIAID 8-point ordinal scale.
- All-cause mortality rate at Day 14 and Day 60
- Change of clinical status related to COVID-19 on Day 14 compared with baseline according to the NIAID 8-point ordinal scale as listed above
- Proportion of patients requiring invasive ventilation (mechanical ventilator and/or ECMO), or who are not alive on Day 14 or Day 28
- Proportion of patients in each category of the NIAID 8-point ordinal scale
- Time to (first) improvement of at least 1 category on the NIAID 8-point ordinal scale (until Day 28). Patients who did not improve at least 1 category on the NIAID scale or die before Day 28 will be censored at Day 28.
- The odds ratio between MP1032 and SoC and placebo and SoC for the number of patients with clinical status improvement from baseline on the NIAID 8-point ordinal scale (ie, an improvement of at least 1 category) at Day 14 and Day 28
- Total duration of hospitalization on Day 28 and Day 60 (from baseline to discharge; with death censored on the last day of the observed period – at Day 28 or Day 60, respectively)

- Proportion of patients alive and testing negative for COVID-19 on Day 14, Day 28, and Day 60
- Safety and tolerability assessed by:
  - Cumulative incidence of TEAEs (summarized by seriousness, severity, relationship to the study medication, outcome, and duration)
  - Vital sign parameters
  - Clinical laboratory parameters
  - Physical examination findings
- MP1032 plasma concentrations and PK parameters (if possible) including maximum observed plasma concentration, area under the concentration-time curve, elimination parameters, apparent body clearance, apparent volume of distribution, trough concentration, average observed plasma concentration at steady state, and other relevant PK parameters assessed via MP1032 plasma exposure on Day 1 and Day 7 in a PK subset of patients

Exploratory endpoints of this study are:

- Change in saturation of oxygen (SpO_2)/fraction of inspired oxygen (FiO_2) ratio (for patients alive) on Day 14 or Day 28 compared with baseline
- Total number of days in the intensive care unit (ICU)
- Duration of invasive mechanical ventilation
- Duration of ECMO
- Time to recovery from COVID-19 symptoms (stuffy or runny nose, sore throat, red or irritated eyes, shortness of breath, cough, low energy or tiredness, muscle or body aches, headache, chills or shivering, feeling hot or feverish, nausea, and number of times of vomit, times of diarrhea, sense of smell, sense of taste in the last 24 hours) at Day 14, Day 28, and Day 60
- Change from discharge in the EuroQol (EQ) index value and EQ visual analog scale (VAS) based on the EuroQol-5D-5L (EQ-5D-5L) questionnaire (Appendix 2) at Day 60
- Change from baseline in biomarker levels potentially including, but not limited to, cytokines (eg, C-reactive protein, interleukin [IL]-1β, IL-6, TNF-α, and IFN-γ), and other coagulation/inflammatory biomarkers (eg, D-dimer and ferritin)

7 INVESTIGATIONAL PLAN

7.1 Description of Overall Study Design and Plan

This is a Phase IIa, randomized, double-blind, placebo-controlled, multicenter, proof-of-concept study designed to assess the efficacy and safety of 300 mg MP1032 BID plus SoC versus placebo plus SoC in hospitalized adults with moderate to severe COVID-19.

Approximately 40 sites worldwide will participate in this study.

Approximately 140 patients will be screened to randomize approximately 120 patients (see Section 15.1 for determination of sample size) in 2:1 ratio as follows:

- Arm A (300 mg MP1032 BID plus SoC): approximately 80 patients
- Arm B (placebo BID plus SoC): approximately 40 patients

The stratification factor for randomization will include baseline COVID-19 severity (moderate versus severe) and age-class (aged ≤65 years versus >65 years). COVID-19 severity will be determined using the following criteria:

- Moderate COVID-19:
  - Positive SARS-CoV-2 testing by standard reverse-transcription polymerase chain reaction (RT-PCR) assay or equivalent test
  - Symptoms of moderate illness with COVID-19, which could include any symptom of mild illness or shortness of breath with exertion
  - Clinical signs suggestive of moderate illness with COVID-19, such as respiratory rate ≥20 breaths per minute, SpO_2 >93% (on room air at sea level, if possible), heart rate ≥90 beats per minute
  - No clinical signs indicative of severe or critical COVID-19

- Severe COVID-19:
  - Positive SARS-CoV-2 testing by standard RT-PCR assay or equivalent test
  - Symptoms suggestive of severe systemic illness with COVID-19, which could include any symptom of moderate illness or shortness of breath at rest, or respiratory distress
  - Clinical signs indicative of severe systemic illness with COVID-19, such as respiratory rate ≥30 breaths per minute, heart rate ≥125 beats per minute, SpO_2 ≤93% (on room air at sea level, if possible), partial pressure of oxygen (PaO_2)/FiO_2 <300, or diagnosed with ARDS (according to the Berlin definition; see Table 3
  - No criteria met for critical COVID-19

To standardize the assessment of COVID-19 severity, respiratory rate, SpO_2, and heart rate will be measured when the patient is on room air at sea level (ie, no supplemental oxygen, if possible) and at rest for at least 5 minutes. If possible, the site should collect the information from each patient at the same time each day (±1 hour).

Please note: Patients who are receiving oxygen therapy at baseline for a chronic condition (eg, emphysema, COPD, pulmonary arterial hypertension [PAH], idiopathic pulmonary fibrosis [IPF], etc) must be considered as having severe COVID-19 (unless the patient meets the definition for critical COVID-19). For questions related to COVID-19 severity, the investigator should contact the medical monitor.

Each site will treat all patients with SoC for the duration of the study. The selected SoC will be used in accordance with the hospital's SoC procedures and may include drugs under an emergency use authorization.

This study consists of 3 periods: (1) screening, (2) treatment, and (3) follow-up. During the screening period, each potential patient (or patient's legally acceptable representative) will provide informed consent before starting any study-specific procedures. The randomization of patients to treatment groups will be performed centrally by an interactive web-response system (IWRS) using a randomization scheme that will be developed by an unblinded, independent statistician. During the treatment period, randomized patients will be provided their assigned treatment and assessments according to the protocol. All patients will be treated for 28 days unless the study drug is discontinued for safety reasons. If the patient is discharged home before Day 28, the study team will provide the patient with the remainder of the assigned blinded study drug kit(s) to take home along with instructions on how to continue treatment at home on the day-of-discharge; for these patients, treatment compliance will be conducted via a diary. If the patient does not feel comfortable to complete the diary, the site will alternatively provide the patient with the option to be called, twice a day, to confirm that the study drug is taken as instructed. The treatment period will end with the Day 28 (End of Treatment Visit) assessments. The follow-up period will consist of Day 60 (Follow-up Visit) assessments.

Please note: Patients will be assessed while hospitalized. If patients are discharged from the hospital, they can have study visits at Day 14, Day 21, Day 28, and Day 60 as an outpatient visit. Patients discharged early will continue to receive study drug through Day 28. For discharged patients, Day 14 and Day 28 visits are preferred to be conducted in person at the study site. If restrictions limit the ability of the patient to return to the study site, these visits may be conducted by home healthcare visit or by telephone/virtual call (the patient's caregiver may assist during the telephone/virtual call). For discharged patients, it is sufficient to conduct the Day 60 Follow-up Visit as a telephone/virtual call (the patient's caregiver may assist during the telephone/virtual call) or an outpatient or home healthcare visit is also allowed.

Note: Home healthcare visits are only allowed in countries where home healthcare visits are explicitly permitted by regulatory requirements.

The study duration for an individual patient will be as follows:

- Screening period: up to 7 days
- Treatment period: 28 consecutive days (ie, Day 1 to Day 28)
- Follow-up period: 32 days after Day 28 (ie, Day 60)

As such, the approximate study duration (including screening and the follow-up period) for an individual patient is up to 67 days (±3 days).

An independent DMC will be established by the sponsor or designee to review accumulating study data at regular intervals (as per the DMC charter) throughout the study to ensure the safety of patients and review overall study conduct. The DMC can recommend in writing to the sponsor

This document is confidential.

whether to continue, modify, or stop the clinical study on the basis of safety considerations (see Section 15.8 for further details).

No interim analysis is planned.

7.2 Discussion of Study Design

This study is designed to investigate the efficacy and safety of 300 mg MP1032 BID plus SoC versus placebo plus SoC treatment in hospitalized adults with confirmed moderate to severe COVID-19, the disease caused by SARS-CoV-2 infection.

This study will be executed during a worldwide outbreak of COVID-19 that has resulted in capacity issues at many medical facilities. Additionally, the highly contagious nature of COVID-19 (compounded by a shortage of personal protective equipment) has caused concerns about the safety of healthcare providers caring for these seriously sick patients. Consequently, this study is designed to minimize any additional burden on clinical sites, including minimizing the number of assessments and interventions that the study will require in addition to SoC, while still ensuring the safety of patients.

This study will be double-blinded and placebo-controlled to limit bias in study assessments. Either MP1032 or placebo will be administered as add-on therapy to SoC. Most interventions and assessments may be accomplished in conjunction with SoC, without requiring additional hospital visits or resources.

The dose, dosing regimen, and duration of treatment planned for this study (300 mg MP1032 BID for 28 days) were selected on the basis of:

- The highest repeated dose was 300 mg MP1032 BID applied to both healthy volunteers (7 days) and psoriasis patients (3 months). MP1032 was well tolerated in all studies and safety analyses did not reveal any clinically important safety issues (see the current version of the IB for further information).
- Acute treatment for symptoms of respiratory viral infections is typically from 14 to 28 days.
- A 28-day treatment period was selected because it is a standard duration of time included in the WHO R&D Blueprint for COVID-19 (WHO 2020d).

All patients will be treated for 28 days (unless the study drug is discontinued for safety reasons) to provide a fixed duration of treatment across all patients. As it is impractical to require the study site to continue to hospitalize patients who no longer require hospital care, if the patient is discharged home before Day 28, the study team will provide the patient with the remainder of the assigned blinded study drug kit(s) to take home along with instructions on how to continue treatment at home on the day-of-discharge. Any patient who is discharged during the study should complete the day-of-discharge assessments before release from the hospital as noted in the Schedule of Assessments (Table 1). Patients who progress to the use of an invasive ventilation (mechanical ventilator and/or ECMO) or who can no longer swallow the study drug

(>2 days [ie, 2-day interruption is acceptable]) must discontinue the study drug; but whenever possible, the study assessments will be performed on Day 14, Day 21, Day 28, and Day 60.

This proof-of-concept study will neither include pregnant nor breastfeeding females. In reproduction toxicity studies of MP1032 on fertility and early embryonic as well as on embryo-fetal development, no pathological effects were found. Genotoxicity testing as well as the repeated dose toxicity studies (up to 12 months in dogs and 6 months in rats) did not indicate any toxicological effects. Hence, MP1032 is considered as IMP with unlikely human teratogenicity/fetotoxicity in early pregnancy (see the current version of the IB for more information). However, due to the early clinical stage of development, only women who are neither pregnant nor breastfeeding will be randomized in this study. Women of childbearing potential (WOCBP) and non-sterile male patients with female partners who are WOCBP will be required to use a highly effective method of contraception from the time of first dose of study medication until at least 30 days after the last dose of study medication.

7.3 End of Study

A patient will have fulfilled the requirements for study completion if/when the patient has completed all study periods, including Day 60 as indicated in the Schedule of Assessments (Table 1).

The end of the study will be the last patient's last visit (ie, Day 60) as indicated in the Schedule of Assessments (Table 1).

8 SELECTION OF STUDY POPULATION

Section 7.1 provides information regarding number of patients planned to be randomized.

8.1 Inclusion Criteria

Individuals must meet all of the following criteria to be included in the study:

1. The patient must be willing and able to give informed consent to participate in the study and to adhere to the procedures stated in the protocol or, for adults incapable of consenting due to their medical condition (eg, too weak or debilitated, severe shortness of breath) or due to literacy issues, the patient's legally authorized representative (LAR) must be willing and able to give informed consent on behalf of the patient to participate in the study as permitted by local regulatory authorities, institutional review boards (IRBs)/independent ethics committees (IECs), or local laws.
2. The patient is male or female adult aged ≥18 years (as per local laws) at the time of giving informed consent.
3. The patient is admitted to a hospital and has a positive SARS-CoV-2 test by standard RT-PCR assay or equivalent test. Please note: If the patient has a previous confirmation of SARS-CoV-2 (within 7 days of Day 1), the SARS-CoV-2 test at screening is not required.

4. The patient has the presence of any symptom(s) suggestive of moderate or severe systemic illness with COVID-19 on Day 1, such as presence of fever (≥38.0°C [≥100.4°F] by any route), loss of smell or taste, cough, sore throat, malaise, headache, muscle pain, gastrointestinal symptoms, shortness of breath upon exertion and/or at rest, or respiratory distress.
5. The patient has the presence of moderate to severe clinical signs indicative of moderate or severe illness with COVID-19 on Day 1:
    a. Moderate:
        i. Clinical signs suggestive of moderate illness with COVID-19, such as respiratory rate ≥20 breaths per minute, SpO_2 >93% (on room air at sea level, if possible), heart rate ≥90 beats per minute
        ii. No clinical signs indicative of severe or critical COVID-19
    b. Severe:
        i. Clinical signs suggestive of severe systemic illness with COVID-19, such as respiratory rate ≥30 breaths per minute, heart rate ≥125 beats per minute, SpO_2 ≤93% (on room air at sea level, if possible), PaO_2/FiO_2 <300, or diagnosed with ARDS (according to the Berlin definition; see Table 3)
        ii. No criteria met for critical COVID-19

    Please note: Patients who are receiving oxygen therapy at baseline for a chronic condition (eg, emphysema, COPD, PAH, IPF, etc) must be considered as having severe COVID-19 (unless the patient meets the definition for critical COVID-19). For questions related to COVID-19 severity, the investigator should contact the medical monitor.

6. The patient does not require hemodialysis (chronic) or any renal replacement therapies at screening or Day 1.
7. The patient is able to swallow the study drug (hard gelatin capsules).
8. The patient agrees to minimize strong sun exposure (sunbathing) and strong ultraviolet exposure during the course of the study. Additionally, during the study, patients must agree to use sunscreen when spending an extended period outdoors.
9. Men whose sexual partners are WOCBP must agree to comply with 1 of the following contraception requirements from the time of first dose of study medication (Day 1) until at least 30 days after the last dose of study medication:
    a. Vasectomy with documentation of azoospermia.
    b. Sexual abstinence (defined as refraining from heterosexual intercourse from the time of first dose of study medication until at least 30 days after the last dose of study medication)
    c. Male condom plus partner use of 1 of the contraceptive options below: contraceptive subdermal implant; intrauterine device or intrauterine system; oral contraceptive, either combined or progestogen alone; injectable progestogen; contraceptive vaginal ring; percutaneous contraceptive patches.

The above is an all-inclusive list of those methods that meet the following definition of highly effective: having a failure rate of <1% per year when used consistently and correctly and, when applicable, in accordance with the product label. For non-product methods (eg, male sterility), the investigator will determine what is consistent and correct use. The investigator is responsible for ensuring that patients understand how to properly use these methods of contraception.

10. WOCBP must agree to comply with 1 of the following contraception requirements from the time of first dose of study medication (Day 1) until at least 30 days after the last dose of study medication:
    a. Sexual abstinence (defined as refraining from heterosexual intercourse from the time of first dose of study medication until at least 30 days after the last dose of study medication)
    b. Use of 1 of the contraceptive options below plus use of a condom by male partner: contraceptive subdermal implant; intrauterine device or intrauterine system; oral contraceptive, either combined or progestogen alone; injectable progestogen; contraceptive vaginal ring; percutaneous contraceptive patches.
    c. Vasectomy of male partner with documentation of azoospermia.

    The above is an all-inclusive list of those methods that meet the following definition of highly effective: having a failure rate of <1% per year when used consistently and correctly and, when applicable, in accordance with the product label. The investigator is responsible for ensuring that patients understand how to properly use these methods of contraception. Women of non-reproductive potential are defined as:
    a. Premenopausal females with 1 of the following: documented tubal ligation; documented hysteroscopic tubal occlusion procedure with follow-up confirmation of bilateral tubal occlusion; hysterectomy; documented bilateral oophorectomy.
    b. Postmenopausal defined as 12 months of spontaneous amenorrhea [in questionable cases a blood sample with simultaneous follicle stimulating hormone and estradiol levels consistent with menopause (refer to laboratory reference ranges for confirmatory levels)]. Women on hormone replacement therapy (HRT) and whose menopausal status is in doubt will be required to use 1 of the highly effective contraception methods listed above if they wish to continue their HRT during the study. Otherwise, they must discontinue HRT to allow confirmation of postmenopausal status before randomization.

8.2 Exclusion Criteria

Individuals meeting any of the following criteria are ineligible to participate in this study:

1. The patient, in the opinion of the investigator, is not likely to survive for ≥48 hours beyond Day 1.
2. The patient has a diagnosis of asymptomatic COVID-19, mild COVID-19, or critical COVID-19 on Day 1.

a. Asymptomatic COVID-19 is defined as a patient with a positive SARS-CoV-2 test by standard RT-PCR assay or equivalent test but not experiencing symptoms.
b. Mild COVID-19 is defined as a patient with a positive SARS-CoV-2 test by standard RT-PCR assay or equivalent test and experiencing symptoms of mild illness but no clinical signs indicative of moderate, severe, or critical COVID-19.
c. Critical COVID-19 is defined as a patient with a positive SARS-CoV-2 test by standard RT-PCR assay or equivalent test and experiencing at least 1 of the following: shock defined by systolic blood pressure <90 mm Hg or diastolic blood pressure <60 mm Hg, or requiring vasopressors; respiratory failure requiring endotracheal intubation and invasive mechanical ventilation, oxygen delivered by high-flow nasal cannula (heated, humidified, oxygen delivered via reinforced nasal cannula at flow rates >20 L/min with fraction of delivered oxygen ≥0.5), non-invasive positive pressure ventilation, ECMO, or clinical diagnosis of respiratory failure (ie, clinical need for 1 of the preceding therapies, but preceding therapies not able to be administered in setting of resource limitation), and/or multi-organ dysfunction/failure.

Please note: Patients who are receiving oxygen therapy at baseline for a chronic condition (eg, emphysema, COPD, PAH, IPF, etc) must be considered as having severe COVID-19 (unless the patient meets the definition for critical COVID-19). For questions related to COVID-19 severity, the investigator should contact the medical monitor.

3. The patient has a Child Pugh score ≥ C.
4. The patient has a documented medical history of infection with hepatitis A, B, or C at screening or Day 1.
5. The patient has a documented medical history of infection with human immunodeficiency virus <u>and</u> has a detectable viral load and CD4 count <500 cells/µL.
6. The patient has a documented active infection with tuberculosis at screening or Day 1.
7. The patient has clinically significant electrocardiogram (ECG) abnormalities at screening.
8. A female patient who is pregnant, planning to become pregnant during the study, breastfeeding, or has a positive pregnancy test at screening (by serum) or before dosing on Day 1 (by urine) as determined by human chorionic gonadotrophin (hCG) tests.
9. The patient is planning to donate or bank ova or sperm from Day 1 until 30 days after the last dose of study drug.
10. The patient has a known history of drug or alcohol abuse within 6 months of study start that would interfere with the patient's participation in the study.
11. The patient has a history of sensitivity to any of the study medications, components thereof (eg, mannitol or gelatin), or a history of drug or other allergy that, in the opinion of the investigator or medical monitor, would contraindicate their participation.
12. The patient has participated in and/or plans to participate in another clinical study using an investigational product within the following period before the first dosing day in the

current study: 30 days, 5 half-lives, or twice the duration of the biological effect of the investigational product (whichever is longer).

13. The patient will be transferred to another hospital that is not a study site within 72 hours. Please note: If the investigator has admitting privileges to the transfer hospital, the patient may be considered for randomization.
14. The patient is employed by MetrioPharm, the contract research organization or clinical site involved in the clinical study.
15. The investigator makes a decision that study involvement is not in patient’s best interest, or the patient has any condition or critical illness, in the opinion of the investigator, that will not allow the protocol to be followed safely.

8.3 Rescreening

Individuals who give informed consent to participate in the study but who do not subsequently meet all the requirements as outlined in the inclusion and exclusion criteria and therefore are not randomized (screen failures) may be rescreened. Such individuals may be allowed to rescreen only 1 time.

8.4 Study Withdrawal, Removal, and Replacement of Patients

Patients who terminate the study before Day 28 will be asked to complete the day-of-discharge study procedures. Patients who terminate the study after Day 28 will be asked to complete the Day 60 study procedures. Please note: It is expected that patients who discontinue the study drug early remain in the study, whenever possible, until the completion of the Day 60 study procedures. If it is not possible to follow-up with the patient until Day 60, then follow-up should occur until at least Day 28.

Once a patient is withdrawn from the study, the patient may not return to the study.

A patient may voluntarily withdraw or be withdrawn from the study at any time for the following reasons:

- patient withdrawal of consent (at any time, a patient's participation in the study may be terminated at his/her request)
- lost to follow-up: the patient stopped coming for visits, and study personnel were unable to contact the patient.

Please note: In cases in which the patient is deemed lost to follow-up, the investigator or designee must make every effort to regain contact with the patient (eg, telephone calls, letter to the patient’s last known mailing address, or local equivalent methods). In cases where the patient’s LAR provides informed consent on behalf of the patient, the study site should attempt to contact the LAR. Additionally, the study site should attempt to regain contact with the patient using the alternative contact information provided by the patient/LAR at the time of informed consent. These contact attempts must be documented in the patient’s medical records.

The reason and date for patient withdrawal from the study must be recorded on the electronic case report form (eCRF).

Additionally, the sponsor or DMC may stop the study at any time for safety, regulatory, legal, or other reasons aligned with good clinical practice (GCP). This study may be terminated at the discretion of the sponsor or any regulatory agency. An investigator may elect to terminate or stop the study at his or her study site for any reason, including safety or low recruitment.

9 TREATMENTS

9.1 Details of Study Drugs

Both MP1032 hard gelatin capsules 50 mg and matching placebo capsules will be supplied as 14-day blinded study drug kits. Each kit will also include reserve supply for 1 additional day. Thus, each kit will include 180 capsules in total. IMP will be packaged and labelled according to all applicable local legal requirements. Study drug must be stored in a controlled environment at the study site's pharmacy where storage temperature is monitored, and access is limited to authorized personnel only. In the pharmacy, both MP1032 capsules and matching placebo capsules must be stored in the primary and/or secondary packaging at room temperature (15°C to 25°C [59°F to 77°F]) and protected from light. If the patient is discharged home before Day 28, the study team will provide the patient with the remainder of the assigned blinded study drug kit(s) to take home along with instructions on the day-of-discharge.

Further details of the blinded study drug kits will be described in a Pharmacy Manual.

9.2 Dosage Schedule

All randomized patients will receive study drug from Day 1 to Day 28 according to their assigned treatment and according to the randomization scheme.

Please note: Patients who remain hospitalized after 28 days of consecutive treatment with study drug are not eligible for additional treatments with study drug. Patients will receive 6 capsules in the morning and 6 capsules in the evening (ie, approximately every 12 hours, approximately at the same time every day, and at least 8 hours apart) according to their randomized treatment arm assignment as described below:

- Arm A: MP1032 (300 mg [6 × MP1032 hard gelatin capsules 50 mg] BID) for oral administration
- Arm B: 6 × placebo capsules (ie, matching MP1032 hard gelatin capsules 50 mg) BID for oral administration

In Arm A, the planned total daily dose of MP1032 is 600 mg. In Arm B, the planned total daily dose of MP1032 is 0 mg.

Study drug will be administered to each patient with water (ad libitum) for oral administration.

For all patients (except on Day 1 and Day 7 for patients in the PK subset): Patients should not eat within the 30 minutes before planned study drug administration and within the 30 minutes after study drug dosing, when feasible. However, if a patient has eaten within the 30 minutes before their planned study drug administration, study drug dosing may proceed. The study site should record the date and time of dosing (recorded as the time when the first capsule is administered).

For patients in the PK subset on Day 1 and Day 7 only: Patients should not eat within the 4 hours before planned study drug administration and within the 120 minutes after study drug dosing, when feasible. Thus, study drug should be administered in fasted state (≥ 4 hours), when feasible. However, if a patient has eaten within the 4 hours before their planned administration, study drug dosing may proceed. The patient's fed or fasted state, as well as food intake (yes/no) within the 120 minutes after study drug dosing should be recorded. The PK samples should be collected as planned regardless of the patients' fasted/fed state. The study site should record the date and time of dosing (recorded as the time when the first capsule is administered).

Any missed dose study drug may be administered as soon as possible and the next scheduled dose may be administered according to the planned schedule as long as the doses are at least 6 to 8 hours apart. If study drug administration is interrupted for >2 days, the investigator should notify the medical monitor to determine if the study drug administration can be resumed.

Patients will be asked to abstain from the following products that may potentially affect their safety and/or the PK profile of the study drug:

- soft or hard drugs (including cannabis) from screening and throughout the study
- smoking or using electronic cigarettes while admitted to the hospital
- consumption of alcohol-based products will be prohibited from screening until the final dose of study drug

9.3 Measures to Minimize Bias: Randomization and Blinding

9.3.1 Randomization

The randomization of patients to treatment groups will be performed centrally by an IWRS using a randomization scheme that will be produced by an unblinded, independent statistician. During the randomization process, IWRS will assign a randomization number. Each patient will be assigned one kit number at randomization and a second kit number at the Day 14 visit. The study drug kits will contain the respective blinded treatment available at the study site. Further information on IWRS process will be provided in the IWRS Manual.

Approximately 120 patients will be randomly assigned (2:1) as follows:

- Arm A (300 mg MP1032 BID plus SoC): approximately 80 patients
- Arm B (placebo BID plus SoC): approximately 40 patients

The stratification factor for randomization will include baseline COVID-19 severity (moderate versus severe) and age-class (aged ≤65 years versus >65 years). COVID-19 severity will be determined using the following criteria:

- Moderate COVID-19:
    - Positive SARS-CoV-2 testing by standard RT-PCR assay or equivalent test
    - Symptoms of moderate illness with COVID-19, which could include any symptom of mild illness or shortness of breath with exertion
    - Clinical signs suggestive of moderate illness with COVID-19, such as respiratory rate ≥20 breaths per minute, SpO_2 >93% (on room air at sea level, if possible), heart rate ≥90 beats per minute
    - No clinical signs indicative of severe or critical COVID-19
- Severe COVID-19:
    - Positive SARS-CoV-2 testing by standard RT-PCR assay or equivalent test
    - Symptoms suggestive of severe systemic illness with COVID-19, which could include any symptom of moderate illness or shortness of breath at rest, or respiratory distress
    - Clinical signs indicative of severe systemic illness with COVID-19, such as respiratory rate ≥30 breaths per minute, heart rate ≥125 beats per minute, SpO_2 ≤93% (on room air at sea level, if possible), PaO_2/FiO_2 <300, or diagnosed with ARDS (according to the Berlin definition; see Table 3)
    - No criteria met for critical COVID-19

To standardize the assessment of COVID-19 severity, respiratory rate, SpO_2, and heart rate will be measured when the patient is on room air at sea level (ie, no supplemental oxygen, if possible) and at rest for at least 5 minutes. If possible, the site should collect the information from each patient at the same time each day (±1 hour).

Please note: Patients who are receiving oxygen therapy at baseline for a chronic condition (eg, emphysema, COPD, PAH, IPF, etc) must be considered as having severe COVID-19 (unless the patient meets the definition for critical COVID-19). For questions related to COVID-19 severity, the investigator should contact the medical monitor.

9.3.2 Blinding

The study is double-blinded. Study drug will be identical in appearance and labeled in a blinded manner. All patients (and their LARs), investigators, and all study staff involved in the conduct of the study (including sponsor personnel or sponsor designees), including data management and biostatistics, will be blinded to treatment assignment.

Exception: An unblinded, independent statistician will be assigned to produce the randomization schedule and unblinded tables, figures, and listings for the DMC. The unblinded statistician will not otherwise participate in study procedures.

In the event that emergency unblinding is required for a given patient because of AEs or concerns for the patient's safety or well-being, the investigator may break the randomization code for the patient via the IWRS, by which system the unblinding will be captured. The investigator is responsible for notifying the medical monitor and/or sponsor of such an event as soon as possible. The unblinding and its cause will also be documented in the eCRF.

If an investigator becomes unblinded to a given patient's study drug, that patient will be discontinued from the study drug unless there are ethical reasons for that patient not to be discontinued; approval from the sponsor's medical monitor must be obtained in such instances.

9.4 Treatment Modifications and Discontinuations

Dose reductions and adjustments are not permitted; any dose reductions and adjustments that occur will be recorded and considered protocol deviations.

Delays and omissions of study drug administration because of medical or non-medical reasons are allowed. All delays and omissions of study drug that occur during the study will be recorded and considered protocol deviations.

All patients will be treated for 28 days unless the study drug is discontinued for safety reasons, or the patient withdraws consent. If a patient is discontinued from the study drug for any reason, the study site must immediately notify the medical monitor.

A patient may voluntarily discontinue the study drug or be discontinued from the study drug at any time for reasons including, but not limited to, the following:

- progressive disease (related to COVID-19). Please note: Patients who progress to the use of an invasive ventilation (mechanical ventilator and/or ECMO) or who can no longer swallow the study drug (>2 days [ie, 2-day interruption is acceptable]) must discontinue the study drug; but whenever possible, the study assessments will be performed on Day 14, Day 21, Day 28, and Day 60.
- unacceptable AE and/or SAE
- patient withdrawal of consent (at any time, a patient's participation in the study may be terminated at his/her request)
- on the basis of the investigator's clinical judgment
- general or specific changes in the patient's condition that render him/her ineligible for further treatment according to the inclusion/exclusion criteria
- patient fails to adhere to the protocol requirements (eg, drug noncompliance)
- lost to follow-up: the patient stopped coming for visits, and study personnel were unable to contact the patient.

  Please note: In cases in which the patient is deemed lost to follow-up, the investigator or designee must make every effort to regain contact with the patient (eg, telephone calls,

letter to the patient's last known mailing address, or local equivalent methods). In cases where the patient's LAR provides informed consent on behalf of the patient, the study site should attempt to contact the LAR. Additionally, the study site should attempt to regain contact with the patient using the alternative contact information provided by the patient/LAR at the time of informed consent. These contact attempts must be documented in the patient's medical records.

- pregnancy, as indicated in Section 12.6.5.

The reason and date for the patient's discontinuation from study drug must be recorded on the eCRF.

Please note: It is expected that patients who discontinue study drug early remain in the study, whenever possible, until the completion of the Day 60 study procedures. If it is not possible to follow-up with the patient until Day 60, then follow-up should occur until at least Day 28.

9.5 Treatment Accountability and Compliance

The pharmacist or other designated individual will maintain records of study drug delivered to the study site, the inventory at the study site, the distribution to and use by each patient, and the return of materials to the sponsor for storage or disposal. These records should include dates, quantities, batch/serial numbers, expiration dates, in-clinic temperature log, and unique code numbers assigned to the product and patients.

During hospitalization, the administration of the study drug will be performed by the investigator (or qualified blinded designee) to ensure compliance. If the patient is discharged home before Day 28, the study team will provide the patient with the remainder of the assigned blinded study drug kit(s) to take home along with instructions to continue treatment at home; for these patients, treatment compliance will be conducted via a diary. If the patient does not feel comfortable to complete the diary, the site will alternatively provide the patient with the option to be called, twice a day, to confirm that the study drug is taken as instructed. Patients are to be reminded of the importance of compliance with their assigned regimen, with an emphasis on taking their study drug on schedule and maintaining the prescribed interval between doses.

Investigators will maintain records that adequately document that the patients were provided with the correct study drug kit(s) and reconcile the products received from the drug dispensing center. Investigational product will not be returned to the sponsor until accountability has been fully monitored.

Noncompliance is defined as taking <80% or >120% of study drug. Discontinuation for noncompliance is at the investigator's discretion and is to be noted on the eCRF.

9.6 Prior and Concomitant Medications

Restricted prior medications are provided in Section 8.2.

All medications and other treatments taken by the patient during the study, including those treatments initiated before the start of the study, must be recorded on the eCRF.

Medications taken by or administered to the patient for the period before screening will be recorded in the eCRF. After the screening visit, medications are generally permitted; however, the following therapies are expressly prohibited during the screening and treatment periods and up through the end of the follow-up period (ie, up through Day 28):

- an investigational product from another clinical study

Any medication or therapy that is taken by or administered to the patient during the course of the study must be recorded in the eCRF. The entry must include the dose, regimen, route, indication, and dates of use.

10 STUDY PROCEDURES

Table 1 outlines the timing of procedures and assessments to be performed throughout the study. Section 12.5 specifies clinical laboratory assessment samples to be obtained. See Section 11, Section 12, Section 13, and Section 14 for additional details regarding efficacy, safety, PK, other assessments, respectively.

Table 1: Schedule of Assessments

Study Period	Screening Period		Treatment Period				Follow-up Period	-
**Visit Name**	**Screening[a]**	**Day 1[a]**	**Days 2 to 13**	**Day 14[b]**	**Day 21[b]**	**Day 28 (End of Treatment Visit)[b]**	**Day 60 (Follow-up Visit)[b] / Early Study Termination after Day 28**	**Day of Discharge / Early Study Termination before Day 28**
**Study Day (window)**	**Day -7 to Day -1**	**Day 1 (NA)**	**Days 2 to 13 (NA)**	**Day 14 (±2 days)**	**Day 21 (±2 days)**	**Day 28 (±3 days)**	**Day 60 (±3 days)**	**Day of Discharge**
Informed consent[c]	X	-	-	-		-	-	-
COVID-19 testing[d]	X	X	-	X[e]		X[e]	X[e]	-
Demographics[f]	X	-	-	-		-	-	-
Medical/surgical history[g]	X	X (updates only)	-	-		-	-	-
Inclusion/exclusion criteria	X	X	-	-		-	-	-
Complete physical examination[h]	X	-	-	-		-	-	-
Limited physical examination[h]	-	X	X (only Day 8 [±3 days])[s]	X[e]		X[e]	-	X
Vital signs[i]	X	X	X[j]	X[e]		X[e]	X[e]	X
Height	X	-	-	-		-	-	-
Weight and BMI	X	X	-	X[e]		X[e]	X[e]	X
Clinical laboratory assessments (hematology, blood biochemistry, coagulation, and urinalysis)	X	X	X (only Day 8 [±3 days])[s]	X (Days 14, 21, and 28)[e, u]			-	X
ECG[k]	X	-	-	-		-	-	-
Pregnancy test (WOCBP only)[l]	X	X	-	X		-	-	X (only if discharged before Day 14 or at Early Study

**Study Period**	**Screening Period**		**Treatment Period**				**Follow-up Period**	**-**
**Visit Name**	**Screening[a]**	**Day 1[a]**	**Days 2 to 13**	**Day 14[b]**	**Day 21[b]**	**Day 28 (End of Treatment Visit)[b]**	**Day 60 (Follow-up Visit)[b] / Early Study Termination after Day 28**	**Day of Discharge / Early Study Termination before Day 28**
**Study Day (window)**	**Day -7 to Day -1**	**Day 1 (NA)**	**Days 2 to 13 (NA)**	**Day 14 (±2 days)**	**Day 21 (±2 days)**	**Day 28 (±3 days)**	**Day 60 (±3 days)**	**Day of Discharge**
								Termination before Day 14)
Randomization via IWRS	-	X	-	-		-	-	-
Retrieve the 14-day blinded study drug kit via the study drug kit number assigned by IWRS		X		X[v]				X (only if discharged before Day 14; NA for Early Study Termination)
Blood sample collection for biomarkers	-	X[o]	X (only Day 7)[o]	X (Days 14, 21, and 28)[o]			X[e,o]	-
Administer blinded study drug BID	-		X[t]				-	-
Provide remainder of the assigned blinded study drug kit(s) to the patient along with instructions[m]	-	-	-	-		-	-	X (only if discharged during the treatment period; NA for Early Study Termination)
Provide patient with the diary and train patient on use of the diary[m]	-	-	-	-		-	-	X (only if discharged during the treatment period; NA for Early Study Termination)
COVID-19 symptoms	X	X	-	X		X	X	X
COVID-19 severity[p]	X	X	-	-		-	-	-
Clinical status related to COVID-19 on the NIAID 8-point ordinal scale[q]	X	X	X	X		X	X	X

Study Period	Screening Period		Treatment Period				Follow-up Period	-
Visit Name	Screening[a]	Day 1[a]	Days 2 to 13	Day 14[b]	Day 21[b]	Day 28 (End of Treatment Visit)[b]	Day 60 (Follow-up Visit)[b] / Early Study Termination after Day 28	Day of Discharge / Early Study Termination before Day 28
Study Day (window)	Day -7 to Day -1	Day 1 (NA)	Days 2 to 13 (NA)	Day 14 (±2 days)	Day 21 (±2 days)	Day 28 (±3 days)	Day 60 (±3 days)	Day of Discharge
EQ-5D-5L questionnaire[r]	-	-	-	-		-	X	X (only if discharged/ early terminated before Day 60)
Blood sample collection (plasma) for PK (from the PK subset of approximately 30 patients who give optional consent)[n]	-	X	X (only Day 7)[j]			-	-	-
AEs	X							X
Prior and concomitant medications	X							X

Abbreviations: AE = adverse event; BID = twice daily; BMI = body mass index; COVID-19 = coronavirus 2019; ECG = electrocardiogram; eCRF = electronic case report form; EQ-5D-5L = EuroQol-5D-5L; IEC = independent ethics committee; ICU = intensive care unit; IRB = institutional review board; IWRS = interactive web-response system; LAR = legally authorized representative; NA = not applicable; NIAID = National Institute of Allergy and Infectious Diseases; PK = pharmacokinetic; RT-PCR = reverse transcription-polymerase chain reaction; SAE = serious adverse event; SARS-CoV-2 = severe acute respiratory syndrome coronavirus type 2; SoC = standard of care; SpO_2 = saturation of oxygen; WOCBP = women of childbearing potential

a. Those patients who are fully eligible (all screening assessments are available) can have Day 1 performed on the same day as screening. If screening and Day 1 are performed on the same day, visit procedures required for both visits do not need to be repeated.
b. Patients will be assessed while hospitalized. If patients are discharged from the hospital, they can have the study visits at Day 14, Day 21, Day 28, and Day 60 as an outpatient visit. Patients discharged early will continue to receive study drug through Day 28. For discharged patients, Day 14 and Day 28 visits are preferred to be conducted in person at the study site. If restrictions limit the ability of the patient to return to the study site, these visits may be conducted by home healthcare visit or by telephone/virtual call (the patient's caregiver may assist during the telephone/virtual call). For discharged patients, it is sufficient to conduct the Day 60 Follow-up Visit as a telephone/virtual call (the patient's caregiver may assist during the telephone/virtual call) or an outpatient or home healthcare visit is also allowed. Please note: In cases in which the patient is deemed lost to follow-up, the investigator or designee must make every effort to regain contact with the patient (eg, telephone calls, letter to the patient's last known mailing address, or local equivalent methods). In cases where the patient's LAR provides informed consent on behalf of the patient, the study site should attempt to contact the LAR. Additionally, the study site should attempt to regain contact with the patient using the alternative contact information provided by the patient/LAR at the time of informed consent. These contact attempts must be documented in the patient's medical records.

c. The patient must give informed consent to participate in the study or, for adults incapable of consenting due to their medical condition (eg, too weak or debilitated, severe shortness of breath) or due to literacy issues, the patient's LAR must be willing give informed consent on behalf of the patient to participate in the study as permitted by local regulatory authorities, IRBs/IECs, or local laws.

d. At the screening visit, the local laboratory may perform COVID-19 testing by standard RT-PCR assay or equivalent test. At screening, a positive test must be available in order for the patient to be randomized on Day 1. If the patient has a previous confirmation of SARS-CoV-2 (within 7 days of Day 1), the SARS-CoV-2 test at screening is not required. On Day 1, Day 14, Day 28, and Day 60, samples will be sent to the central laboratory for COVID-19 testing by standard RT-PCR assay. The result of the COVID-19 tests (positive/negative) must be documented in the eCRF. Further details will be described in the laboratory manual. Please note: If COVID-19 tests are performed at unscheduled time points (eg, according to SoC), findings (ie, positive/negative result) must be recorded in the eCRF.

e. Not all assessments will be able to be performed if the Day 14, Day 28, and/or 60 visits are conducted via a telephone/virtual call (the patient's caregiver may assist during the telephone/virtual call). If the assessment can be conducted via a telephone/virtual call, the study site should record the details; otherwise, the missed assessment will not be considered a protocol deviation. If the visit is conducted by a home healthcare nurse, these assessments should be conducted, if possible; if the assessment can be conducted via home healthcare nurse, the study site should record the details; otherwise, the missed assessment will not be considered a protocol deviation. These assessments must be conducted if the visit is conducted at the study site. All efforts should be made to collect clinical laboratory assessments on Day 14 (± 2 days) and Day 28 (± 3 days). Please note: In cases in which the patient is deemed lost to follow-up, the investigator or designee must make every effort to regain contact with the patient (eg, telephone calls, letter to the patient's last known mailing address, or local equivalent methods). In cases where the patient's LAR provides informed consent on behalf of the patient, the study site should attempt to contact the LAR. Additionally, the study site should attempt to regain contact with the patient using the alternative contact information provided by the patient/LAR at the time of informed consent. These contact attempts must be documented in the patient's medical records.

f. Demographics will include sex, age, and race and/or ethnicity.

g. Medical/surgical medical history includes all active and chronic diseases (eg, asthma and COPD), and any diseases diagnosed in the past 1 year (before screening) that the investigator considers clinically significant. Additional pre-existing conditions present at the time when informed consent is given and up to the time of first dosing (Day 1) are to be regarded as concomitant. Medical history will include documentation of social behaviors, including use of tobacco, nicotine (including use of electronic cigarettes), and soft or hard drugs (including cannabis), as well as alcohol consumption, if applicable. Smoking history also must be documented as part of medical history and includes never, light (<100 cigarettes/lifetime), active smoker, and former smoker (quit date). History of number packs/day, number of smoking years, and quitting time for former and active smokers must also be documented. Additionally, the investigators will document at screening the patient's Charlson score via the Charlson Comorbidity Index (see Section 12.1 for further details) (Appendix 1).

h. A complete physical examination will be performed at screening. Physical examinations will be performed by a physician, nurse, or other appropriately trained staff. The complete physical examination includes head, eyes, ears, nose, and throat; heart; lungs; abdomen; skin; cervical and axillary lymph nodes; and neurological and musculoskeletal systems. A limited physical examination to verify continued patient eligibility and to follow-up regarding any change in medical history will be performed at the visits indicated above. Symptom-driven, limited physical examinations may be performed as clinically indicated during the study (according to SoC).

i. Vital signs include systolic and diastolic blood pressure, heart rate, respiration rate, SpO_2, and body temperature. All vital signs will be measured after the patient has been resting for at least 5 minutes. Blood pressure measurements are to be taken in the same arm for the duration of the study. Moreover, additional vital signs may be performed as needed in case of any cardiac AE.

j. For patients who remain hospitalized only.

k. A 12-lead, resting ECG will be obtained locally at screening. At screening, the investigator must examine the ECG traces for clinically significant abnormalities that could exclude the patient from the study. ECGs should always be obtained in supine position after adequate rest (≥5 minutes).

l. For WOCBP, a serum sample for pregnancy testing must be collected at screening and a urine sample for pregnancy testing must be collected before the first dose on Day 1. Pregnancy tests must be negative for the patient to be randomized and treated. A urine pregnancy test will be repeated on Day 14 (± 7 days). If the patient is discharged before Day 14, urine pregnancy test at day of discharge should be done, and urine pregnancy test at Day 14 is not required. If the patient is discharged after Day 14, urine pregnancy test should be done at Day 14, and urine pregnancy test at day of discharge is not required (whichever comes first). Patients with a positive pregnancy test will be discontinued from study drug.

This document is confidential.

m. Only for patients discharged before Day 28. If the patient does not feel comfortable to complete the diary, the site will alternatively provide the patient with the option to be called, twice a day, to confirm that the study drug is taken as instructed.

n. Plasma PK samples will be collected via sparse sampling from approximately 30 patients (who give optional consent) in a 2:1 ratio to assess the PK of MP1032 on Day 1 (single dose) and Day 7 (steady state). A total of 16 samples will be collected relative to the first dose on Day 1 and the first dose on Day 7. Samples on Day 1 and Day 7 will be collected predose (within 10 minutes before the first daily dose) and postdose at 10 minutes, 20 minutes, 30 minutes, 60 minutes, 120 minutes, 8 hours (before next dose), and 24 hours before the next morning dose (if applicable), if possible. Patients who provide consent for the PK sampling, but are no longer hospitalized on Day 7, will not have Day 7 PK samples collected.

o. Blood samples to be obtained on Day 1 (before the first study drug administration) and Days 7, 14, 21, 28, and 60 (when possible). Blood samples for biomarkers must be collected on Days 7, 14, 21, 28, and 60 if a patient is still hospitalized. If a patient is discharged, the study site should arrange an outpatient visit (ie, at the study site) to accommodate the collection of blood samples for biomarkers on Days 7, 14, 21, 28, and 60.

p. To standardize the assessment of COVID-19 severity, respiratory rate, SpO_2, and heart rate will be measured when the patient is on room air at sea level (ie, no supplemental oxygen, if possible) and at rest for at least 5 minutes. If possible, the site should collect the information from each patient at the same time each day (±1 hour).

q. Record the patient's clinical status related to COVID-19 on the NIAID 8-point ordinal scale and also record the date and time for each individual component of the NIAID 8-point ordinal scale including: invasive or non-invasive mechanical ventilation/ECMO start/stop, high-flow/mask oxygen start/stop, supplemental oxygen start/stop (nasal cannula, liter flow [and its conversion to FiO_2], other delivery services), hospital discharge (whether with limitations or without limitations), hospitalization type (eg, transfer to ICU) start/stop, and (if applicable) death, cause of death (including relatedness to COVID-19), and date/time of death. If possible, the site should collect the information from each patient at the same time each day (±1 hour).

r. The EQ-5D-5L (Appendix 2) is only required for discharged patients and at the following visits: day of discharge, Day 60, and Early Termination visit (if applicable). The EQ-5D-5L is not required for patients who remain hospitalized until study end.

s. The limited physical examination and clinical laboratory assessments on Day 8 (± 3 days) will be collected in all patients who remain hospitalized. If the patient is discharged before Day 8 (± 3 days), the site should arrange to have the physician assess the patient via a telephone/virtual call. If the physician determines during the telephone/virtual call that the limited physical examination and clinical laboratory assessments are clinically indicated, then the site should arrange for an outpatient visit to collect these assessments.

t. For all patients (except on Day 1 and Day 7 for patients in the PK subset): Patients should not eat within the 30 minutes before planned study drug administration and within the 30 minutes after study drug dosing, when feasible. However, if a patient has eaten within the 30 minutes before their planned study drug administration, study drug dosing may proceed. The study site should record the date and time of dosing (recorded as the time when the first capsule is administered). For patients in the PK subset on Day 1 and Day 7 only: Patients should not eat within the 4 hours before planned study drug administration and within the 120 minutes after study drug dosing, when feasible. Thus, study drug should be administered in fasted state (≥ 4 hours), when feasible. However, if a patient has eaten within the 4 hours before their planned administration, study drug dosing may proceed. The patient's fed or fasted state, as well as food intake (yes/no) within the 120 minutes after study drug dosing should be recorded. The PK samples should be collected as planned regardless of the patients' fasted/fed state. The study site should record the date and time of dosing (recorded as the time when the first capsule is administered).

u. The clinical laboratory assessments on Day 21 will be collected in all patients who remain hospitalized. If the patient is discharged before Day 21, the site should arrange to have the physician assess the patient via a telephone/virtual call. If the physician determines during the telephone/virtual call that the clinical laboratory assessments are clinically indicated, then the site should arrange for an outpatient visit to collect this assessment.

v. The +2-day window does not apply to the retrieval of the 14-day blinded study drug kit as the second kit must be distributed at the latest on Day 14.

This document is confidential.

10.1 Informed Consent

Before performing any study-related procedures, the investigator (or designee) will obtain informed consent from the patient to participate in the study or, for adults incapable of consenting due to their medical condition (eg, too weak or debilitated, severe shortness of breath) or due to literacy issues, the patient's LAR must be willing give informed consent on behalf of the patient to participate in the study as permitted by local regulatory authorities, IRBs/IECs, or local laws.

Please note: The patient/LAR will be expected to provide the study site with their contact information so that the site can maintain contact with the patient/LAR in the event that the patient is discharged before the end of the study. Additionally, at the time of informed consent, the study site may also request for the patient/LAR to provide alternative contact information. In the event that rescreening occurs, the investigator (or designee) must reconsent the patient (or the patient's LAR) and the patient must be assigned a new identification number.

10.2 Study Procedures

Assessments and their timing are to be performed as outlined in the Schedule of Assessments (Table 1). Section 12.5 specifies clinical laboratory assessment samples to be obtained.

Assessments and procedures scheduled at a visit where study drug is administered should be performed before administration of treatment unless otherwise indicated in the Schedule of Assessments (Table 1).

Efficacy assessments are described in Section 11 and include COVID-19 symptoms, COVID-19 severity, clinical status related to COVID-19 on the NIAID 8-point ordinal scale, HRQoL assessed by the EQ-5D-5L questionnaire (Appendix 2), and COVID-19 testing.

Safety assessments are described in Section 12 and include medical/surgical history and demographics, vital signs, physical examinations, ECGs, clinical laboratory assessments, and AEs. PK assessments are described in Section 13 and include PK sampling and PK analytical methodology. Biomarkers are described in Section 14.1.

The investigator may, at his/her discretion, arrange for a patient to have an unscheduled assessment, especially in the case of AEs that require follow-up or are considered by the investigator to be possibly related to the use of study drug. The unscheduled visit page in the eCRF must be completed.

Procedures for early study withdrawal are described in Section 8.4.

11 EFFICACY ASSESSMENTS

The Schedule of Assessments (Table 1) outlines the efficacy assessments to be performed throughout the study and their timing.

11.1 COVID-19 Symptoms

The assessment of the COVID-19 symptoms will be recorded at the visits specified in the Schedule of Assessments (Table 1).

The patient will be assessed for the following as shown in Table 2.

Table 2 COVID-19 Symptoms

Symptom	Response Options and Scoring
1. Stuffy or runny nose	None = 0<br>Mild = 1<br>Moderate = 2<br>Severe =3
2. Sore throat	
3. Red or irritated eyes (conjunctivitis)	
4. Shortness of breath (difficulty breathing)	
5. Cough	
6. Low energy or tiredness	
7. Muscle or body aches	
8. Headache	
9. Chills or shivering	
10. Feeling hot or feverish	
11. Nausea (feeling like you wanted to throw up)	
12. How many times did you vomit (throw up) in the last 24 hours?	I did not vomit at all = 0<br>1–2 times = 1<br>3–4 times = 2<br>5 or more times = 3
13. How many times did you have diarrhea (loose or watery stools) in the last 24 hours?	I did not have diarrhea at all = 0<br>1–2 times = 1<br>3–4 times = 2<br>5 or more times = 3
14. Rate your sense of smell in the last 24 hours.	My sense of smell is THE SAME AS usual = 0<br>My sense of smell is LESS THAN usual = 1<br>I have NO sense of smell = 2
15. Rate your sense of taste in the last 24 hours.	My sense of taste is THE SAME AS usual = 0<br>My sense of taste is LESS THAN usual = 1<br>I have NO sense of taste = 2

Note: Score values are included in the table for ease of reference. Score values within the response options will not be presented to patients to avoid confusing patients.

11.2 COVID-19 Severity

The assessment of the COVID-19 severity will be recorded at the visits specified in the Schedule of Assessments (Table 1). As per the inclusion and exclusion criteria (see Section 8.1 and Section 8.2), patients who are randomized must have either moderate or severe COVID-19. The criteria (FDA 2020) used to determine the patient's COVID-19 severity incorporates objective measures as follows:

This document is confidential.

Asymptomatic SARS-CoV-2 infection

- Positive SARS-CoV-2 testing by standard RT-PCR assay or equivalent test
- No symptoms

Mild COVID-19

- Positive SARS-CoV-2 testing by standard RT-PCR assay or equivalent test
- Symptoms of mild illness with COVID-19 (eg, loss of smell or taste, fever, cough, sore throat, malaise, headache, muscle pain, gastrointestinal symptoms, and without shortness of breath or dyspnea)
- No clinical signs indicative of moderate, severe, or critical COVID-19

Moderate COVID-19

- Positive SARS-CoV-2 testing by standard RT-PCR assay or equivalent test
- Symptoms of moderate illness with COVID-19, which could include any symptom of mild illness or shortness of breath with exertion
- Clinical signs suggestive of moderate illness with COVID-19, such as respiratory rate ≥20 breaths per minute, SpO_2 >93% (on room air at sea level, if possible), heart rate ≥90 beats per minute
- No clinical signs indicative of severe or critical COVID-19

Severe COVID-19

- Positive SARS-CoV-2 testing by standard RT-PCR assay or equivalent test
- Symptoms suggestive of severe systemic illness with COVID-19, which could include any symptom of moderate illness or shortness of breath at rest, or respiratory distress
- Clinical signs indicative of severe systemic illness with COVID-19, such as respiratory rate ≥30 breaths per minute, heart rate ≥125 beats per minute, SpO_2 ≤93% (on room air at sea level, if possible), PaO_2/FiO_2 <300, or diagnosed with ARDS (according to the Berlin definition; see Table 3 for further details)
- No criteria for critical COVID-19

Critical COVID-19

- Positive SARS-CoV-2 testing by standard RT-PCR assay or equivalent test
- Evidence of critical illness, defined by at least 1 of the following:
    - Respiratory failure defined based on resource utilization requiring at least 1 of the following:
        - Endotracheal intubation and mechanical ventilation, oxygen delivered by high-flow nasal cannula (heated, humidified, oxygen delivered via reinforced nasal cannula at flow rates >20 L/min with fraction of delivered oxygen ≥0.5), non-invasive positive pressure ventilation, ECMO, or clinical diagnosis of respiratory failure (ie, clinical need for 1 of the

preceding therapies, but preceding therapies not able to be administered in setting of resource limitation)

- Shock (defined by systolic blood pressure <90 mm Hg, or diastolic blood pressure <60 mm Hg or requiring vasopressors)
- Multi-organ dysfunction/failure

A clinical diagnosis of respiratory failure (in the setting of resource limitation) in which the management deviates from SoC must be recorded.

To standardize the assessment of COVID-19 severity, respiratory rate, SpO_2, and heart rate will be measured when the patient is on room air at sea level (ie, no supplemental oxygen, if possible) and at rest for at least 5 minutes. If possible, the site should collect the information from each patient at the same time each day (±1 hour).

Please note: Patients who are receiving oxygen therapy at baseline for a chronic condition (eg, emphysema, COPD, PAH, IPF, etc) must be considered as having severe COVID-19 (unless the patient meets the definition for critical COVID-19). For questions related to COVID-19 severity, the investigator should contact the medical monitor.

Table 3: Acute Respiratory Distress Syndrome – Berlin Definition

Timing	Within 1 week of known clinical insult or new or worsening respiratory symptoms	
Chest imaging[a]	Bilateral opacities — not fully explained by effusions, lobar/lung collapse, or nodules	
Origin of edema	Respiratory failure not fully explained by cardiac failure or fluid overload. Need objective assessment (eg, echocardiography) to exclude hydrostatic edema if no risk factor is present	
Oxygenation[b]	Mild	200 mm Hg < PaO_2/FIO_2 ≤300 mm Hg with PEEP or CPAP ≥5 cmH_2O[c]
	Moderate	100 mm Hg < PaO_2/FIO_2 ≤200 mm Hg with PEEP ≥5 cmH_2O
	Severe	PaO_2/FIO_2 ≤100 mm Hg with PEEP ≥5 cmH_2O

Abbreviations: ARDS = acute respiratory distress syndrome; CPAP = continuous positive airway pressure; FIO_2 = fraction of inspired oxygen; PaO_2 = partial pressure of arterial oxygen; PEEP = positive end-expiratory pressure.

a Chest radiograph or computed tomography scan

b If altitude is higher than 1,000 m, the correction factor will be calculated as follows: [PaO_2/FIO_2 (barometric pressure/760)]

c This may be delivered noninvasively in the mild ARDS group

Source: Adapted from Fanelli et al 2013

11.3 Clinical Status Related to COVID-19 on the National Institute of Allergy and Infectious Diseases 8-Point Ordinal Scale

The NIAID 8-point ordinal scale is an assessment of the clinical status at the first assessment of a given study day. The scale is as follows:

1. Death

2. Hospitalized, on invasive ventilation (mechanical ventilator and/or ECMO)
3. Hospitalized, on non-invasive ventilation or high-flow oxygen devices
4. Hospitalized, requiring supplemental oxygen
5. Hospitalized, not requiring supplemental oxygen but requiring ongoing medical care (COVID-19 related or otherwise)
6. Hospitalized, not requiring supplemental oxygen and no longer requires ongoing medical care (used if hospitalization was extended for infection-control reasons)
7. Not hospitalized, limitation on activities, and/or requiring home oxygen
8. Not hospitalized, no limitations on activities

Please note: Patients requiring oxygen before COVID-19 and returning to baseline oxygen use will be considered improved (ie, not requiring supplemental oxygen). Patients with a limitation on activities before COVID-19 and returning to baseline activity will be considered improved.

At the visits specified in the Schedule of Assessments (Table 1), record the date and time for each individual component of the NIAID 8-point ordinal scale including: invasive or non-invasive mechanical ventilation/ECMO start/stop, high-flow/mask oxygen start/stop, supplemental oxygen start/stop (nasal cannula, liter flow [and its conversion to FiO_2], other delivery services), hospital discharge (whether with limitations or without limitations), hospitalization type (eg, transfer to ICU) start/stop, and (if applicable) death, cause of death (including relatedness to COVID-19), and date/time of death. If possible, the site should collect the information from each patient at the same time each day (±1 hour).

A standard conversion for liter flow oxygen to FiO_2 is presented in Table 4.

Table 4 Standard Conversion for Liter Flow Oxygen

	**FiO_2 (%)**
Room air	21
1 L/min	24
2 L/min	27
3 L/min	30
4 L/min	33
5 L/min	36
6 L/min	39

If there is use of a venturi mask (air-entrainment mask), the FiO_2 is based on the nozzle use and should also be recorded.

11.4 Health-related Quality of Life Assessment

The HRQoL will be assessed via the EQ-5D-5L questionnaire (Appendix 2). The EQ-5D-5L is a standardized questionnaire developed by the EuroQol Group as a measure of HRQoL that consists of a descriptive system and a VAS. The descriptive system consists of 5 dimensions: mobility, self-care, usual activities, pain/discomfort, and anxiety/depression. The VAS records the patient's self-rated health on a vertical VAS. This can be used as a quantitative measure of health outcome that reflects the patient's own judgment. The patient is asked to indicate his/her current health state (ie, describe their health "today"). The scores on these 5 dimensions can be presented as a health profile or can be converted to a single summary index number that describes the patient's current health state. The EQ-5D-5L questionnaire has been validated in a diverse patient population in 6 countries and is available in multiple languages. The EQ-5D-5L questionnaire is suitable for completion by adults (EuroQol Research Foundation 2020).

The results of the EQ-5D-5L questionnaire will be recorded at the visits specified in the Schedule of Assessments (Table 1). Please note: This assessment will only be performed in patients who have been discharged from the hospital before Day 60.

11.5 COVID-19 Testing

Samples for COVID-19 testing will be obtained at the visits specified in the Schedule of Assessments (Table 1). At the screening visit, the local laboratory may perform COVID-19 testing by standard RT-PCR assay or equivalent test. At screening, a positive test must be available in order for the patient to be randomized on Day 1. If the patient has a previous confirmation of SARS-CoV-2 (within 7 days of Day 1), the SARS-CoV-2 test at screening is not required. The result of the COVID-19 tests (positive/negative) must be documented in the eCRF

On Day 1, Day 14, Day 28, and Day 60, samples will be sent to the central laboratory for COVID-19 testing by standard RT-PCR assay. The result of the COVID-19 tests (positive/negative) must be documented in the eCRF. Further details will be described in the laboratory manual.

Please note: If COVID-19 tests are performed at unscheduled time points (eg, according to SoC), findings (ie, positive/negative result) must be recorded in the eCRF.

12 SAFETY ASSESSMENTS

Safety assessments (medical/surgery history, demographics, prior and concomitant medications, vital signs, physical examinations, ECGs, clinical laboratory assessments, and AEs) are to be performed at the visits specified in the Schedule of Assessments (Table 1).

12.1 Medical/Surgical History and Demographics

Medical/surgical history will be recorded at screening. Investigators will document all active and chronic diseases (eg, asthma and COPD), and any diseases diagnosed in the past 1 year (before

This document is confidential.

screening) that the investigator considers clinically significant. Additional pre-existing conditions present at the time when informed consent is given and up to the time of first dosing (Day 1) are to be regarded as concomitant. Medical history will include documentation of social behaviors, including use of tobacco, nicotine (including use of electronic cigarettes), and soft or hard drugs (including cannabis), as well as alcohol consumption, if applicable. Smoking history also must be documented as part of medical history and includes never, light (<100 cigarettes/lifetime), active smoker, and former smoker (quit date). History of number packs/day, number of smoking years, and quitting time for former and active smokers must also be documented.

Additionally, the investigators will document at screening the patient's Charlson score via the Charlson Comorbidity Index (Charlson et al 1987). The Charlson Comorbidity Index may be used to identify patients with higher risk of COVID-19 progression (Garibaldi et al 2020) via the COVID-19 Inpatient Risk Calculator (online calculator available via: https://rsconnect.biostat.jhsph.edu/covid_predict/). The 14-item Charlson score is based on the patient's comorbidities that are associated with mortality : myocardial infarction, congestive heart failure, peripheral vascular disease, cerebrovascular disease, dementia, chronic pulmonary disease, rheumatologic disease, peptic ulcer disease, liver disease, diabetes, hemiplegia or paraplegia, renal disease, malignancy, and acquired immunodeficiency syndrome/human immunodeficiency virus (online calculator available via: https://www.orthotoolkit.com/charlson-comorbidity-index/). The investigator should record each individual component of the 14-item Charlson Comorbidity Index (Appendix 1) as well as the overall score.

Illnesses first occurring or detected during the study and/or worsening of a concomitant illness during the study are to be documented as AEs on the eCRF in accordance with Section 12.6. All changes not present at baseline or described in the past medical history and identified as clinically noteworthy must be recorded as AEs.

Additionally, demographic data will be collected for all patients and include sex, age, and race and/or ethnicity.

12.2 Vital Signs

Vital signs (systolic and diastolic blood pressure, heart rate, respiration rate, SpO_2, and body temperature) will be evaluated at the visits indicated in the Schedule of Assessments (Table 1). All vital signs will be measured after the patient has been resting for at least 5 minutes. Blood pressure measurements are to be taken in the same arm for the duration of the study. Moreover, additional vital signs may be performed as needed in case of any cardiac AE.

Additionally, body weight (without shoes) will be recorded at screening, Day 1, Day 14, Day 28, Day 60, and day of discharge (applicable); height (without shoes) will be recorded at screening only. Body mass index will be calculated whenever weight is recorded.

Vital sign measurements will be repeated if clinically significant or machine/equipment errors occur. Out-of-range blood pressure, respiratory rate, heart rate measurements, or SpO_2 will be

repeated at the investigator's discretion. Any confirmed, clinically significant vital sign measurements must be recorded as AEs.

12.3 Physical Examinations

A complete physical examination will be performed at screening. Physical examinations will be performed by a physician, nurse, or other appropriately trained staff. The complete physical examination includes head, eyes, ears, nose, and throat; heart; lungs; abdomen; skin; cervical and axillary lymph nodes; and neurological and musculoskeletal systems.

A limited physical examination to verify continued patient eligibility and to follow-up regarding any change in medical history will be performed at the visits indicated in the Schedule of Assessments (Table 1). Symptom-driven, limited physical examinations may be performed as clinically indicated during the study (according to SoC).

Please note: The limited physical examination on Day 8 (± 3 days) will be collected in all patients who remain hospitalized. If the patient is discharged before Day 8 (± 3 days), the site should arrange to have the physician assess the patient via a telephone/virtual call. If the physician determines during the telephone/virtual call that the limited physical examination is clinically indicated, then the site should arrange for an outpatient visit to collect this assessment.

12.4 Electrocardiograms

A 12-lead, resting ECG will be obtained locally at screening as indicated in the Schedule of Assessments (Table 1). At screening, the investigator must examine the ECG traces for clinically significant abnormalities that could exclude the patient from the study. If ECGs are performed at unscheduled time points (eg, according to SoC), findings must be recorded in the eCRF.

ECGs should always be obtained in supine position after adequate rest (≥ 5 minutes). An assessment of normal or abnormal will be recorded; if the ECG is considered abnormal, the abnormality will be documented on the eCRF. ECGs will be repeated if clinically significant abnormalities are observed or artifacts are present. Any ECG considered by the investigator to be clinically significant should be considered an AE. Moreover, additional ECGs may be obtained as needed in case of any cardiac AE and will be recorded on the eCRF.

12.5 Clinical Laboratory Assessments

Clinical laboratory assessment samples (Table 5) will be obtained at designated visits as specified in the Schedule of Assessments (Table 1).

Table 5: Clinical Laboratory Assessments

Hematology	Blood Biochemistry	Urinalysis (Dipstick)
Full and differential blood count<br>Hematocrit (HCT)<br>Hemoglobin (Hb)<br>Mean corpuscular hemoglobin (MCH)<br>Mean corpuscular hemoglobin concentration (MCHC)<br>Mean corpuscular volume (MCV)<br>Platelet count (PLT Ct)<br>Red blood cell count (RBC ct)<br>White blood cell count (WBC ct) with differential	Albumin (ALB)<br>Alanine aminotransferase (ALT)<br>Alkaline phosphatase (ALP)<br>Aspartate aminotransferase (AST)<br>Blood urea nitrogen (BUN) or urea<br>Carbon dioxide (CO_2)<br>Creatinine[a] (Cr)<br>Creatine kinase (CK) and subtypes<br>Electrolytes (sodium, potassium, chloride, calcium, phosphorus)<br>Gamma-glutamyl transpeptidase (GGT)<br>Glucose<br>Lactate dehydrogenase (LDH)<br>Total bilirubin (TBIL)<br>Direct bilirubin (DBI)<br>Total cholesterol (TC)<br>Triglycerides (TG)<br>Estimated glomerular filtration rate[b] (eGFR)	Appearance<br>pH<br>Protein<br>Glucose<br>Ketone bodies<br>Indicators of blood and white blood cells<br>Specific gravity<br>Urobilinogen (UROB)
**Coagulation**<br>Activated partial thromboplastin time<br>International normalized ratio<br>Partial thromboplastin time		
**Pregnancy Test**<br>A serum pregnancy (hCG) test will be performed on all WOCBP at screening and a urine pregnancy (hCG) test will be performed on all WOCBP on Day 1 before randomization and dosing. A urine pregnancy test will be repeated on Day 14 (± 7 days). If the patient is discharged before Day 14, urine pregnancy test at day of discharge should be done, and urine pregnancy test at Day 14 is not required. If the patient is discharged after Day 14, urine pregnancy test should be done at Day 14, and urine pregnancy test at day of discharge is not required (whichever comes first). Patients with a positive pregnancy test will be discontinued from study drug.<br>Please note: In questionable cases of postmenopausal status, a blood sample with simultaneous follicle stimulating hormone and estradiol levels may be required to determine postmenopausal status		

Abbreviations: hCG = human chorionic gonadotropin; WOCBP = women of childbearing potential.
[a] Creatinine clearance will be estimated using the patient's serum creatinine value, actual body weight, age, and the appropriate Cockroft-Gault formula for the patient's sex.
[b] According to Chronic Kidney Disease Epidemiology Collaboration (Levey et al 2009).

Blood and urine samples will be analyzed at the site's local laboratory facility. Urine samples will be analyzed by dipstick, and a microscopic analysis will be performed if the results of dipstick indicate abnormalities to be further investigated. All laboratory reports must be reviewed, signed, and dated by the investigator. A legible copy of all reports must be filed with both the patient's eCRF and medical record (source document) for that visit. Any laboratory test result considered by the investigator to be clinically significant should be considered an AE (clinically significant AEs include those that require an intervention). Clinically significant

This document is confidential.

abnormal values occurring during the study will be followed up until repeat test results return to normal, stabilize, or are no longer clinically significant. If laboratory assessments are performed at unscheduled time points (eg, according to SoC), findings must be recorded in the eCRF.

Please note: The clinical laboratory assessments on Day 8 (± 3 days) and Day 21 (± 2 days) will be collected in all patients who remain hospitalized. If the patient is discharged before Day 8 (± 3 days) or Day 21 (± 2 days), the site should arrange to have the physician assess the patient via a telephone/virtual call. If the physician determines during the telephone/virtual call that the clinical laboratory assessments are clinically indicated, then the site should arrange for an outpatient visit to collect this assessment. All efforts should be made to collect clinical laboratory assessments on Day 14 (± 2 days) and Day 28 (± 3 days).

12.6 Adverse Events

12.6.1 Adverse Events

An AE is any symptom, physical sign, syndrome, or disease that either emerges during the study or, if present at screening, worsens during the study, regardless of the suspected cause of the event. All medical and psychiatric conditions (except those related to the indication under study) present at screening will be documented on the medical history eCRF. Changes in these conditions and new symptoms, physical signs, syndromes, or diseases should be noted on the AE eCRF during the rest of the study. Clinically significant laboratory abnormalities should also be recorded as AEs. Surgical procedures that were planned before the patient was randomized in the study are not considered AEs if the conditions were known before study inclusion; the medical condition should be reported in the patient’s medical history.

Patients will be instructed to report AEs at each study visit. All AEs are to be followed up until resolution or a stable clinical endpoint is reached.

Each AE is to be documented on the eCRF with reference to date of onset, duration, frequency, severity, relationship to study drug, action taken with study drug, treatment of event, and outcome. Furthermore, each AE is to be classified as being serious or nonserious. Changes in AEs and resolution dates are to be documented on the eCRF.

For the purposes of this study, the period of observation for collection of AEs extends from the time the patient gives informed consent until the end of the follow-up period (Day 60). Follow-up of the AE, even after the date of study completion, is required (if possible) if the AE persists. The follow-up should continue until the event resolves (returns to normal or baseline values) or stabilizes, or until it is judged by the investigator to no longer be clinically significant. It is understood that if a patient withdraws consent to further follow-up, then it may not be possible for the study site to follow the AE to resolution.

When changes in the severity of an AE occur more frequently than once a day, the maximum severity for the event should be noted. If the severity category changes over a number of days, then those changes should be recorded separately (with distinct onset dates).

This document is confidential.

Each AE will be assigned a grade which refers to the severity of the AE based on the Common Terminology Criteria for Adverse Events version 5.0. The Common Terminology Criteria for Adverse Events v5.0 displays grades 1 through 5 with unique clinical descriptions of the severity for each AE based on the general guideline shown in Table 6.

The guideline for classifying AEs by relationship to study drug are given in Table 7.

Table 6: Classification of Adverse Events by Severity

**Grade 1**: Mild; asymptomatic or mild symptoms; clinical or diagnostic observations only; intervention not indicated.
**Grade 2**: Moderate; minimal, local or non-invasive intervention indicated; limiting age-appropriate instrumental activities of daily living.
**Grade 3**: Severe or medically significant but not immediately life-threatening; hospitalization or prolongation of hospitalization indicated; disabling; limiting self-care activities of daily living.
**Grade 4**: Life-threatening consequences; urgent intervention indicated.
**Grade 5**: Death related to AE.

Abbreviation: AE = adverse event.

Table 7: Classification of Adverse Events by Relationship to Study Drug

**UNRELATED**: This category applies to those AEs that are clearly and incontrovertibly due to extraneous causes (disease, environment, etc).
**UNLIKELY**: This category applies to those AEs that are judged to be unrelated to the study drug but for which no extraneous cause may be found. An AE may be considered unlikely to be related to study drug if or when it meets 2 of the following criteria: (1) it does not follow a reasonable temporal sequence from administration of the study drug; (2) it could readily have been produced by the patient's clinical state, environmental or toxic factors, or other modes of therapy administered to the patient; (3) it does not follow a known pattern of response to the study drug; or (4) it does not reappear or worsen when the drug is readministered.
**POSSIBLY**: This category applies to those AEs for which a connection with the study drug administration appears unlikely but cannot be ruled out with certainty. An AE may be considered possibly related if or when it meets 2 of the following criteria: (1) it follows a reasonable temporal sequence from administration of the drug; (2) it could not readily have been produced by the patient's clinical state, environmental or toxic factors, or other modes of therapy administered to the patient; or (3) it follows a known pattern of response to the study drug.
**PROBABLY**: This category applies to those AEs that the investigator feels with a high degree of certainty are related to the study drug. An AE may be considered probably related if or when it meets 3 of the following criteria: (1) it follows a reasonable temporal sequence from administration of the drug; (2) it could not be reasonably explained by the known characteristics of the patient's clinical state, environmental or toxic factors, or other modes of therapy administered to the patient; (3) it disappears or decreases on cessation or reduction in dose (note that there are exceptions when an AE does not disappear upon discontinuation of the drug, yet drug-relatedness clearly exists; for example, as in bone marrow depression, fixed drug eruptions, or tardive dyskinesia); or (4) it follows a known pattern of response to the study drug.
**DEFINITELY**: This category applies to those AEs that the investigator feels are incontrovertibly related to study drug. An AE may be assigned an attribution of definitely related if or when it meets all of the following criteria: (1) it follows a reasonable temporal sequence from administration of the drug; (2) it could not be reasonably explained by the known characteristics of the patient's clinical state, environmental or toxic factors, or other modes of therapy administered to the patient; (3) it disappears or decreases on cessation or reduction in dose and recurs with reexposure to drug (if rechallenge occurs); and (4) it follows a known pattern of response to the study drug.

Abbreviation: AE = adverse event.

12.6.2 Serious Adverse Events

An SAE is any untoward medical occurrence, in the view of either the investigator or sponsor, that:

- results in death
- is life-threatening
- results in inpatient hospitalization or prolongation of existing hospitalization
- results in persistent or significant disability/incapacity
- is a congenital anomaly/birth defect

Other important medical events that may not be immediately life-threatening or result in death or hospitalization, based upon appropriate medical judgment, are considered SAEs if they are thought to jeopardize the patient and/or require medical or surgical intervention to prevent 1 of the outcomes defining an SAE. SAEs are critically important for the identification of significant safety problems; therefore, it is important to take into account both the investigator's and the sponsor's assessment. If either the sponsor or the investigator believes that an event is serious, the event must be considered serious and evaluated by the sponsor for expedited reporting.

12.6.3 Serious Adverse Event Reporting

An SAE occurring from the time the patient gives informed consent until the end of the follow-up period (Day 60) or within 30 days of stopping the study drug (whichever is longer) must be reported to the Syneos Health Safety and Pharmacovigilance group and will be communicated to the sponsor. Any such SAE due to any cause, whether or not related to the study drug, must be reported within 24 hours the investigator's awareness of the event. Notification can be made using the dedicated fax line or email for the Syneos Health Safety and Pharmacovigilance group:

- Syneos Health Safety and Pharmacovigilance fax number: +1 (877) 464-7787
- Syneos Health Safety and Pharmacovigilance email address: safetyreporting@syneoshealth.com

If the investigator contacts the Syneos Health Safety and Pharmacovigilance group by telephone, then a written report must follow within 24 hours and has to include a full description of the event and sequelae in the format detailed in the SAE reporting form.

The event must also be recorded on the standard AE eCRF. Preliminary reports of SAEs must be followed up by detailed descriptions later on. Clear and anonymized photocopies of hospital case reports, consultant reports, autopsy reports, and other documents must be provided when requested by Syneos Health Safety and Pharmacovigilance. SAE reports must be made whether or not the investigator considers the event to be related to the investigational drug.

Appropriate remedial measures should be taken to treat the SAE, and the response should be recorded. Clinical, laboratory, and diagnostic measures should be employed as needed to determine the etiology of the problem. The investigator must report all additional follow-up evaluations to the Syneos Health Safety and Pharmacovigilance group within 24 hours of becoming aware of the additional information. All SAEs will be followed up until the investigator and sponsor agree the event is satisfactorily resolved.

Any SAE that is not resolved by the end of the study or upon discontinuation of the patient's participation in the study is to be followed up, if possible, until it either resolves, stabilizes, returns to baseline values (if a baseline value is available), or is shown to not be attributable to the study drug or procedures. It is understood that if a patient withdraws consent to further follow-up, then it may not be possible for the study site to follow the SAE to resolution.

This document is confidential.

12.6.4 Suspected Unexpected Serious Adverse Reactions

AEs that meet all of the following criteria will be classified as suspected unexpected serious adverse reactions (SUSARs) and reported to the appropriate regulatory authorities in accordance with applicable regulatory requirements for expedited reporting:

- serious
- unexpected (ie, the event is not consistent with the reference safety information in the current version of the IB)
- there is at least a reasonable possibility that there is a causal relationship between the event and the study drug

The investigator will assess whether an event is causally related to the study drug. Syneos Health will consider the investigator's assessment and determine whether the event meets the criteria for being reportable as a 7-day or 15-day safety report. SUSARs that are fatal or life-threatening must be reported to the regulatory authorities and the IRBs/IECs (where required) within 7 days after Syneos Health/MetrioPharm has first knowledge of them, with a follow-up report (when applicable) submitted within a further 8 calendar days. Other SUSARs must be reported to the relevant regulatory authorities and the IRBs/IECs within 15 calendar days after Syneos Health/MetrioPharm first has knowledge of them.

Syneos Health is responsible for reporting SUSARs and any other events required to be reported in an expedited manner to the regulatory authorities and for informing investigators of reportable events, in compliance with applicable regulatory requirements within specific timeframes. Investigators will notify the relevant IRBs/IECs of reportable events within the applicable timeframes.

12.6.5 Pregnancy

WOCBP must have a negative serum pregnancy test at screening and negative urine pregnancy test before the first dose on Day 1. A urine pregnancy test will be repeated on Day 14 (± 7 days). If the patient is discharged before Day 14, urine pregnancy test at day of discharge should be done, and urine pregnancy test at Day 14 is not required. If the patient is discharged after Day 14, urine pregnancy test should be done at Day 14, and urine pregnancy test at day of discharge is not required (whichever comes first). Patients with a positive pregnancy test will be discontinued from study drug. After administration of study drug, any known cases of pregnancy in female patients will be reported until the patient completes or withdraws from the study. The pregnancy will be reported immediately by faxing/emailing a completed pregnancy report to Syneos Health Safety and Pharmacovigilance within 24 hours of knowledge of the event. The pregnancy will not be processed as an SAE; however, the investigator will follow up with the patient until completion of the pregnancy and must assess the outcome in the shortest possible time but not >30 days after completion of the pregnancy. (It is understood that if a patient withdraws consent to further follow-up, then it may not be possible for the study site to follow the pregnancy to completion.) The investigator should notify Syneos Health Safety and

Pharmacovigilance group of the pregnancy outcome by submitting a follow-up pregnancy report. If the outcome of the pregnancy involved spontaneous or therapeutic abortion (any congenital anomaly detected in an aborted fetus is to be documented), stillbirth, neonatal death, or congenital anomaly, the investigator will report the event by faxing/emailing a completed pregnancy report form to Syneos Health Safety and Pharmacovigilance group within 24 hours of knowledge of the event.

If the investigator becomes aware of a pregnancy occurring in the partner of a patient participating in the study, the pregnancy should be reported to Syneos Health Safety and Pharmacovigilance group within 24 hours of knowledge of the event. Information regarding the pregnancy must only be submitted after obtaining consent from the pregnant partner. The investigator will arrange counseling for the pregnant partner by a specialist to discuss the risks of continuing with the pregnancy and the possible effects on the fetus.

Upon discontinuation from the study drug, only those procedures that would not expose the patient to undue risk will be performed. The investigator should also be notified of pregnancy occurring during the study but confirmed after completion of the study. In the event, that a patient is subsequently found to be pregnant after inclusion in the study and received at least one dose of study drug, any pregnancy will be followed to term, and the status of mother and child will be reported to Syneos Health Safety and Pharmacovigilance group after delivery.

12.6.6 Overdose

The investigator must immediately notify Syneos Health of any occurrence of overdose with the study drug.

13 PHARMACOKINETICS

13.1 Pharmacokinetic Sampling

Blood samples for PK analysis of MP1032 plasma concentrations will be collected at the time points indicated in the Schedule of Assessments (Table 1) from a PK subset of approximately 30 patients (who give optional consent). In summary, PK samples will be collected via sparse sampling from these 30 patients (approximately) in a 2:1 ratio (approximately 20 patients from the 300 mg MP1032 BID plus SoC group and approximately 10 patients from the placebo group plus SoC group) to assess the PK of MP1032 on Day 1 (single dose) and Day 7 (steady state). A total of 16 samples (approximately 4.9 mL per time point for a total of approximately 78.4 mL) will be collected relative to the first dose on Day 1 and the first dose on Day 7. Samples on Day 1 and Day 7 will be collected predose (within 10 minutes before the first daily dose) and postdose at 10 minutes, 20 minutes, 30 minutes, 60 minutes, 120 minutes, 8 hours (before next dose), and 24 hours before the next morning dose (if applicable), if possible. Patients who provide consent for the PK sampling, but are no longer hospitalized on Day 7, will not have Day 7 PK samples collected.

For patients in the PK subset on Day 1 and Day 7 only: Patients should not eat within the 4 hours before planned study drug administration and within the 120 minutes after study drug dosing, when feasible. Thus, study drug should be administered in fasted state (≥ 4 hours), when feasible. However, if a patient has eaten within the 4 hours before their planned administration, study drug dosing may proceed. The patient's fed or fasted state, as well as food intake (yes/no) within the 120 minutes after study drug dosing should be recorded. The PK samples should be collected as planned regardless of the patients' fasted/fed state. The study site should record the date and time of dosing (recorded as the time when the first capsule is administered).

Intravenous cannulas may be used for blood collection to avoid multiple skin punctures, when appropriate. Otherwise, blood samples will be collected by direct venipuncture. The actual date and time of each blood sample collection will be recorded. Sample collections completed outside the predefined time windows (windows to be specified in the laboratory manual) will not be considered as protocol deviations because actual postdose sampling times will be used for PK and statistical analyses.

PK samples will be analyzed by a central laboratory facility (Prolytic GmbH). Details of PK blood sample collection, processing (including separation of plasma), storage, and shipping procedures will be provided in a separate laboratory manual.

13.2 Pharmacokinetic Analytical Methodology

The concentration of study drug will be determined from the plasma samples using a validated analytical method. Details of the method validation and sample analysis will be included with the final clinical study report (CSR).

14 OTHER ASSESSMENTS

14.1 Biomarkers

Blood samples will be obtained at the visits specified in the Schedule of Assessments (Table 1) on Day 1 (before the first study drug administration) and Days 7, 14, 21, 28, and 60 (when possible) to measure biomarkers levels. Blood samples for biomarkers must be collected on Days 7, 14, 21, 28, and 60 if a patient is still hospitalized. If a patient is discharged, the study site should arrange an outpatient visit (ie, at the study site) to accommodate the collection of blood samples for biomarkers on Days 7, 14, 21, 28, and 60.

Biomarkers will potentially include, but will not be limited, to cytokines (eg, C-reactive protein, IL-1β, IL-6, TNF-α, and IFN-γ), and other coagulation/inflammatory biomarkers (eg, D-dimer and ferritin). Samples may be collected at the same time that samples are collected for the clinical laboratory assessments. Blood samples will be analyzed by a central laboratory facility. Details of sample collection, processing, storage, shipping procedures, and analysis will be provided in a separate laboratory manual.

Please note: Patients will be requested to provide consent for the sponsor to store and use leftover blood samples for future exploratory biomarker research. Patient participation in the storage and use of leftover blood samples is optional. If the patient consents, the sponsor will store and use the leftover blood samples for a period of 3 years after the last patient has completed his/her last visit in this study.

15 STATISTICAL ANALYSIS

The statistical analysis plan (SAP) will provide further details regarding the definition of analysis variables and analysis methodology to address all study objectives. The SAP will be finalized before any statistical output (eg, DSMB output) is produced. The SAP will serve as a companion document to the protocol. The SAP will further describe the statistical analyses. All data will be included with the final CSR.

The statistical evaluation will be performed using SAS® software version 9.4 or higher (SAS® Institute, Cary, NC).

Summary statistics will be presented by treatment group. Continuous and ordinal variables will be summarized by the number of patients (N), mean, standard deviation, median, minimum, and maximum for actual values and change from baseline. Additionally, for ordinal data, the number and percentage of patients in each category will be presented. Binary and categorical variables will be summarized for each treatment group by presenting the number and percentage of patients in the categories.

Statistical tests for comparison between treatments will be performed using a two-sided alpha level of 10%. No adjustment for multiple testing will be applied in this descriptive proof-of-concept study.

15.1 Determination of Sample Size

The primary efficacy endpoint "proportion of patients with disease progression on Day 14" is assumed to be 10% in the 300 mg MP1032 BID plus SoC treatment group (Arm A) and 30% in the placebo plus SoC treatment group (Arm B). Using the Chi-square test with type I error alpha=10% two-sided for this proof-of-concept study and 2:1 randomization ratio, with 114 randomized patients (76 in Arm A and 38 in Arm B) a statistical power of 83% can be achieved. Considering about 5% early study terminations, the necessary sample size to randomize results is 120 patients in total (80 in Arm A and 40 in Arm B). If during the conduct of the study the early study termination rate is higher than the estimated 5%, then an increase in the randomized number of patients may be necessary.

Sample size estimation was performed using nQuery 8, Version 8.6.1.0.

A subset of randomized patients will be consented for the collection of blood samples for PK measurements. The PK subset consists of approximately 20 patients from the 300 mg MP1032 BID plus SoC group and approximately 10 patients from the placebo plus SoC group, up to a total of approximately 30 patients.

15.2 Analysis Sets

Screened Set

The Screened Set will include all patients who give informed consent and are screened. For this group, patient disposition, and reasons for premature study termination or screening failure will be described.

Randomized Set

The Randomized Set will include all patients randomized. Unless specified otherwise, this set will be used for all subject listings with exception of the listing presenting screening failures and the respective reason(s) for screen failure.

Intention-To-Treat Set

The Intention-to-Treat (ITT) set, correspond with randomized set, will include all randomized patients irrespective of any deviation from the protocol or premature discontinuation from study drug/withdrawal from study. The treatment group assignment will be designated according to initial randomization. The ITT will serve as the basis for the analysis of efficacy and summary of demographics and baseline characteristics.

Per Protocol Set

The Per Protocol Set (PPS) will include all patients from ITT who received at least 1 dose of study drug and who do not have any major protocol deviations impacting the efficacy assessments. The PPS will be used for supportive analyses of efficacy.

Safety Set

The Safety Set (SS) will include all randomized patients who receive at least 1 dose of study drug. The treatment group assignment in the SS will be defined by the treatment actually received. The SS will be used for the analysis of safety.

PK Analysis Set

The PK Analysis Set (PKS) will include all the patients who have been administered active study drug and have at least 1 postdose evaluable plasma concentration after Day 1 dose.

15.3 Efficacy Analysis

The estimands for the primary efficacy endpoint, and for the 4 key secondary endpoints comparing the 2 treatment groups, will be tested with consideration of a two-sided type-1 error alpha of 10% for each test on the ITT. Additionally, 95% confidence intervals will be provided. Also, the treatment comparison with regards to the other secondary efficacy endpoints will be performed using a two-sided alpha level of 10%.

The main estimand for the primary efficacy endpoint is defined as follows:

- Treatment: 300 mg MP1032 BID plus SoC in Arm A and placebo BID plus SoC in Arm B. If a patient terminates the study drug, but still remains in the study and is followed up, then the patient will continue to receive either SoC alone or SoC+ additional treatment. These treatments are defined with regards to intercurrent events as ‘other treatments’.
- Population: Of all patients defined by the study inclusion/exclusion criteria, the analysis population will include patients who were randomized to study drug (ITT population).
- Variable: A binary variable indicating disease progression (death or respiratory failure) at Day 14. Respiratory failure is defined as patients who have a score of 2, 3, or 4 on the NIAID 8-point ordinal scale.
- Intercurrent events: All events that lead to study drug discontinuation and/or switch to other treatment before Day 14 will be handled using the treatment-policy strategy (ie, the NIAID score at Day 14 will be used for definition of the failure in such patients).
- Population-level summary: Common risk difference resulting from the Mantel-Haenszel (MH) test considering the 4 strata out of the combinations of the 2 randomization stratification factors disease severity (moderate versus severe) and age-class (aged ≤65 years versus >65 years) at baseline.

Missing data on Day 14 resulting from study termination before Day 14 are not expected. However, in case they happen, such monotone missings will be imputed by multiple imputation using information from similar patients of the same treatment group. Missing postbaseline data for patients randomized but not treated will be imputed by the respective baseline value (baseline observation carried forward [BOCF]). The comparison between the treatment groups will be performed on the ITT after imputation of missing values using MH test for common risk difference considering the 4 strata resulting from the 2 randomization stratification factors disease severity (moderate versus severe) and age-class (aged ≤65 years versus >65 years). The 95% confidence intervals will be provided for the risk difference.

Similar MH analyses and presentation of 95% confidence intervals will be applied to the main estimand of the first key secondary efficacy endpoint, which is defined as a binary variable indicating disease progression (death or respiratory failure) at Day 28, and the main estimand of the second key secondary efficacy endpoint that is defined as a binary variable indicating disease resolution (alive and have a score of 6, 7 or 8 on NIAID) at Day 28. Intercurrent events that lead to study drug discontinuation and/or switch to other treatment before Day 28 will be handled using the treatment-policy strategy for these 2 estimands (ie, the NIAID score at Day 28 will be used for definition of the failure or response, respectively) in such patients. Missing data on Day 28 will be imputed by multiple imputation using information from similar patients of the same treatment group. Missing postbaseline data for patients randomized but not treated will be imputed by BOCF.

A similar MH approach and presentation of 95% confidence intervals as described above will be applied also to the main estimand of the third key secondary efficacy endpoint, which is the all-cause mortality at Day 28. Intercurrent events that lead to study drug discontinuation and/or

switch to other treatment before Day 28 will be handled using the treatment-policy strategy (ie, the life status at Day 28 will be considered). No missing data of life status on Day 28 is expected.

The main estimand of the fourth key secondary efficacy endpoint, change of clinical status related to COVID-19 on Day 28 compared with baseline according to the NIAID 8-point ordinal scale, will be compared using the risk difference from the analysis of covariance model with treatment and the 2 binary stratification factors (eg, severity and age-class) as independent factors and the baseline value of NIAID as independent covariate. Intercurrent events that lead to study drug discontinuation and/or switch to other treatment before Day 28 will be handled using the treatment-policy strategy for this estimand (ie, the NIAID score at Day 28 will be used). Missing data on Day 28 will be imputed by multiple imputation using information from similar patients of the same treatment group. Missing post-baseline data for patients randomized but not treated will be imputed by BOCF. The 95% confidence intervals will be provided for the risk difference.

The population for all above described estimands of the key secondary endpoints is the ITT.

Sensitivity analyses for the main estimands of the primary and key efficacy endpoints will be performed by assuming missing data at Day 14 or Day 28 as failure or non-resolution for the binary endpoints, and by multiple imputation using information from similar patients of the placebo group for the NIAID 8-point ordinal scale.

All above analyses for the main estimands of the primary efficacy and key secondary efficacy endpoints will be repeated on PPS as supportive analysis. Additionally, for the primary efficacy and binary key secondary efficacy endpoints, logistic regression model with treatment and the 2 stratification factors as covariables will be performed on the ITT. Further estimands for the primary and key secondary efficacy endpoints will be provided in the SAP.

The proportion of patients with disease resolution at Day 14 and the all-cause mortality rate at Day 14 and Day 60 will be analyzed using similar MH methods as described above for Day 28. Cumulative proportion of patients with disease resolution will be plotted by time.

A similar analysis of covariance approach as described above for Day 28 will be applied for the analysis of the change of clinical status related to COVID-19 on Day 14 compared with baseline on the NIAID 8-point ordinal scale. The endpoint considering the proportion of patients requiring invasive ventilation (mechanical ventilator and/or ECMO) or who are not alive, the proportion of patients in each NIAID category, and the proportion of patients alive and testing negative for COVID-19 on Day 14, Day 28, and Day 60, respectively, will be compared between the treatment groups using MH test. Overall survival, time to (first) improvement of at least 1 category on the NIAID scale and total hospitalization duration (from baseline to discharge; with death censored on last day of the observed period − at Day 28 or Day 60, respectively − depending on the analyses) will be summarized using the Kaplan-Meier method and will be compared between treatment groups using log-rank tests. Additionally, Cox proportional hazards model will be applied with treatment and the 2 stratification factors as covariables.

This document is confidential.

The odds ratio between MP1032 and SoC and placebo and SoC for the number of patients with clinical status improvement from baseline on the NIAID 8-point ordinal scale (ie, an improvement of at least 1 category) at Day 14 and Day 28 will be analyzed using a logistic regression with consideration of the 2 stratification factors.

The time to recovery of all at baseline present COVID-19 symptoms (stuffy or runny nose, sore throat, red or irritated eyes, shortness of breath, cough, low energy or tiredness, muscle or body aches, headache, chills or shivering, feeling hot or feverish, nausea, and number of times of vomit, times of diarrhea, sense of smell, sense of taste in the last 24 hours) will be presented by Kaplan-Meier estimates and figure. The change from baseline will be presented in shift tables for each single symptom, with the number and percentage of subjects in each category at post-baseline assessment time-point compared to baseline for each treatment group.

Total number of days in ICU, duration of invasive mechanical ventilation, and duration of ECMO will be descriptively summarized by treatment group using mean days with 90% confidence intervals and additionally by median days and quartiles. To consider death sufficiently unfavorable in these measures, in case of death, the duration of the respective measure will be considered with the longest duration possible (eg, if a summary of the first 28 days is presented, then death before Day 28 will be considered as duration 28 days, and if the summary also includes follow-up period until Day 60, then death before Day 60 will be considered as duration of 60 days). Both summaries (up to Day 28 and up to Day 60) will be provided.

For HRQoL assessments at discharge and Day 60, the EQ index value and the EQ VAS, both based on the EQ-5D-5L questionnaire (Appendix 2), will be calculated and summarized by visit and treatment group. Additionally, change at Day 60 from discharge will be summarized by treatment group.

All efficacy endpoints will be summarized using descriptive statistics by treatment group and by visit, as appropriate: Binary and categorical endpoints will be summarized by treatment group by presenting the number and percentage of patients in the categories. Continuous and ordinal endpoints will be described by mean, standard deviation, median, minimum, and maximum for actual values and change from baseline. Additionally, for ordinal data, the number and percentage of patients in each category will be presented.

15.4 Safety Analysis

All safety parameters will be summarized descriptively by treatment group for the entire study period.

Study drug duration and SoC therapies will be described for each treatment group.

All reported AEs will be coded into preferred term (PT) and grouped by SOC using the most recent Medical Dictionary for Regulatory Activities version. An overall summary will be provided with the number and percentage of patients reporting TEAEs, serious TEAEs,

treatment-related TEAEs, TEAEs leading to withdrawal, and TEAEs leading to death. For patients with any serious TEAE and for patients with any related serious TEAE exposure-adjusted events rates will be calculated and compared between treatment groups using the risk ratio [(300 mg MP1032 BID plus SoC) / (placebo plus SoC)] with respective 95% confidence intervals. This comparison will also be performed for the rate of rehospitalization which is considered a serious AE. The number and percentage of patients with TEAEs, serious TEAEs, TEAEs leading to withdrawal, and treatment-related TEAEs will be summarized by SOC and PT for each treatment group, and total. In these tables, the incidence of TEAEs by SOC and PT will also be included. TEAEs will also be summarized by maximum severity and relationship to study drug. Events with missing onset dates will be considered as TEAEs. If a patient experienced >1 occurrence of the same AE, the occurrence with the greatest severity and the closest association with the study drug will be used in the corresponding summary tables. In case that for one AE several AE episodes with changing severity is reported, an aggregation of all respective episodes to one AE will be performed before summarization. The worst severity and the worst causality will be assigned to the aggregated AE and used for the respective AE summaries. All reported AE episodes will be listed by patient, along with information regarding onset, duration, severity and relationship to study drug, action taken with study drug, treatment of event, and outcome. Additionally, the aggregated AEs with the resulting worst severity and worst relationship will be included in the AE listings.

Vital signs including SpO_2 (%) will be summarized using descriptive statistics, including mean values and mean change from baseline values. Clinical laboratory data of local laboratories will be presented with the number and percentage of patients with values below, within, or above the normal range at each time point. Additionally, a shift table of the number and percentage of patients with normal and abnormal, and clinically significant abnormal values at each scheduled postbaseline visit compared with the normal/abnormal/clinically significant abnormal categorization at baseline will be provided by treatment group. Summarizing of clinical laboratory data of local laboratories using descriptive statistics, including mean values and mean change from baseline values will be only possible after normalization of the values using a reference laboratory. Details for normalization will be provided in the SAP.

Summary tables will be provided for concomitant medications.

15.5 Pharmacokinetic Analysis

The PK parameters (such as C_{max}, T_{max}, AUC_{0-t}, and others) of MP1032 on Day 1 (single dose) and Day 7 (steady state; if available), using up to 8 hours postdose, will be derived applying non-compartmental analysis. PK parameters will be summarized along with plasma concentrations at various time points. Individual and mean plasma concentration versus time will be presented on linear, and semi-log scales. Further details of the PK analysis will be described in the SAP. Any possible correlation between plasma exposure and biomarkers (eg, change from baseline in cytokines, or other coagulation/inflammatory biomarkers) or efficacy measures can be explored by appropriate regression analysis methods during the final data analysis.

15.6 Biomarker Analysis

The change from baseline biomarker levels will be summarized descriptively by treatment group and visit. The change in biomarker levels will also be evaluated with any change of COVID-19 clinical status.

15.7 Interim Analysis

Not applicable.

15.8 Data Monitoring Committee

An independent DMC will be established by the sponsor or designee to review accumulating study data at regular intervals (as per the DMC charter) throughout the study to ensure the safety of patients and review overall study conduct. Additional data may be reviewed that are related to efficacy, but the DMC will only be reviewing data for risk-benefit purposes. Members will include experts in drug safety and/or medical specialist and biostatistics, who are not participating in this study and do not have affiliation with the investigators or the sponsor. The DMC can recommend in writing to the sponsor whether to continue, modify, or stop the clinical study on the basis of safety considerations. The pre-specified study stopping rules for the trial are defined as follows:

- death (not including disease progression) in any patient in whom the cause of death is judged to be related to the study drug by investigator
- the occurrence in any patient of a SAE whose causal relationship (ie, without a plausible alternative explanation) to the study drug is judged to be related by investigator
- two occurrences of a clinically significant Grade 3 or higher laboratory abnormality assessed to be related to the study drug by investigator.

The DMC’s specific duties will be fully described in a DMC charter.

16 STUDY MANAGEMENT

16.1 Approval and Consent

16.1.1 Regulatory Guidelines

This study will be conducted in accordance with the accepted version of the Declaration of Helsinki and all relevant regulations as set forth in Parts 50, 56, 312, Subpart D, of Title 21 of the United States Code of Federal Regulations (CFR), in compliance with International Council for Harmonisation and GCP guidelines and according to the appropriate regulatory requirements in the countries where the study will be conducted.

16.1.2 Institutional Review Board/Independent Ethics Committee

Conduct of the study must be approved by an appropriately constituted IRB/IEC. Approval is required for the study protocol, protocol amendments (if applicable), IB, ICFs, recruitment material and patient information sheets, and other patient-facing material.

16.1.3 Informed Consent

For each patient (or the patient's LAR), informed consent will be obtained before any protocol-related activities. As part of this procedure, the principal investigator (PI) or designee must explain the nature of the study, its purpose, procedures, expected duration, alternative therapy available, and the benefits and risks involved in study participation. The patient (or the patient's LAR) should be informed that he/she may withdraw from the study at any time, and the patient will receive all information that is required by local regulations and guidelines for International Council for Harmonisation. The PI will provide the sponsor or its representative with a copy of the IRB/IEC-approved ICF before the start of the study.

16.2 Data Handling

Data reported on the eCRF that are derived from source documents should be consistent with the source documents, or the discrepancies must be explained (see also Section 16.3).

Clinical data will be entered by site personnel on eCRFs for transmission to the sponsor. Data on eCRFs transmitted via the web-based data system must correspond to and be supported by source documentation maintained at the study site. All study forms and records transmitted to the sponsor must only include coded identifiers such that directly identifying personal information is not transmitted. The primary method of data transmittal is via the secure, internet-based electronic data capture (EDC) system maintained by Syneos Health. Access to the EDC system is available to only authorized users via the study's internet website, where a user unique assigned username and password are required for access.

Any changes made to data after collection will be made through the use of data clarification forms or the EDC system. When all missing and/or incorrect data have been resolved, eCRFs will be considered complete.

16.3 Source Documents

Source documents are considered to be all information in original records and certified copies of original records of clinical findings, observations, data, or other activities in a clinical study necessary for the reconstruction and evaluation of the study. The investigator will provide direct access to source documents and/or source data in the facilitation of study-related monitoring, audits, review by IRB/IECs, and regulatory inspections.

The investigator/institution should maintain adequate and accurate source documents and study records that include all pertinent observations on each of the site's patients. Source data should

be attributable, legible, contemporaneous, original, accurate, and complete. Changes to source data should be traceable, not obscure the original entry, and be explained if necessary.

16.4 Record Retention

Study records and source documents must be preserved for at least 30 years after the completion or discontinuation of/withdrawal from the study, at least 2 years after the drug being studied has received its last approval for sale, or at least 2 years after the drug development has stopped, and in accordance with the applicable local privacy laws, whichever is the longer period.

The investigator agrees to comply with all applicable federal, state, and local laws and regulations relating to the privacy of patient health information, including, but not limited to, the Standards for Individually Identifiable Health Information, 45 CFR, Parts 160 and 164 (the Health Insurance Portability Accountability Act of 1996 Privacy Regulation). The investigator shall ensure that study patients authorize the use and disclosure of protected health information in accordance with Health Insurance Portability Accountability Act Privacy Regulation and in a form satisfactory to the sponsor.

16.5 Monitoring

The study will be monitored to ensure that it is conducted and documented properly according to the protocol, GCP, and all applicable regulatory requirements.

Monitoring visits, on-site and remote (telephone), or a combination of these and contacts will be made at appropriate times during the study. The PI will assure he/she and adequate site personnel must be available throughout the study to collaborate with clinical monitors. Clinical monitors must have direct access to source documentation to check the completeness, clarity, and consistency of the data recorded in the eCRFs for each patient.

The investigator will make available to the clinical monitor all source documents and medical records necessary to review protocol adherence and eCRFs. In addition, the investigator will work closely with the clinical monitor and, as needed, provide them appropriate evidence that the study is being conducted in accordance with the protocol, applicable regulations, and GCP guidelines.

16.6 Quality Control and Quality Assurance

The sponsor or its designee will perform the quality assurance and quality control activities of this study; however, responsibility for the accuracy, completeness, security, and reliability of the study data presented to the sponsor lies with the investigator generating the data.

The sponsor may arrange audits as part of the implementation of quality assurance to ensure that the study is being conducted in compliance with the protocol, standard operating procedures, GCP, and all applicable regulatory requirements. Audits will be independent of and separate from the routine monitoring and quality control functions. Quality assurance procedures will be

performed at study sites and during data management to assure that safety and efficacy data are adequate and well documented.

16.7 Protocol Amendment and Protocol Deviation

16.7.1 Protocol Amendment

Amendments to the protocol that entail corrections of typographical errors, clarifications of confusing wording, changes in study personnel, and minor modifications that have no effect on the safety of patients or the conduct of the study will be classed as administrative amendments and will be submitted to the IRB/IEC for information only. The sponsor will ensure that acknowledgment is received and filed. Amendments that are classed as substantial amendments must be submitted to the appropriate regulatory authorities and the IRB/IECs for approval and will not be implemented at sites until such approvals are received other than in the case of an urgent safety measure.

16.7.2 Protocol Deviations

Should a protocol deviation occur, the sponsor must be informed as soon as possible. Protocol deviations and/or violations and the reasons they occurred will be included in the CSR. Reporting of protocol deviations to the IRB/IEC and in accordance with applicable regulatory authority mandates is the investigator's responsibility.

16.8 Ethical Considerations

This study will be conducted in accordance with this protocol, the accepted version of the Declaration of Helsinki and/or all relevant federal regulations, as set forth in Parts 50, 56, 312, Subpart D, of Title 21 of the CFR; EU 536/2014, Annex 1, D, 17 (a); and in compliance with GCP guidelines.

IRB/IECs will review and approve this protocol and the ICF. All patients (or the patient's LAR) are required to give informed consent before participation in the study.

16.9 Financing and Insurance

Before the study commences, the sponsor (or its designee) and the investigator (or the institution, as applicable) will agree on costs necessary to perform the study. This agreement will be documented in a financial agreement that will be signed by the investigator (or the institution signatory) and the sponsor (or its designee).

The investigator is required to have adequate current insurance to cover claims for negligence and/or malpractice. The sponsor will provide no-exclusion insurance coverage for the clinical study as required by national regulations.

16.10 Publication Policy/Disclosure of Data

Both the use of data and the publication policy are detailed within the clinical study agreement. Intellectual property rights (and related matters) generated by the investigator and others performing the clinical study will be subject to the terms of a clinical study agreement that will be agreed between the institution and the sponsor or their designee. With respect to such rights, the sponsor or its designee will solely own all rights and interests in any materials, data, and intellectual property rights developed by investigators and others performing the clinical study described in this protocol, subject to the terms of any such agreement. In order to facilitate such ownership, investigators will be required to assign all such inventions either to their institution or directly to the sponsor or its designee, as will be set forth in the clinical study agreement.

17 REFERENCES

Beigel JH, Tomashek KM, Dodd LE, et al. ACTT-1 Study Group Members. Remdesivir for the treatment of Covid-19 - final report. *N Engl J Med*. Published online October 8, 2020. doi:10.1056/NEJMoa2007764.

Cennimo DJ. Coronavirus Disease 2019 (COVID-19). Updated January 09, 2021. Accessed January 13, 2021. https://emedicine.medscape.com/article/2500114-overview.

Charlson ME, Pompei P, Ales KL, MacKenzie CR. A new method of classifying prognostic comorbidity in longitudinal studies: development and validation. *J Chronic Dis*. 1987;40(5):373-383. doi:10.1016/0021-9681(87)90171-8.

Curtin N, Bányai K, Thaventhiran J, et al. Repositioning PARP inhibitors for SARS-CoV-2 infection (COVID-19); a new multi-pronged therapy for acute respiratory distress syndrome? *Br J Pharmacol*. 2020;177(16): 3635-3645. doi:10.1111/bph.15137.

EuroQol Research Foundation. EQ-5D Instruments. Accessed October 9, 2020. https://euroqol.org/eq-5d-instruments/.

Fanelli V, Vlachou A, Ghannadian S, et al. Acute respiratory distress syndrome: new definition, current and future therapeutic options. *J Thorac Dis*. 2013;5(3):326-334. doi:10.3978/j.issn.2072-1439.2013.04.05.

Food and Drug Administration (FDA). COVID-19: Developing drugs and biological products for treatment or prevention, guidance for industry, May 2020. Accessed November 11, 2020. https://www.fda.gov/regulatory-information/search-fda-guidance-documents/covid-19-developing-drugs-and-biological-products-treatment-or-prevention.

Garibaldi BT, Fiksel J, Muschelli J, et al. Patient trajectories among persons hospitalized for COVID-19: a cohort study. *Ann Intern Med*. Published online September 22, 2020. doi:10.7326/M20-3905.

Gloire G, Legrand-Poels S, Piette J. NF-kappaB activation by reactive oxygen species: fifteen years later. *Biochem Pharmacol*. 2006;72(11):1493-1505. doi:10.1016/j.bcp.2006.04.011.

Grunewald ME, Fehr AR, Athmer J, Perlman S. The coronavirus nucleocapsid protein is ADP-ribosylated. *Virology*. 2018;517:62-68. doi:10.1016/j.virol.2017.11.020.

Hillienhof A. COVID-19: What immune reactions are triggered. *German Medical Journal (Deutsches Ärzteblatt)*. 2020;117(39):A-1822.

Ke Y, Wang C, Zhang J, et al. The role of PARPs in inflammation-and metabolic-related diseases: molecular mechanisms and beyond. *Cells*. 2019;8(9):1047. doi:10.3390/cells8091047.

Khomich OA, Kochetkov SN, Bartosch B, Ivanov AV. Redox biology of respiratory viral infections. *Viruses*. 2018;10(8):392. doi:10.3390/v10080392.

Levey AS, Stevens LA, Schmid CH, et al. A new equation to estimate glomerular filtration rate. *Ann Intern Med*. 2009;150(9):604-612.

Levin AT, Hanage WP, Owusu-Boatey N, et al. Assessing the age specifity of infection fatality rates for COVID-19: systematic review, meta-analysis, and public policy implications. *Eur J Epidemiol*. 2020;35(12):1123-1138. doi: 10.1007/s10654-020-00698-1.

Rothan HA, Byrareddy SN. The epidemiology and pathogenesis of coronavirus disease (COVID-19) outbreak. *J Autoimmun*. 2020;109:102433. Published online February 26, 2020. doi:10.1016/j.jaut.2020.102433.

Schumann S, Kaiser A, Nicoletti F, et al. Immune-modulating drug MP1032 with SARS-CoV-2 antiviral activity in vitro: a potential multi-target approach for prevention and early intervention treatment of COVID-19. *Int J Mol Sci*. 2020;21(22):8803. doi:10.3390/ijms21228803.

Varga Z, Flammer AJ, Steiger P, et al. Endothelial cell infection and endotheliitis in COVID-19. *Lancet*. 2020;395(10234):1417-1418. Published online April 21, 2020. doi:10.1016/S0140-6736(20)30937-5.

WHO Rapid Evidence Appraisal for COVID-19 Therapies (WHO-REACT) Working Group, Sterne JAC, Murthy S, Diaz JV, et al. Association between administration of systemic corticosteroids and mortality among critically ill patients with COVID-19: a meta-analysis. *JAMA*. Published online September 2, 2020. 324(13):1-13. doi:10.1001/jama.2020.17023.

World Health Organization (WHO). 2020a. January 12, 2020. Accessed January 14, 2021. https://www.who.int/csr/don/12-january-2020-novel-coronavirus-china/en/.

World Health Organization (WHO). 2020b. Estimating mortality from COVID-19. Scientific brief, August 4, 2020. Accessed October 27, 2020. https://www.who.int/publications/i/item/WHO-2019-nCoV-Sci-Brief-Mortality-2020.1

World Health Organization (WHO). 2020c. Therapeutics and COVID-19. Living Guideline. December 17, 2020. Accessed December 17, 2020. https://www.who.int/publications/i/item/therapeutics-and-covid-19-living-guideline. WHO reference number: WHO/2019-nCoV/therapeutics/2020.1.

World Health Organization (WHO). 2020d. WHO R&D Blueprint. Novel coronavirus: COVID-19 therapeutic trial synopsis. February 18, 2020. Accessed October 19, 2020. https://www.who.int/publications-detail/covid-19-therapeutic-trial-synopsis.

This document is confidential.

18 APPENDICES

APPENDIX 1 describes the Charlson Comorbidity Index (CCI) applicable for this study.

APPENDIX 2 describes the EuroQol-5D-5L (EQ-5D-5L) questionnaire applicable for this study.

APPENDIX 1. CHARLSON COMORBIDITY INDEX

https://orthotoolkit.com/charlson-comorbidity-index/

Myocardial infarction (history of)

☑ No (+0)	☐ Yes (+1)

Congestive heart failure

☑ No (+0)	☐ Yes (+1)

Peripheral vascular disease

☑ No (+0)	☐ Yes (+1)

Cerebrovascular disease

☑ No (+0)	☐ Yes (+1)

Dementia

☑ No (+0)	☐ Yes (+1)

Chronic pulmonary disease

☑ No (+0)	☐ Yes (+1)

Rheumatologic disease

☑ No (+0)	☐ Yes (+1)

Peptic ulcer disease

☑ No (+0)	☐ Yes (+1)

Liver disease

☑ No (+0)	☐ Mild liver disease (+1)	☐ Moderate or severe liver disease (+3)

Diabetes

☑ No (+0)	☐ Diabetes without chronic complications (+1)	☐ Diabetes with chronic complications (+2)

Hemiplegia or paraplegia

☑ No (+0)	☐ Yes (+2)

Renal disease

☑ No (+0)	☐ Yes (+2)

Malignancy

☑ No (+0)	☐ Any malignancy, including leukemia and lymphoma (+2)	☐ Metastatic solid tumor (+6)

AIDS/HIV

☑ No (+0)	☐ Yes (+6)

Charlson Comorbidity Index Score: 0 / 29 = 0.0 %

APPENDIX 2. EUROQOL-5D-5L (EQ-5D-5L) QUESTIONNAIRE

Under each heading, please tick the ONE box that best describes your health TODAY.

MOBILITY

I have no problems in walking about	❑
I have slight problems in walking about	❑
I have moderate problems in walking about	❑
I have severe problems in walking about	❑
I am unable to walk about	❑

SELF-CARE

I have no problems washing or dressing myself	❑
I have slight problems washing or dressing myself	❑
I have moderate problems washing or dressing myself	❑
I have severe problems washing or dressing myself	❑
I am unable to wash or dress myself	❑

USUAL ACTIVITIES *(e.g. work, study, housework, family or leisure activities)*

I have no problems doing my usual activities	❑
I have slight problems doing my usual activities	❑
I have moderate problems doing my usual activities	❑
I have severe problems doing my usual activities	❑
I am unable to do my usual activities	❑

PAIN / DISCOMFORT

I have no pain or discomfort	❑
I have slight pain or discomfort	❑
I have moderate pain or discomfort	❑
I have severe pain or discomfort	❑
I have extreme pain or discomfort	❑

ANXIETY / DEPRESSION

I am not anxious or depressed	❑
I am slightly anxious or depressed	❑
I am moderately anxious or depressed	❑
I am severely anxious or depressed	❑
I am extremely anxious or depressed	❑

Sample

- We would like to know how good or bad your health is TODAY.
- This scale is numbered from 0 to 100.
- 100 means the best health you can imagine.
  0 means the worst health you can imagine.
- Mark an X on the scale to indicate how your health is TODAY.
- Now, please write the number you marked on the scale in the box below.

YOUR HEALTH TODAY = [ ]

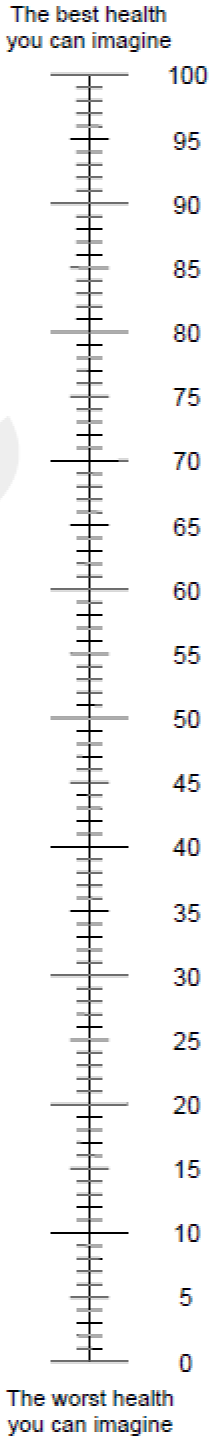

Sample
